# Machine Learning in Sensory Analysis of Mead—A Case Study: Ensembles of Classifiers

**DOI:** 10.3390/molecules30153199

**Published:** 2025-07-30

**Authors:** Krzysztof Przybył, Daria Cicha-Wojciechowicz, Natalia Drabińska, Małgorzata Anna Majcher

**Affiliations:** 1 Faculty of Food Science and Nutrition, Poznań University of Life Sciences, Wojska Polskiego 31, 60-624 Poznań, Poland; krzysztof.przybyl@up.poznan.pl (K.P.); daria.cicha@up.poznan.pl (D.C.-W.); natalia.drabinska@up.poznan.pl (N.D.); 2 Department of Biosystems Engineering, Poznań University of Life Sciences, Wojska Polskiego 50, 60-627 Poznań, Poland

**Keywords:** machine learning, ensembles of classifiers, mead aroma, sensory analysis, odor-active compounds

## Abstract

The aim was to explore using machine learning (including cluster mapping and k-means methods) to classify types of mead based on sensory analysis and aromatic compounds. Machine learning is a modern tool that helps with detailed analysis, especially because verifying aromatic compounds is challenging. In the first stage, a cluster map analysis was conducted, allowing for the exploratory identification of the most characteristic features of mead. Based on this, k-means clustering was performed to evaluate how well the identified sensory features align with logically consistent groups of observations. In the next stage, experiments were carried out to classify the type of mead using algorithms such as Random Forest (RF), adaptive boosting (AdaBoost), Bootstrap aggregation (Bagging), K-Nearest Neighbors (KNN), and Decision Tree (DT). The analysis revealed that the RF and KNN algorithms were the most effective in classifying mead based on sensory characteristics, achieving the highest accuracy. In contrast, the AdaBoost algorithm consistently produced the lowest accuracy results. However, the Decision Tree algorithm achieved the highest accuracy value (0.909), demonstrating its potential for precise classification based on aroma characteristics. The error matrix analysis also indicated that acacia mead was easier for the algorithms to identify than tilia or buckwheat mead. The results show the potential of combining an exploratory approach (cluster map with the k-means method) with machine learning. It is also important to focus on selecting and optimizing classification models used in practice because, as the results so far indicate, choosing the right algorithm greatly affects the success of mead identification.

## 1. Introduction

Honey has been a valued food product for centuries, renowned for its exceptional taste, aroma, and health benefits. As the only available sweetener, honey has been an essential part of our diet since the beginning of Homo sapiens [1,2]. Its diversity is due to different nectar sources, natural environments, production techniques, and fermentation processes. Depending on the type of plant from which bees collect nectar, honey can take on other colors, flavors, and aromas, making it a unique product in each region. The processes of fermentation and storage of honey affect its final properties, including texture, shelf life, and nutritional content. The main alcoholic beverage prepared from honey is mead, known for its unique and distinctive aroma and taste. The literature points to a number of research efforts to better understand and classify mead. The research by the team of Cicha-Wojciechowicz et al. (2024) focused on the effects of fermentation techniques and honey type on mead sensory profile [3], while the team of Pereira et al. (2019) studied the composition of volatile aromatic compounds [4]. Furthermore, Starowicz and Granvogl (2022) analyzed the effects of heat treatment on wort aroma changes [5], which is an important step in understanding the technological processes that influence the quality of this product. These research efforts help to know how different factors in different types of honey can affect the sensory and chemical properties of mead. Nowadays, approaches using modern analytical technologies and machine learning methods are becoming crucial in the systematic analysis of honey.

The application of machine learning methods to the analysis of mead offers new possibilities for classifying honey types [6,7,8], identifying aromatic compounds, and conducting future experiments in the optimization of production processes. Machine learning algorithms such as classifiers based on supervised learning, including Random Forest (RF) [9,10], adaptive boosting (AdaBoost), Bootstrap aggregation (Bagging), K-Nearest Neighbors (KNN), and decision tree (DT) [11,12,13], among others, can be applied to the analysis of chemical and sensory datasets [14,15]. DTs are some of the most widely used algorithms in data analysis, which allow the creation of simple yet explainable decision models. With DT, it is possible to both classify and predict the properties of food products based on the various descriptors identified in a given research question. In a transparent and easy-to-interpret way [16], DTs can be useful in the data analysis process, where the result must be understandable and practical for DTs to serve as a tool for implementation in the production process. In combination with other methods, such as RF or AdaBoost, decision trees form so-called ensembles of classifiers. This approach helps to improve the efficiency of classification and can be a crucial tool for improving mead production processes, assessing mead quality, and distinguishing between different types of mead based on sensory and chemical data characteristics.

In order to improve the performance of the models, an optimization technique was applied by tuning the hyperparameters using the GridSearch method, which makes it possible to effectively identify relevant features of the data, even in the case of high complexity. This type of optimization not only improves the generalizability of the model, but also significantly increases its efficiency. As pointed out by Liu et al. (2021) [17], machine learning algorithms can exhibit poor performance due to inappropriate selection of hyperparameters. Optimization of hyperparameters becomes crucial for achieving better model performance [18].

The aim of this research was to investigate the possibility of using machine learning to classify mead types based on their sensory analysis, particularly employing cluster mapping and k-means methods to identify relationships between mead groups based on their sensory characteristics. This approach will not only allow an accurate classification of mead but also improves understanding of the factors that influence its quality.

Given the challenges in verifying aromatic compounds in mead, machine learning provides an innovative way to conduct more detailed and accurate analyses. With algorithms that can recognize patterns in large datasets, it becomes possible to understand how variables like nectar origin, production methods, and fermentation influence the mead’s sensory characteristics.

## 2. Results and Discussion

### 2.1. Hierarchical Analysis of Mead Aroma Compounds

**Figure 1:**
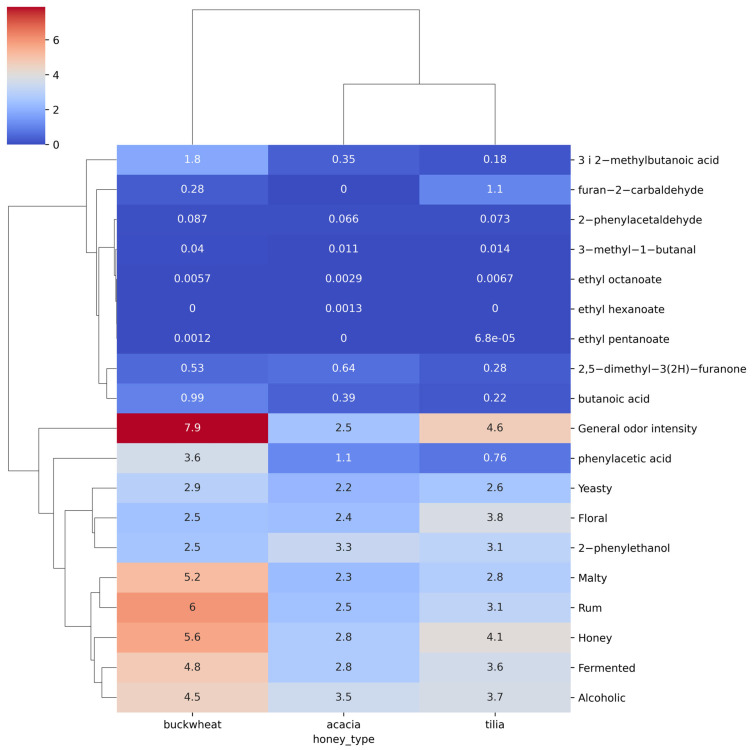
Cluster map of odor descriptors against compound concentrations.

A cluster map of odor-related characteristics and odor compound concentration characteristics was generated (Figure 1). It was found that one variable had the highest value relative to the others. This was the odor sensory attribute, General Odor Intensity, which was the focus of the next stage of the experiments. Based on this analysis, combinations were made to create learning sets followed by a model mead classification process. It was also observed that for the ‘buckwheat’ type of mead, in addition to General Odor Intensity, sensory attributes such as Malty, Rum, Honey, Fermented, and Alcoholic achieved high scores. For comparison, the sensory attributes listed for the other types of tilia and acacia mead were also higher for buckwheat honey than for the other attributes. This means that these sensory aroma trait variables were more dominant than the aroma compound concentration traits. This made it possible to accurately understand and prepare classification models for the selected aroma-sensory attributes. It was also found that the dominant decision class for mead would be the variable responsible for the type of buckwheat.

### 2.2. Interpretation of K-Means of Honey Aroma Compounds

**Figure 2:**
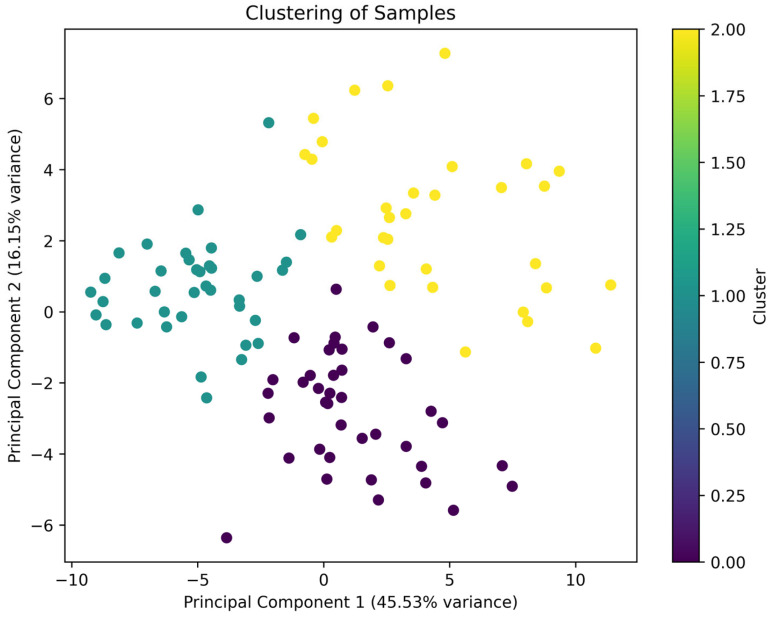
K-means for descriptors: General_odor_intensity, Honey, Malty, Yeasty, Fermented, Alcoholic, Floral, and Rum.

In the next stage of research, k-means analysis (Figure 2) was carried out between the descriptors (odor sensory attributes) identified from the cluster map patterns. Given the number and diversity of aromatic compounds, classifying mead types based on sensory characteristics is complex and necessitates statistical analysis. For this purpose, a multidimensional analysis method, specifically the k-means technique, was utilized. Based on previous research experience, the focus was on dividing sensory characteristics to evaluate the similarity levels among different groups of characteristics within a cluster. Attention was also given to how closely the sensory characteristics of mead resemble each other within a single group.

In Table 1, it can be observed that cluster 2 had the highest value of variance for the senor trait General Odor Intensity. This was confirmed from the cluster map analysis that General Odor Intensity strongly stands out in between cases in this cluster. It is possible that this descriptor plays a key role in identifying the type of mead. In this cluster, the General Odor Intensity interaction may facilitate the clustering of cases for other sensory attributes, which is reflected in the high values for other sensory attributes. For cluster 1, the sensory traits of mead aroma showed low intensity among the characteristics. This means that for cluster 1, the learning cases do not distinguish between the characteristics. The highest variance value in cluster 1 is also determined by the General Odor Intensity, whose mean value was 3.21. Cluster 0 gave an intermediate value between cluster 0 and cluster 1, suggesting that the General Odor Intensity descriptor was moderately expressed in the cases from this cluster. The learning cases in cluster 0 may have been particularly characteristic of the moderate value of the aforementioned descriptor. In conclusion, General Odor Intensity indeed represented an important sensory property for mead classification.

### 2.3. Machine Learning

In the analysis carried out on the basis of 21 different combinations of sensory features on the input variables classifying the mead, different machine learning algorithms were evaluated for their performance based on four key measures: accuracy (Acc), precision (Precision), sensitivity (Recall), and the F1-score (Table 2). The results are presented on a test set using the Train and Test method. Practically, the model was validated on an independent test set. Additionally, metrics such as accuracy, precision, recall, and F1-score were used to assess the model’s performance. In machine learning, this is a common approach, known as simple validation [19,20]. This method is especially useful for smaller datasets.

The highest scores were obtained by the Random Forest (6 times) and KNN (6 times) algorithms. It can be concluded that these models were the most effective in classifying mead. It can be assumed that Random Forest, due to its use of multiple decision trees and the random feature selection technique, had a high resistance to overfitting. This translates into its stability and good performance in different configurations with mead-based features [21]. On the other hand, KNN, based on nearest neighbor voting, also achieved high performance, especially in the evaluation of the performance metrics of this model [7,22,23]. Research shows that Random Forest, especially when combined with suitable feature extraction methods, achieves excellent results (e.g., Acc above 93%) [8,11,21,24,25]. Employing voting schemes and different distance metrics can further enhance classification performance. However, it should be noted that KNN is more sensitive to an increase in the number of features and may be less computationally efficient with large datasets.

In summary, both Random Forest and KNN can be very effective in sensory classification tasks, but their advantage is due to different mechanisms of operation: RF from the strength of the tree ensemble and randomness, and KNN from local neighbor voting in feature space [24].

The rest of the machine learning algorithms such as Bagging (6 times), Naive Bayes (3 times), and Decision Tree (3 times) also performed well but were not able to outperform Random Forest and KNN in terms of stability of results for different combinations. Bagging, a technique that involves training multiple models on different data samples, showed very robust results, but slightly less stable than the other algorithms. Naive Bayes and Decision Tree, although effective, achieved higher results, but only for selected correlations.

However, the highest metric for assessing the effectiveness of the model was obtained with Decision Tree for the sensory features General Odor Intensity, Yeasty, and Floral, which reached an accuracy of 0.909, a precision of 0.929, a recall of 0.909, and an F1-score of 0.911. It was observed that when optimizing the algorithms with the Grid Search method for the most effective Decision Tree model, the value for the tree depth hyperparameter reached a score of 6. Depending on the combination of sensory features, the Grid Search method produced different results. This is due to the fact that for the Decision Tree model with data on these sensory features, the optimal tree depth was 6, whereas in other configurations, both for the Decision Tree and Random Forest models, values of less or more were obtained. The aim of this was to avoid overfitting the model, as an appropriate tree depth affects the ability of the model to generalize, and too much depth can lead to the model fitting the noise in the data rather than capturing the true relationships.

In contrast, the worst results were obtained with the AdaBoost algorithm. In fact, the AdaBoost model achieved the lowest accuracy value of 0.515 for aroma features such as General Odor Intensity, Rum, and Yeasty, indicating its weaker performance compared to other algorithms. AdaBoost, which is a boosting algorithm, aims to improve the accuracy of the classifier by iteratively increasing the weights of incorrect examples and creating new weak classifiers.

**Figure 3:**
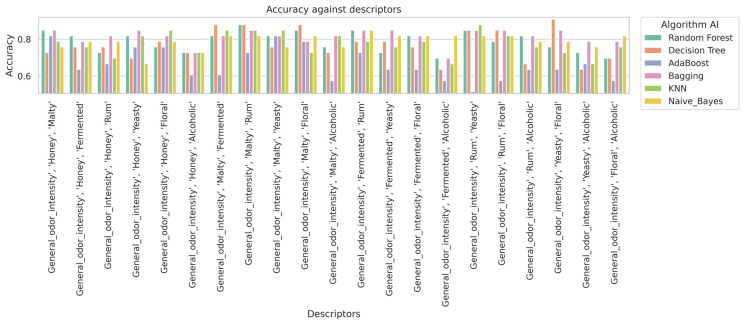
Impact of descriptors on algorithm accuracy.

**Figure 4:**
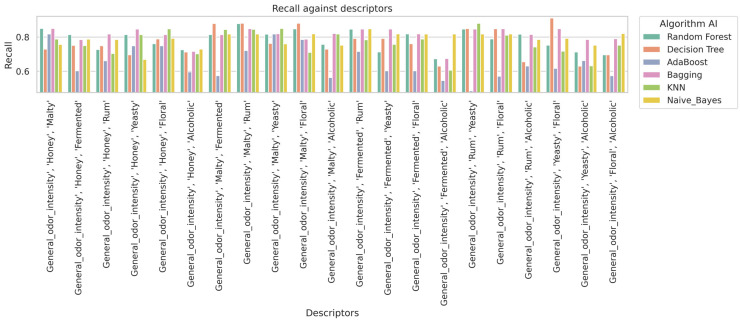
Impact of descriptors on algorithm recall.

**Figure 5:**
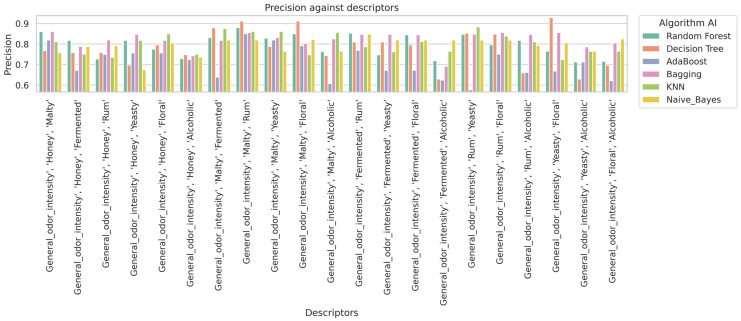
Impact of descriptors on algorithm precision.

**Figure 6:**
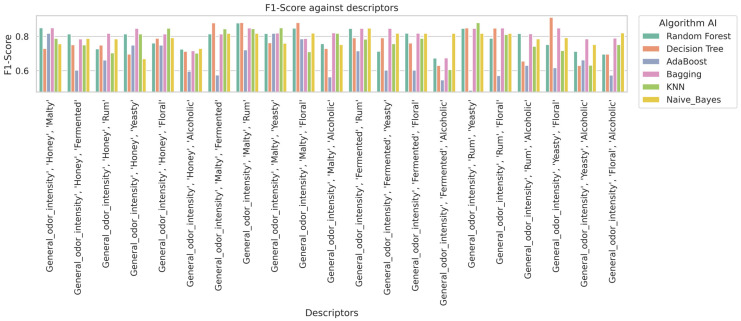
Impact of descriptors on algorithm F1-score.

In Figure 3, it is observed that the Random Forest and K-Nearest Neighbors (KNN) algorithms generally achieve the highest accuracy results in classifying mead types. In Figure 4, the Random Forest and KNN algorithms also demonstrate high performance in terms of recall rate. Overall, these algorithms also provide high precision (Figure 5) scores and F1-scores (Figure 6). However, it is worth noting that for specific sensory characteristics of mead, the Decision Tree algorithm obtained the highest single value for accuracy, recall, precision, and F1-score. The results clearly indicate that choosing an appropriate classification model significantly impacts the performance of the mead identification process in practical applications. Additionally, selecting suitable sensory descriptors is crucial for achieving optimal mead identification performance.

When tuning the models using the K-NN algorithm, it was observed that the ‘manhattan’ hyperparameter was the most frequently selected. The reason for this is that it gives a better assessment of model performance and stability [26]. In the case of the Random Forest algorithm, as in the case of the decision tree algorithm, the choice of hyperparameter was influenced by the avoidance of overfitting and, at the same time, the goal of obtaining high model performance. In the case of the Naive Bayes model, the Grid Search method determined the exact optimal value of var_smoothing equal to 1 × 10^−9^ to improve the stability of the model, as well as its generalizability, avoiding overfitting problems or computational errors associated with zero variance. In summary, the selection of different hyperparameter values using different machine learning algorithms made it possible to test on the basis of which sensory characteristics the model effectively classifies the type of mead. In light of the above and observations in the literature, it is worth investigating the selection of hyperparameters suitable for a specific task [27]. In the age of modern machine learning and deep learning tools, it allows that optimization through Grid Search has become rapid. Observations and the literature show that when choosing an optimization technique, it is also worth paying attention to the size of the dataset for a given problem [28].

The above information highlights the importance of selecting the appropriate machine learning algorithm and tuning hyperparameters for different honey types. Based on previous model training efforts, AdaBoost was identified as the least effective algorithm in this field. The literature also indicates that machine learning methods are relatively underused in sensory analysis [29]. Research by Schreurs et al. (2023) showed that one of the main challenges in classifying food based on sensory analysis is the large number of chemical compounds involved. It has also been noted that aromatic compounds vary in both chemical structure and concentration, which significantly complicates their classification or quantitative analysis [30].

Wang et al. (2022) demonstrated that machine learning can develop intelligent systems for sensory analysis of alcoholic beverages. These systems excel at detecting fraud and adulteration, analyzing aromatic profiles, overseeing production processes, and exploring links with human sensory perception [31]. Meanwhile, Yang et al. (2023) introduced a method combining sensory analysis and neural networks to predict consumers’ hedonic responses to fruit juice [32].

Nevertheless, the potential of artificial intelligence offers real opportunities for efficiently modeling complex data, especially in tools that monitor food products to ensure quality and safety. This is particularly relevant to the authors’ research on classifying different types of mead.

### 2.4. Analysis of Classifier Performance Based on Confusion Matrix Results

In machine learning, confusion matrices are an important tool for evaluating the performance of classifiers. Confusion matrices allow a detailed analysis of the predictive performance for a test set to be performed. It has also been observed in the literature that it is the most commonly used visualization to present among others this information [33,34,35,36,37]. It was found that it is usually presented to represent a single model. In our study, the recognition behavior of the selected mead type was also compared between models, which is crucial for selecting the appropriate model in a future implementation in an application [11,33].

In this experiment, the number of correct and incorrect classifications of mead into a particular class (buckwheat, acacia, tilia) was accurately determined. This analysis led to an understanding of the extent to which the model correctly classified learning instances related to aroma characteristics and where it went wrong. The confusion matrix also made it possible to identify specific difficulties that may have occurred with certain types of mead. Such a solution will be helpful in optimizing the model, which would lead to an assessment of the overall performance of the classifier, while attempting to improve these algorithms. A total of 126 confusion matrix plots were generated (Figures S1–S126), corresponding to 21 combinations of sensory odor features generated from the similarity map analysis and the k-means method. Each combination contained 6 models, and for each model, a confusion matrix was plotted for the test set. In each matrix, the class number corresponded to the type of mead, i.e., ‘1’ was acacia, ‘2’ was buckwheat, and ‘3’ was tilia. In order to explicitly identify individual types of mead, the classification analysis of the attributes ‘General_odor_intensity’, ‘Honey’, and ‘Malty’ from Table 1, Random Forest (Figure S1) and Bagging (Figure S4) successfully classified 11 cases for acacia and at the same time made an error in 1 case by assigning tilia to mead in the test set. In the class corresponding to buckwheat-based mead, 8 cases were classified in the class corresponding to this buckwheat, while 2 cases were incorrectly classified in the class corresponding to tilia. In the case of the last type of honey, 9 cases were clearly classified in favor of tilia honey, while only 2 cases were incorrectly classified in favor of tilia honey. In the second case (Table 3), for the characteristics ‘General_odor_intensity’, ‘Honey’, and ‘Fermented’, the Random Forest model was the most successful, classifying acacia honey in 11 cases, buckwheat-based mead in 9 cases, and tilia honey in 7 cases (Figure S7). In the third case (Table 3), considering the features ‘General_odor_intensity’, ‘Honey’, and ‘Rum’, the Bagging model was the one that correctly classified acacia honey in 11 cases, buckwheat-based mead in 9 cases, and tilia honey in 8 cases (Figure S16). The analysis of the confusion matrix between the models and the selection of sensory attributes showed that the algorithms were more successful in classifying acacia honey. In the case of buckwheat and tilia honey, this depended on the choice of model and the sensory characteristics of the mead. Considering the most effective Decision Tree model for the sensory attributes ‘General_odor_intensity’, ‘Yeasty’, and ‘Floral’ (Figures S109–S114), the confusion matrix on the test set correctly classified acacia honey in 10 cases, buckwheat-based mead in 9 cases, and lime tree honey in 11 cases (Figure S110). In fact, it can be seen that the difficulty in classifying mead is due to the type of mead, i.e., tilia honey, where in case 19 (Table 3), tilia honey was classified more correctly than acacia honey. In the group of characteristics related to ‘General_odor_intensity’, ‘Rum’, and ‘Yeasty’, where the AdaBoost model performed the worst (Figure S93), the KNN algorithm proved to be the most successful model, correctly classifying tilia mead in 11 cases as well as buckwheat and tilia mead in 9 cases (Figure S95).

## 3. Materials and Methods

### 3.1. Data Source

For the current research, data from a previously published study were used, including a quantitative olfactory profile analysis and the quantitation of important odor-active compounds for 12 types of mead. These meads were produced using three different honey varieties (acacia, buckwheat, and tilia) and three fermentation methods: spontaneous fermentation, inoculation with *Saccharomyces cerevisiae* yeast, and inoculation with *Galactomyces geotrichum* molds. Additionally, both heated and unheated wort variations were analyzed. The methods used for sample analysis were thoroughly described before [3]. In the presented study, 12 different mead types were investigated based on three honey types (acacia, buckwheat, and tilia) and four fermentations methods (spontaneous fermentation, inoculation with *Galactomyces geotrichum* molds, inoculation of *Saccharomyces cerevisiae* yeast in unheated wort, and inoculation of *Saccharomyces cerevisiae* in heated wort). In accordance with the findings of the referenced study, the botanical origin of the honey used in mead production showed the most significant impact on the differences in the aromatic profiles of the twelve samples analyzed. Consequently, in the present investigation, all twelve mead samples were classified exclusively based on the type of honey used, regardless of the fermentation method applied. A total of four meads were prepared for each of the three honey types. All analyses were performed in triplicate.

### 3.2. Data Collection

The research examined a series of sensory attributes: ‘General Odor Intensity’, ‘Honey’, ‘Malty’, ‘Fermented’, ‘Rum’, ‘Yeasty’, ‘Floral’, and ‘Alcoholic’. A total of these attributes was created. This process facilitated the creation of multiple input variables sets used for training the cluster models. The combinations aimed to deepen the understanding of relationships between the different sensory characteristics and potentially enhance the performance of the algorithms. By varying these patterns, it was possible to better capture important features that influence the accuracy of mead classification. As a result, 21 learning sets were designed for which the input variables were 3 sensory characteristics. The output variable in each set was the type of honey (acacia, talia, buckwheat). The dataset consisted of 108 learning cases. The train_test_split function was used to split the set into a training set and a test set. This means that 30% of the learning cases belonged to the test set and 70% of the learning cases belonged to the training set. The selection of learning cases from the training set was performed by the aforementioned function, which automatically decides which cases should go into the learning set and which into the test set. The only criterion set by the user is the proportion that determines how much data is included in the training set and how much in the test set. A random seed (random_state) was also specified to ensure reproducibility of the distribution, allowing results to be replicated in future experiments.

### 3.3. Parameter Selection for Classification Models

In the design of algorithms using ensembles of classifiers, an appropriate architecture was developed for each model (Table 3), which was then used to classify the different types of mead. In addition, during the learning process, the selection of the hyperparameters of each model was automated in order to obtain only the best model in terms of the evaluation metrics. In this way, it was possible to optimize the performance of each classifier to obtain the best possible results in the classification of mead types. The automated hyperparameter selection process also ensured greater consistency and repeatability of results, eliminating the risk of errors due to manual calibration.

In view of the fact that machine learning algorithms often contain several hyperparameters, this study used the optimization of selected hyperparameters to assess the performance of the models [38]. Within the Random Forest model, a set of hyperparameters was selected that included different values for the maximum tree depth (max_depth) and the number of estimators (n_estimators). This allowed different configurations to be tested and the optimum balance between accuracy and model complexity to be found.

In the research question for decision trees, only the maximum tree depth (max_depth) was considered as a hyperparameter, with specific values tested (Table 1). These values were intended to strike a balance between the complexity of the tree and its ability to extract meaningful patterns from the data.

Two important variables were introduced for the AdaBoost model: the number of estimators (n_estimators) and the learning rate (learning_rate). These values were tested over a wide range to optimize the learning rate and the number of weak classifiers in the ensemble.

The K-Nearest Neighbors algorithm used a more complex parametric grid, including the number of neighbors (n_neighbors), the distance measure (metric) and the way the neighbors were weighted (weights) (Table 3). Each of these parameters affected classification accuracy and computation time, so different values were tested to select the best settings.

Bagging (param_grid_bag) considered the parameter n_estimators, which determines the number of estimators in the ensemble. Three different values were tested (Table 1) in order to find the optimal number of submodels that best influenced the classification performance.

In the case of the Naive Bayes model, the var_smoothing hyperparameter was used, which was responsible for adding a small value to the variance of the features to avoid the problem of dividing by zero. The tested values (Table 3) allowed to check the effect of this parameter on the stability and accuracy of the model.

### 3.4. Model Training and Testing

In the next step, the learning process of the designed ensembles of classifiers [39,40,41] was carried out in order to evaluate their performance in recognizing mead types. During validation of the test set, the quality of learning was assessed using evaluation metrics such as accuracy, precision, recall, and F1-score. A confusion matrix was also used to analyze the predicted results. The process of model estimation and learning was carried out using Python version 3.11.11 [42,43,44,45].

A virtual machine specifically configured for development was used to build machine learning models and generate drawings, ensuring stability, performance, and compatibility with modern programming tools. Below are the technical configuration details of the virtual machine used as follows:

- CPU: Intel Xeon 2.2 GHz.
- RAM: 12 GB.
- Operating system: Ubuntu 22.04.4 LTS (Jammy).
- Python: 3.11.11.

Furthermore, machine learning algorithms were created, and drawings were produced using Python with libraries like NumPy 2.0.2, Pandas 2.2.2, Matplotlib 3.10.0, and Scikit-learn in Google Colab.

### 3.5. Statistical Analysis

In the process of clustering the data using the sklearn.cluster library, the clustering for the cases defining the different sensory characteristics was plotted on a graph. In the next step, the PCA (Principal Component Analysis) algorithm was used to reduce the data to two principal components, allowing them to be visualized in a two-dimensional graph. The k-means algorithm [22,46] grouped the sensory features, and the results of the clustering were presented in a graph with separate color-coding for different groups. This visualization facilitated the evaluation of the structure of the learning instances corresponding to the sensory features and allowed us to understand the preparation of learning sets based on the three sensory features.

As part of the analysis of the sensory feature data, correlations between the different features were also determined. A cluster map of the sensory features and odors was created to identify feature similarities that could influence the classification results. Based on the cluster map results, it was possible to prepare learning sets for selected odor features.

## 4. Conclusions

Within the scope of our research, we identified mead on the basis of selected sensory characteristics of aroma, which allowed us to accurately classify this type of mead. By analyzing the performance of different classification algorithms, we found that Random Forest and K-Nearest Neighbors (KNN) algorithms proved to be the most effective models for mead recognition. Both models showed high performance, but it was the Decision Tree algorithm that achieved the highest accuracy value, suggesting its potential for accurate classification based on odor features. These results indicate that the choice of an appropriate classification model can significantly affect the performance of the mead identification process in practical applications. Analysis of the confusion matrix also showed that acacia honey was more easily identified by the algorithms than tilia or buckwheat mead. It is worth noting that the high performance of the Decision Tree algorithm influenced the fact that the number of classified cases using the confusion matrix method was higher for lime honey than for acacia honey. The highest model performance result was obtained using the Decision Tree for odor sensory attributes such as General Odor Intensity, Yeasty, Floral, which resulted in an accuracy value of 0.909, a precision of 0.929, a recall of 0.909 and an F1-score of 0.911.

Future research directions may focus on the optimization of mead production processes using machine learning methods, which open up new opportunities for experiments aimed at increasing the efficiency and quality of this beverage. A deeper understanding of the factors influencing the quality of mead both in terms of its chemical and sensory characteristics is also an important area for further analysis. In the future, it is also worth focusing on the selection and optimization of classification models used in practice, because, as the results so far have shown, the appropriate choice of algorithm significantly affects the effectiveness of mead identification. The difficulties in distinguishing between buckwheat and lime mead require special attention analysis of the confusion matrix has shown that they are classified less precisely than acacia honey, which may indicate the need for further research into improving the classification performance of these particular types of mead.

It is also worth highlighting the importance of developing interdisciplinary research that combines advanced methods of chemical analysis, sensory evaluation, and artificial intelligence. This approach fosters the creation of modern research tools and the advancement of knowledge on the quality and characteristics of food products, including mead.

## Figures and Tables

**Table 1:** Results of k-means for descriptors: General_odor_intensity, Honey, Malty, Yeasty, Fermented, Alcoholic, Floral, and Rum.

Cluster	General_odor_intensity	Honey	Malty	Yeasty	Fermented	Alcoholic	Floral	Rum
0	4.182051	4.125641	3.446154	4.164103	4.312821	4.997436	4.853846	4.164103
1	3.210811	2.435135	1.643243	0.943243	1.878378	2.097297	1.294595	1.513514
2	8.065625	6.165625	5.453125	2.468750	5.046875	4.656250	2.253125	6.203125

**Table 2:** Machine learning algorithms with the best hyperparameters.

No.	Descriptors	Algorithm AI	Best Hyperparameter
1	‘General_odor_intensity’, ‘Honey’, ‘Malty’	Random Forest	{‘max_depth’: 7, ‘n_estimators’: 500}
		Decision Tree	{‘max_depth’: 5}
		AdaBoost	{‘learning_rate’: 0.01, ‘n_estimators’: 50}
		Bagging	{‘n_estimators’: 100}
		KNN	{‘metric’: ‘chebyshev’, ‘n_neighbors’: 3}
		Naive_Bayes	{‘var_smoothing’: 1 × 10^−9^}
2	‘General_odor_intensity’, ‘Honey’, ‘Fermented’	Random Forest	{‘max_depth’: 6, ‘n_estimators’: 10}
		Decision Tree	{‘max_depth’: 6}
		AdaBoost	{‘learning_rate’: 0.01, ‘n_estimators’: 50}
		Bagging	{‘n_estimators’: 50}
		KNN	{‘metric’: ‘manhattan’, ‘n_neighbors’: 5}
		Naive_Bayes	{‘var_smoothing’: 1 × 10^−9^ }
3	‘General_odor_intensity’, ‘Honey’, ‘Rum’	Random Forest	{‘max_depth’: 3, ‘n_estimators’: 10}
		Decision Tree	{‘max_depth’: 5}
		AdaBoost	{‘learning_rate’: 0.01, ‘n_estimators’: 50}
		Bagging	{‘n_estimators’: 200}
		KNN	{‘metric’: ‘manhattan’, ‘n_neighbors’: 2}
		Naive_Bayes	{‘var_smoothing’: 1 × 10^−9^}
4	‘General_odor_intensity’, ‘Honey’, ‘Yeasty’	Random Forest	{‘max_depth’: 3, ‘n_estimators’: 100}
		Decision Tree	{‘max_depth’: 3}
		AdaBoost	{‘learning_rate’: 0.01, ‘n_estimators’: 50}
		Bagging	{‘n_estimators’: 200}
		KNN	{‘metric’: ‘chebyshev’, ‘n_neighbors’: 3}
		Naive_Bayes	{‘var_smoothing’: 1 × 10^−9^}
5	‘General_odor_intensity’, ‘Honey’, ‘Floral’	Random Forest	{‘max_depth’: 7, ‘n_estimators’: 50}
		Decision Tree	{‘max_depth’: 6}
		AdaBoost	{‘learning_rate’: 0.01, ‘n_estimators’: 50}
		Bagging	{‘n_estimators’: 100}
		KNN	{‘metric’: ‘Euclidean’, ‘n_neighbors’: 7}
		Naive_Bayes	{‘var_smoothing’: 1 × 10^−9^}
6	‘General_odor_intensity’, ‘Honey’, ‘Alcoholic’	Random Forest	{‘max_depth’: 7, ‘n_estimators’: 1000}
		Decision Tree	{‘max_depth’: 5}
		AdaBoost	{‘learning_rate’: 0.01, ‘n_estimators’: 100}
		Bagging	{‘n_estimators’: 100}
		KNN	{‘metric’: ‘minkowski’, ‘n_neighbors’: 2}
		Naive_Bayes	{‘var_smoothing’: 1 × 10^−9^}
7	‘General_odor_intensity’, ‘Malty’, ‘Fermented’	Random Forest	{‘max_depth’: 4, ‘n_estimators’: 50}
		Decision Tree	{‘max_depth’: 7}
		AdaBoost	{‘learning_rate’: 0.01, ‘n_estimators’: 50}
		Bagging	{‘n_estimators’: 50}
		KNN	{‘metric’: ‘manhattan’, ‘n_neighbors’: 3}
		Naive_Bayes	{‘var_smoothing’: 1 × 10^−9^}
8	‘General_odor_intensity’, ‘Malty’, ‘Rum’	Random Forest	{‘max_depth’: 7, ‘n_estimators’: 10}
		Decision Tree	{‘max_depth’: 5}
		AdaBoost	{‘learning_rate’: 0.1, ‘n_estimators’: 50}
		Bagging	{‘n_estimators’: 100}
		KNN	{‘metric’: ‘manhattan’, ‘n_neighbors’: 5}
		Naive_Bayes	{‘var_smoothing’: 1 × 10^−9^}
9	‘General_odor_intensity’, ‘Malty’, ‘Yeasty’	Random Forest	{‘max_depth’: 7, ‘n_estimators’: 50}
		Decision Tree	{‘max_depth’: 6}
		AdaBoost	{‘learning_rate’: 0.01, ‘n_estimators’: 50}
		Bagging	{‘n_estimators’: 200}
		KNN	{‘metric’: ‘minkowski’, ‘n_neighbors’: 3}
		Naive_Bayes	{‘var_smoothing’: 1 × 10^−9^}
10	‘General_odor_intensity’, ‘Malty’, ‘Floral’	Random Forest	{‘max_depth’: 6, ‘n_estimators’: 10}
		Decision Tree	{‘max_depth’: 4}
		AdaBoost	{‘learning_rate’: 0.01, ‘n_estimators’: 50}
		Bagging	{‘n_estimators’: 50}
		KNN	{‘metric’: ‘chebyshev’, ‘n_neighbors’: 3}
		Naive_Bayes	{‘var_smoothing’: 1 × 10^−9^}
11	‘General_odor_intensity’, ‘Malty’, ‘Alcoholic’	Random Forest	{‘max_depth’: 6, ‘n_estimators’: 10}
		Decision Tree	{‘max_depth’: 4}
		AdaBoost	{‘learning_rate’: 0.01, ‘n_estimators’: 50}
		Bagging	{‘n_estimators’: 50}
		KNN	{‘metric’: ‘manhattan’, ‘n_neighbors’: 3}
		Naive_Bayes	{‘var_smoothing’: 1 × 10^−9^}
12	‘General_odor_intensity’, ‘Fermented’, ‘Rum’	Random Forest	{‘max_depth’: 5, ‘n_estimators’: 50}
		Decision Tree	{‘max_depth’: 6}
		AdaBoost	{‘learning_rate’: 0.1, ‘n_estimators’: 500}
		Bagging	{‘n_estimators’: 100}
		KNN	{‘metric’: ‘Euclidean’, ‘n_neighbors’: 2}
		Naive_Bayes	{‘var_smoothing’: 1 × 10^−9^}
13	‘General_odor_intensity’, ‘Fermented’, ‘Yeasty’	Random Forest	{‘max_depth’: 4, ‘n_estimators’: 10}
		Decision Tree	{‘max_depth’: 6}
		AdaBoost	{‘learning_rate’: 0.01, ‘n_estimators’: 50}
		Bagging	{‘n_estimators’: 50}
		KNN	{‘metric’: ‘Euclidean’, ‘n_neighbors’: 5}
		Naive_Bayes	{‘var_smoothing’: 1 × 10^−9^}
14	‘General_odor_intensity’, ‘Fermented’, ‘Floral’	Random Forest	{‘max_depth’: 6, ‘n_estimators’: 100}
		Decision Tree	{‘max_depth’: 3}
		AdaBoost	{‘learning_rate’: 0.01, ‘n_estimators’: 50}
		Bagging	{‘n_estimators’: 100}
		KNN	{‘metric’: ‘manhattan’, ‘n_neighbors’: 5}
		Naive_Bayes	{‘var_smoothing’: 1 × 10^−9^}
15	‘General_odor_intensity’, ‘Fermented’, ‘Alcoholic’	Random Forest	{‘max_depth’: 3, ‘n_estimators’: 100}
		Decision Tree	{‘max_depth’: 3}
		AdaBoost	{‘learning_rate’: 0.01, ‘n_estimators’: 100}
		Bagging	{‘n_estimators’: 50}
		KNN	{‘metric’: ‘Euclidean’, ‘n_neighbors’: 2}
		Naive_Bayes	{‘var_smoothing’: 1 × 10^−9^}
16	‘General_odor_intensity’, ‘Rum’, ‘Yeasty’	Random Forest	{‘max_depth’: 7, ‘n_estimators’: 50}
		Decision Tree	{‘max_depth’: 7}
		AdaBoost	{‘learning_rate’: 1.0, ‘n_estimators’: 50}
		Bagging	{‘n_estimators’: 100}
		KNN	{‘metric’: ‘manhattan’, ‘n_neighbors’: 5}
		Naive_Bayes	{‘var_smoothing’: 1 × 10^−9^}
17	‘General_odor_intensity’, ‘Rum’, ‘Floral’	Random Forest	{‘max_depth’: 4, ‘n_estimators’: 500}
		Decision Tree	{‘max_depth’: 3}
		AdaBoost	{‘learning_rate’: 0.1, ‘n_estimators’: 500}
		Bagging	{‘n_estimators’: 100}
		KNN	{‘metric’: ‘chebyshev’, ‘n_neighbors’: 2}
		Naive_Bayes	{‘var_smoothing’: 1 × 10^−9^}
18	‘General_odor_intensity’, ‘Rum’, ‘Alcoholic’	Random Forest	{‘max_depth’: 5, ‘n_estimators’: 100}
		Decision Tree	{‘max_depth’: 3}
		AdaBoost	{‘learning_rate’: 0.01, ‘n_estimators’: 50}
		Bagging	{‘n_estimators’: 100}
		KNN	{‘metric’: ‘Euclidean’, ‘n_neighbors’: 3}
		Naive_Bayes	{‘var_smoothing’: 1 × 10^−9^}
19	‘General_odor_intensity’, ‘Yeasty’, ‘Floral’	Random Forest	{‘max_depth’: 6, ‘n_estimators’: 100}
		Decision Tree	{‘max_depth’: 6}
		AdaBoost	{‘learning_rate’: 0.01, ‘n_estimators’: 50}
		Bagging	{‘n_estimators’: 50}
		KNN	{‘metric’: ‘chebyshev’, ‘n_neighbors’: 3}
		Naive_Bayes	{‘var_smoothing’: 1 × 10^−9^}
20	‘General_odor_intensity’, ‘Yeasty’, ‘Alcoholic’	Random Forest	{‘max_depth’: 3, ‘n_estimators’: 50}
		Decision Tree	{‘max_depth’: 3}
		AdaBoost	{‘learning_rate’: 0.1, ‘n_estimators’: 50}
		Bagging	{‘n_estimators’: 50}
		KNN	{‘metric’: ‘chebyshev’, ‘n_neighbors’: 2}
		Naive_Bayes	{‘var_smoothing’: 1 × 10^−9^}
21	‘General_odor_intensity’, ‘Floral’, ‘Alcoholic’	Random Forest	{‘max_depth’: 7, ‘n_estimators’: 10}
		Decision Tree	{‘max_depth’: 3}
		AdaBoost	{‘learning_rate’: 0.01, ‘n_estimators’: 100}
		Bagging	{‘n_estimators’: 50}
		KNN	{‘metric’: ‘manhattan’, ‘n_neighbors’: 3}
		Naive_Bayes	{‘var_smoothing’: 1 × 10^−9^}

**Table 3:** The structure of hyperparameters in tuning algorithms for ensembles of classifiers.

Machine Learning Algorithm Type	Hyperparameters Used	Value
Decision Tree	max_depth	3, 4, 5, 6, 7
Random Forest	max_depth	3, 4, 5, 6, 7
Random Forest	n_estimators	10, 50, 100, 200, 500, 1000
AdaBoost	n_estimators	50, 100, 200, 500, 1000
AdaBoost	learning_rate	0.01, 0.1, 1.0
KNN	n_neighbors	2, 3, 5, 7
KNN	metric	Euclidean, manhattan, chebyshev, minkowski
KNN	weights	‘uniform’, ‘distance’
Bagging	n_estimators	50, 100, 200
Naive_Bayes	var_smoothing	1 × 10^−9^, 1 × 10^−8^, 1 × 10^−7^, 1 × 10^−6^, 1 × 10^−5^

## Data Availability

All original data presented in this research are included in the article. Further information is available from the first author or the corresponding author upon reasonable request.

## Supplementary Materials

The following supporting information can be downloaded from: https://www.mdpi.com/article/10.3390/molecules30153199/s1. Figures S1–S126: Confusion matrix of the classifier ensembles calculated on the test set for all sensory attributes of the odor.
