# Supplementary material for: Machine Learning in Sensory Analysis of Mead—A Case Study: Ensembles of Classifiers
Source: Molecules. 2025 Jul 30;30(15):3199. doi: 10.3390/molecules30153199 (PMC12348089; doi:10.3390/molecules30153199)
Supplement: Supplementary file 1 [file molecules-30-03199-s001.zip › molecules-3776098-supplementary.pdf]

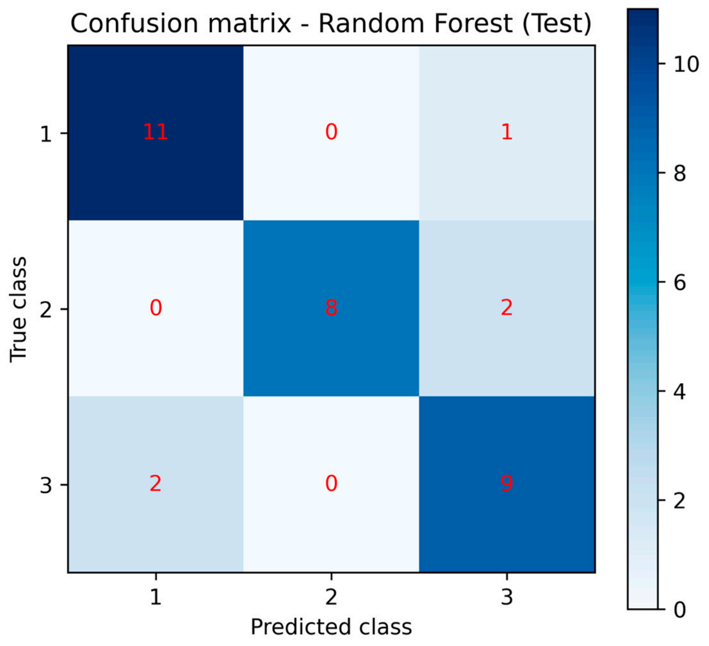

Figure S1. Confusion matrix of the classifier ensembles calculated on the test set for all sensory attributes of the odour

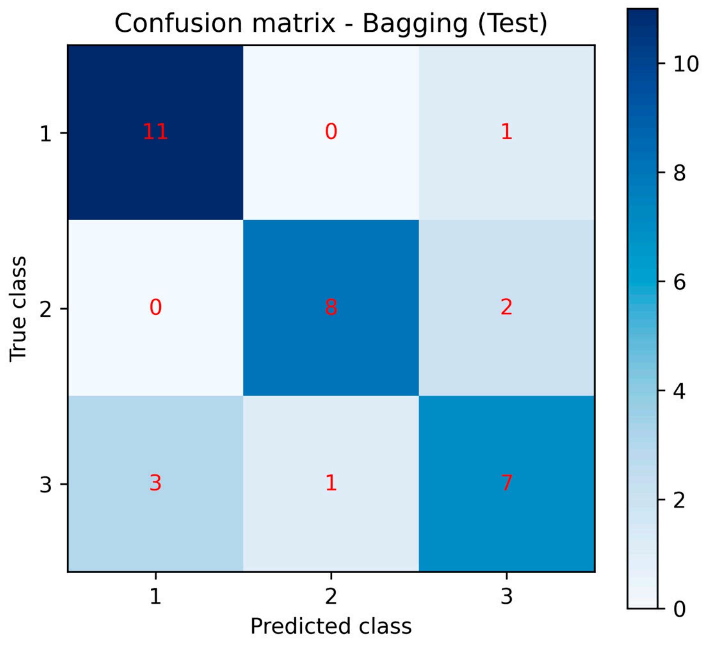

Figure S2. Confusion matrix of the classifier ensembles calculated on the test set for all sensory attributes of the odour

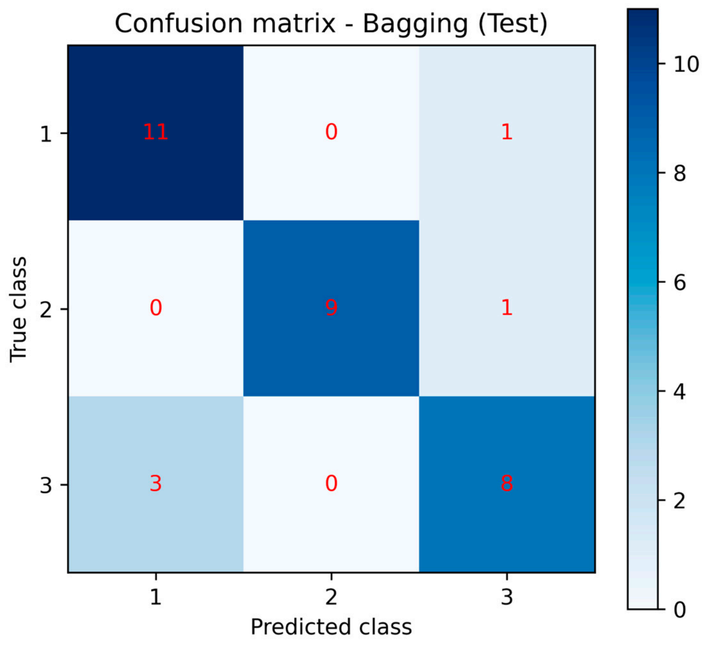

Figure S3. Confusion matrix of the classifier ensembles calculated on the test set for all sensory attributes of the odour

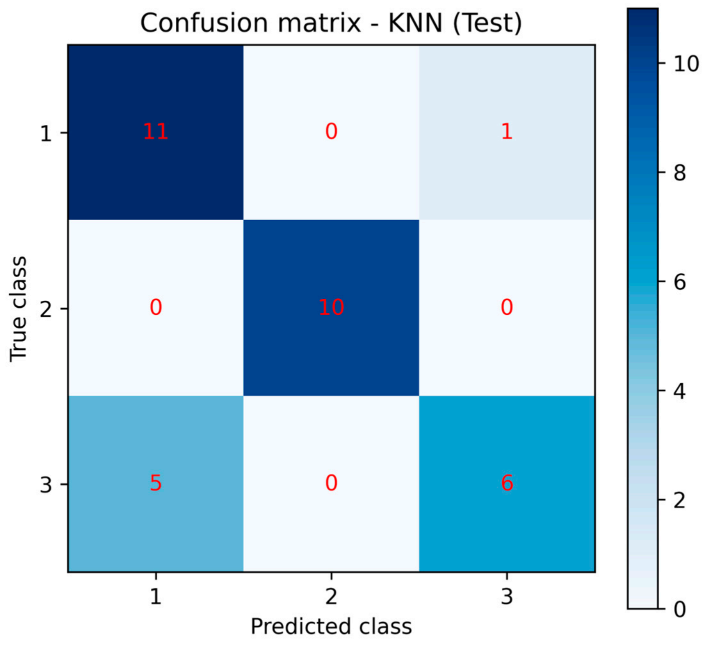

Figure S4. Confusion matrix of the classifier ensembles calculated on the test set for all sensory attributes of the odour

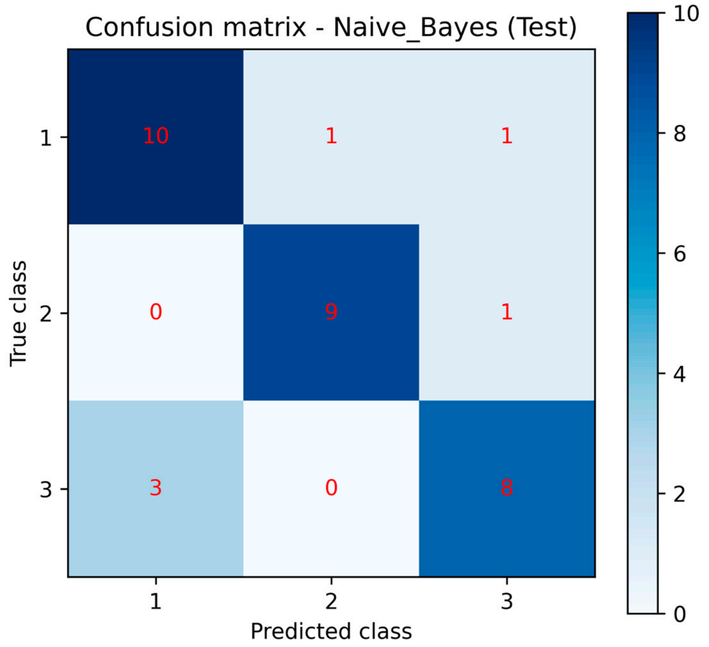

Figure S5. Confusion matrix of the classifier ensembles calculated on the test set for all sensory attributes of the odour

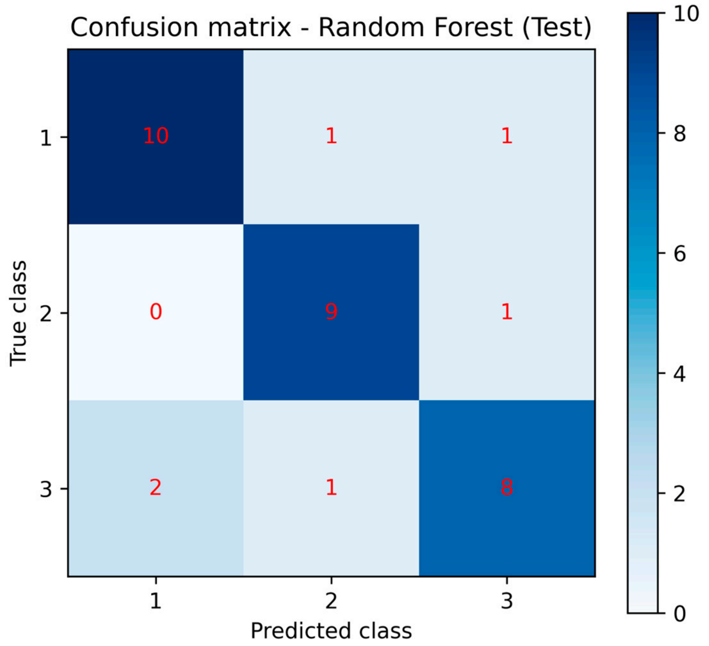

Figure S6. Confusion matrix of the classifier ensembles calculated on the test set for all sensory attributes of the odour

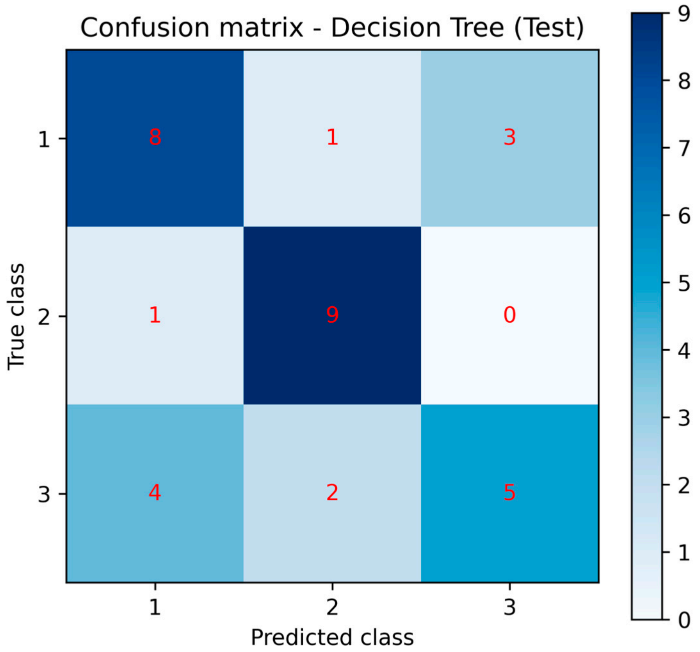

Figure S7. Confusion matrix of the classifier ensembles calculated on the test set for all sensory attributes of the odour

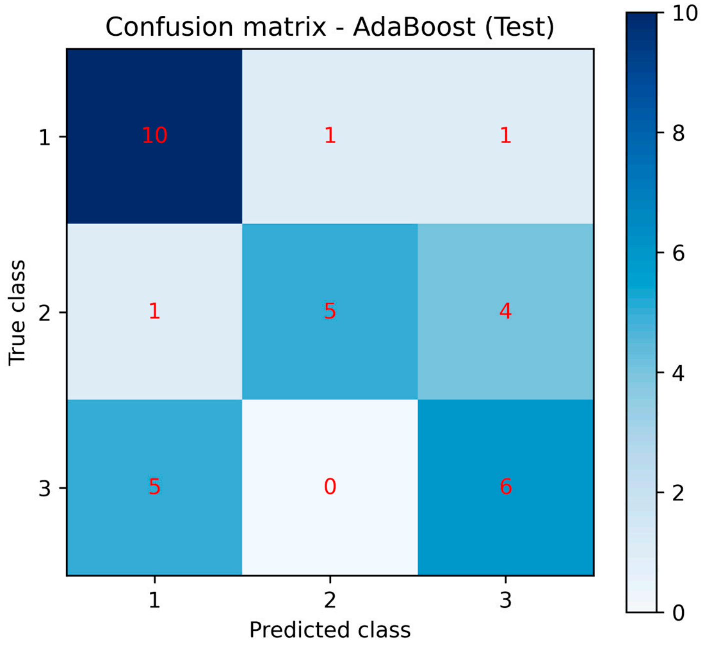

Figure S8. Confusion matrix of the classifier ensembles calculated on the test set for all sensory attributes of the odour

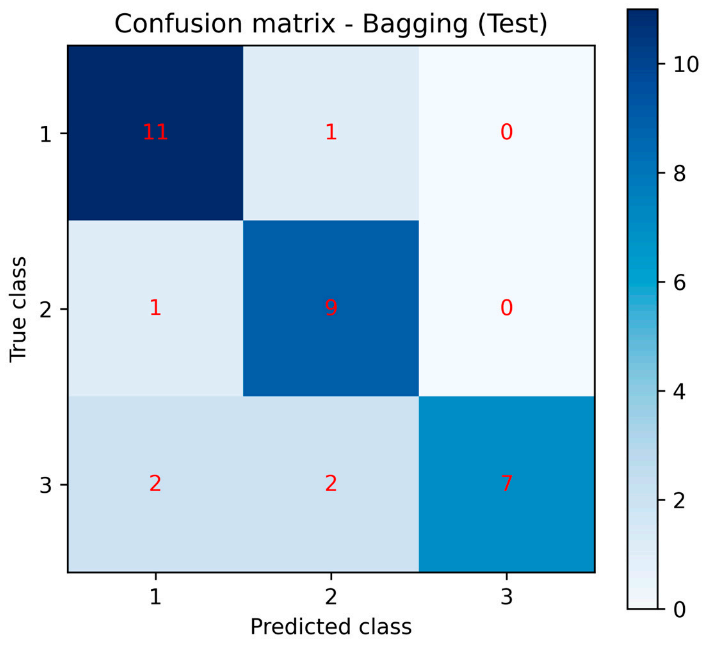

Figure S9. Confusion matrix of the classifier ensembles calculated on the test set for all sensory attributes of the odour

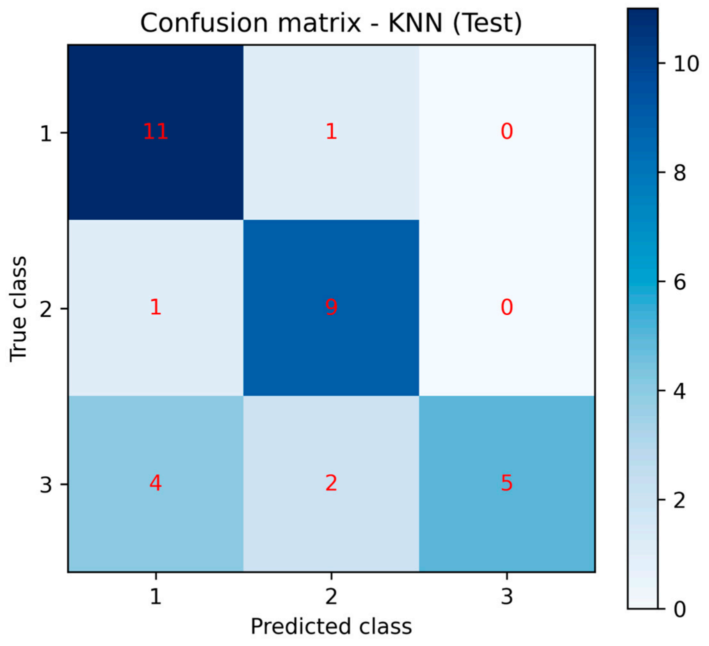

Figure S10. Confusion matrix of the classifier ensembles calculated on the test set for all sensory attributes of the odour

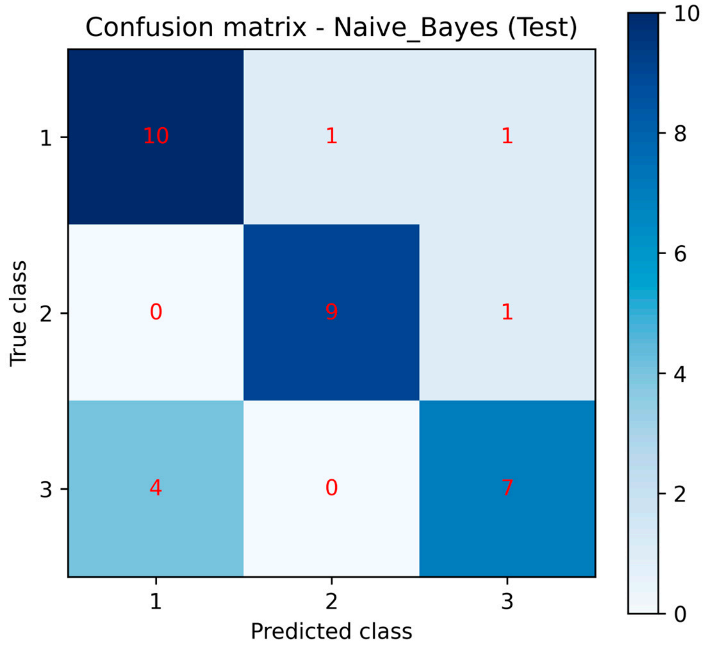

Figure S11. Confusion matrix of the classifier ensembles calculated on the test set for all sensory attributes of the odour

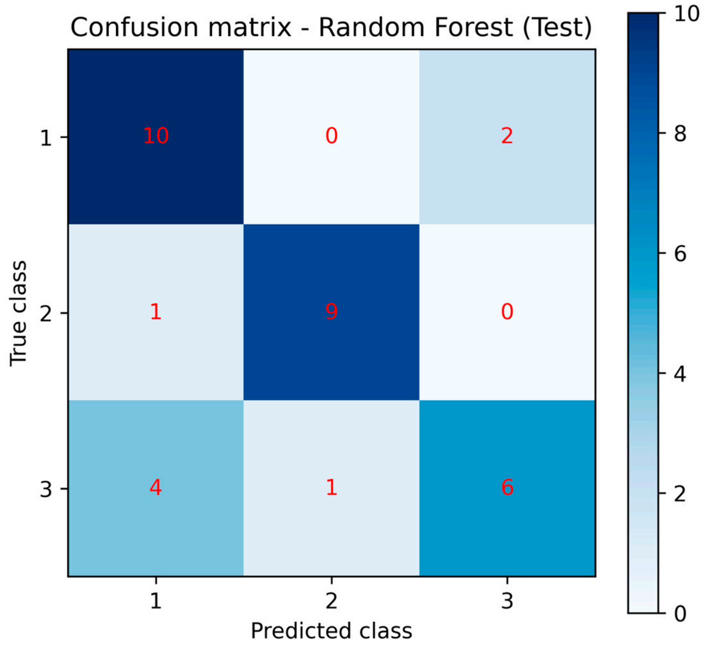

Figure S12. Confusion matrix of the classifier ensembles calculated on the test set for all sensory attributes of the odour

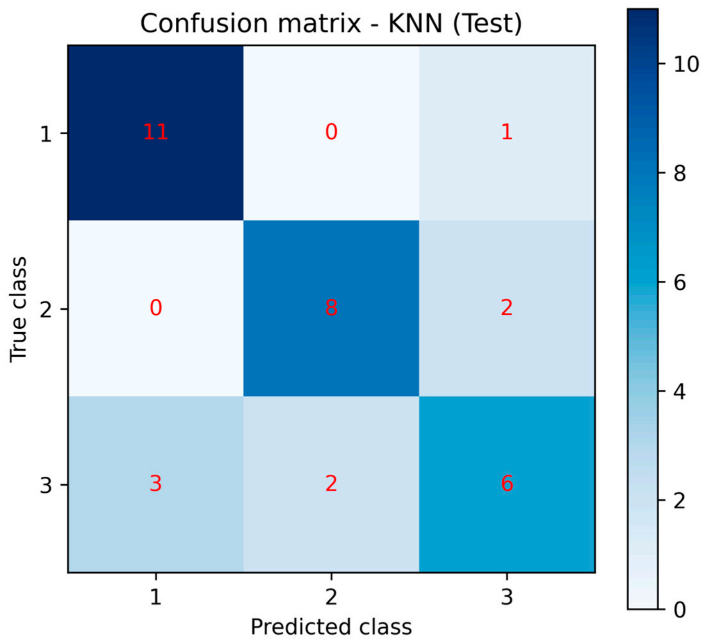

Figure S13. Confusion matrix of the classifier ensembles calculated on the test set for all sensory attributes of the odour

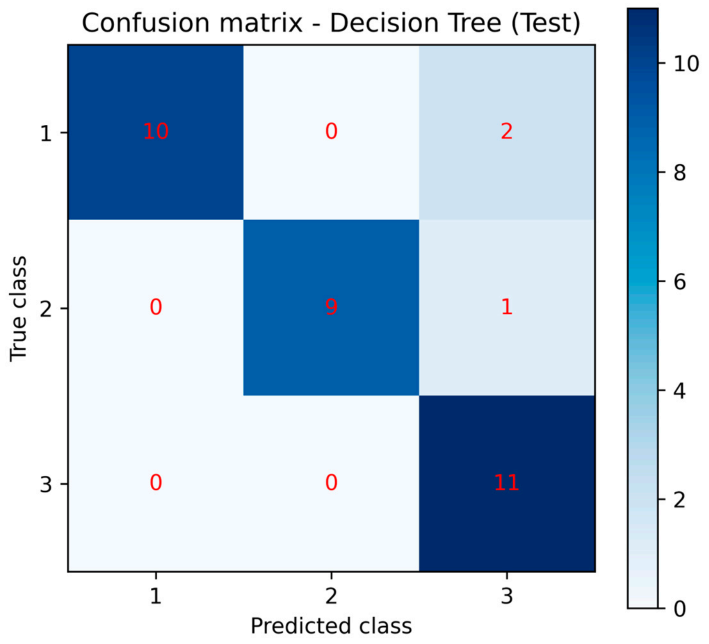

Figure S14. Confusion matrix of the classifier ensembles calculated on the test set for all sensory attributes of the odour

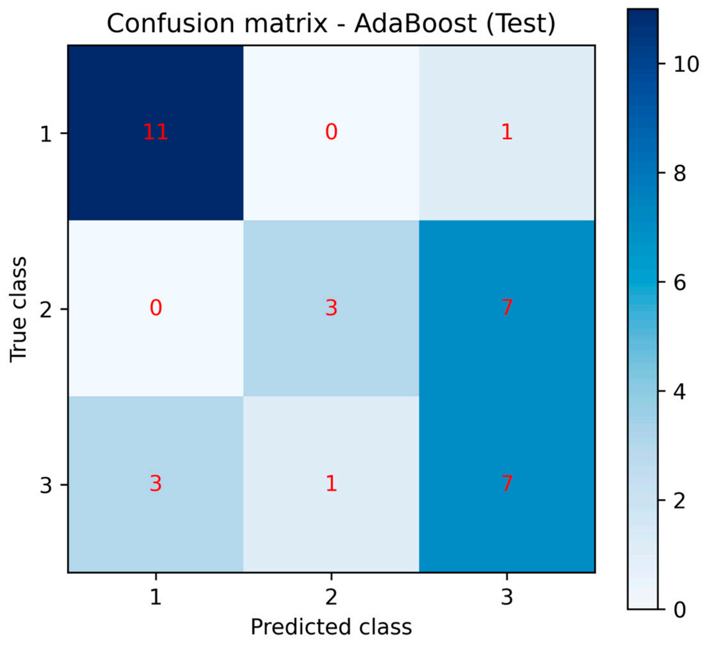

Figure S15. Confusion matrix of the classifier ensembles calculated on the test set for all sensory attributes of the odour

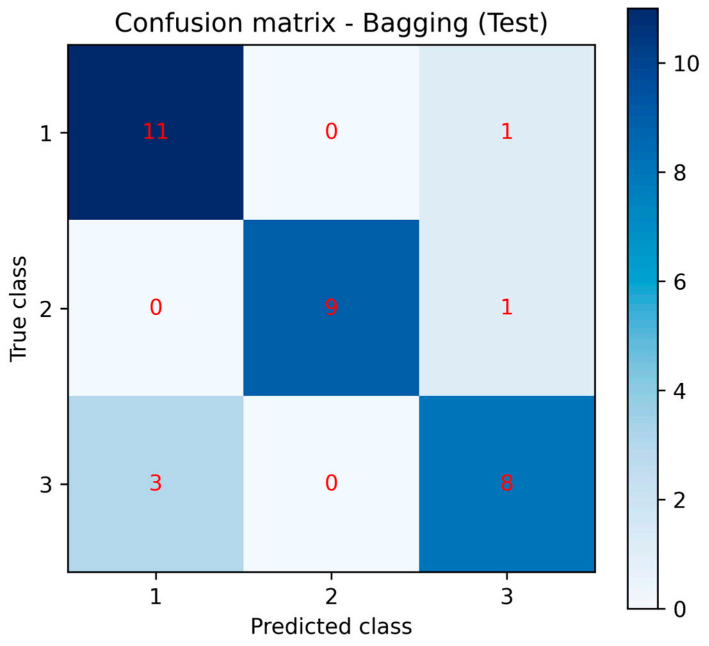

Figure S16. Confusion matrix of the classifier ensembles calculated on the test set for all sensory attributes of the odour

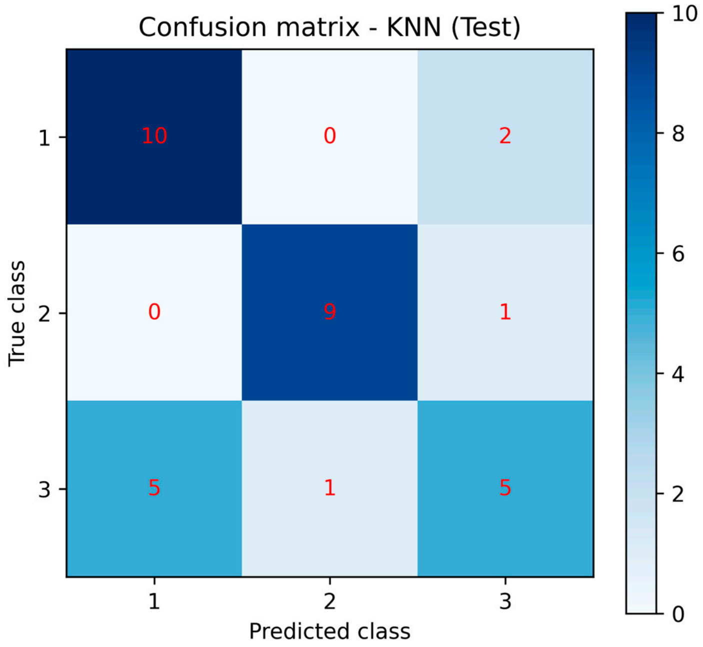

Figure S17. Confusion matrix of the classifier ensembles calculated on the test set for all sensory attributes of the odour

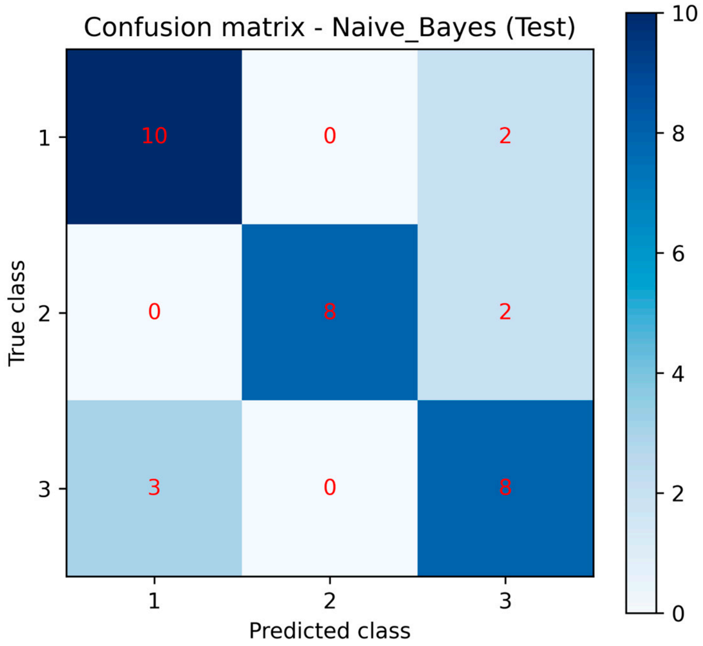

Figure S18. Confusion matrix of the classifier ensembles calculated on the test set for all sensory attributes of the odour

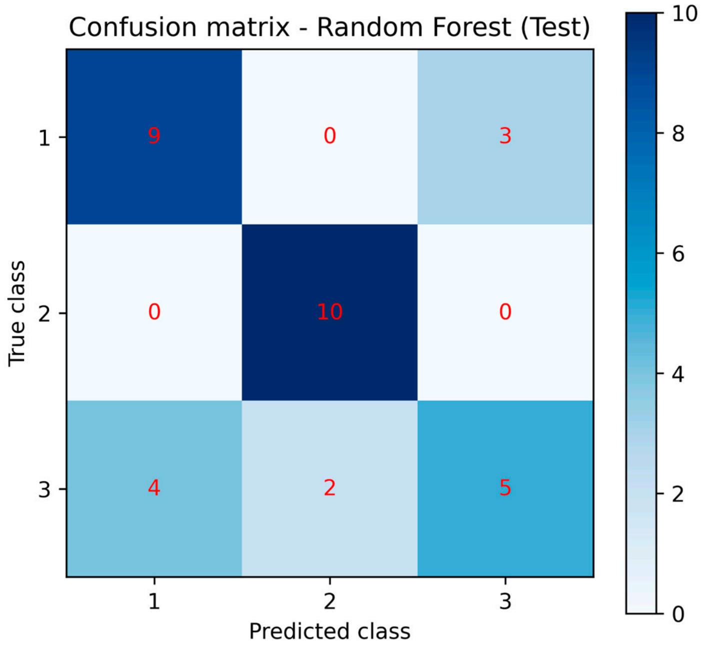

Figure S19. Confusion matrix of the classifier ensembles calculated on the test set for all sensory attributes of the odour

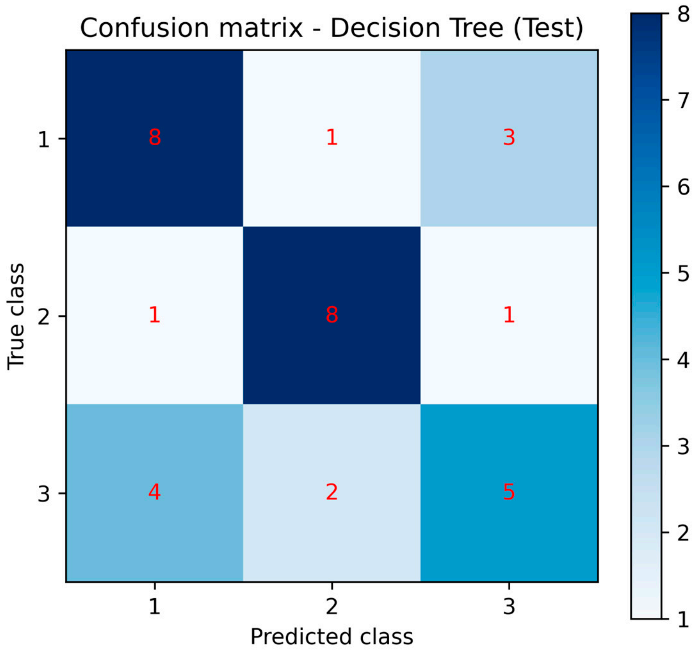

Figure S20. Confusion matrix of the classifier ensembles calculated on the test set for all sensory attributes of the odour

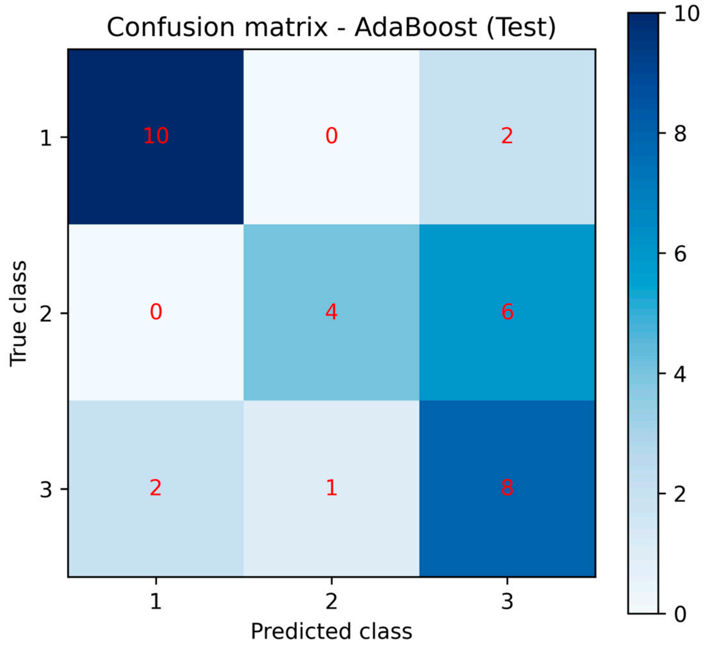

Figure S21. Confusion matrix of the classifier ensembles calculated on the test set for all sensory attributes of the odour

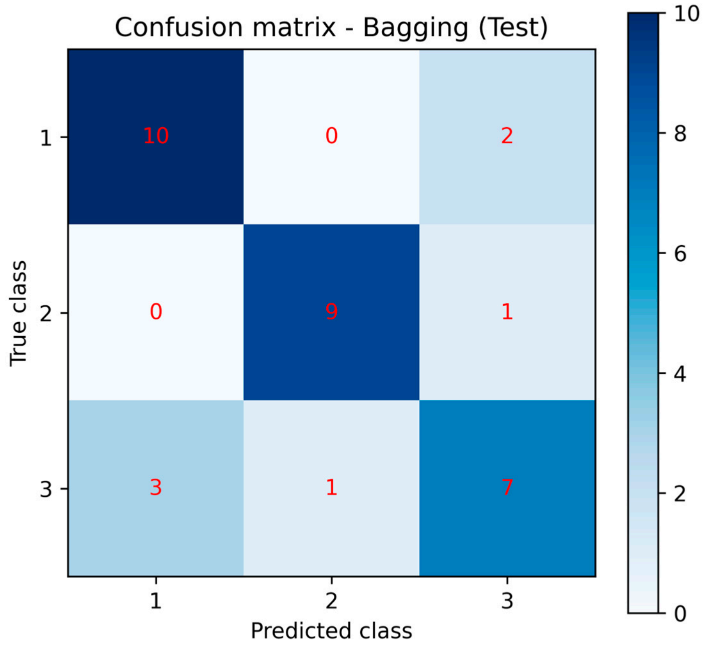

Figure S22. Confusion matrix of the classifier ensembles calculated on the test set for all sensory attributes of the odour

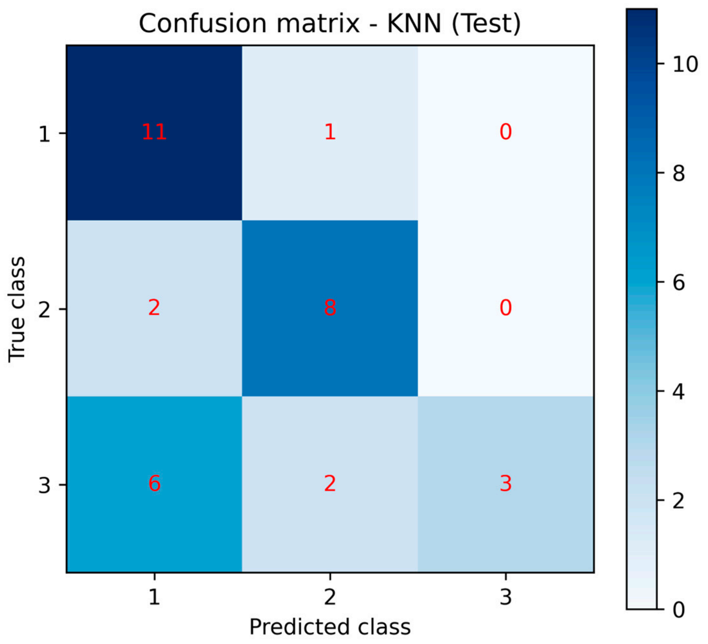

Figure S23. Confusion matrix of the classifier ensembles calculated on the test set for all sensory attributes of the odour

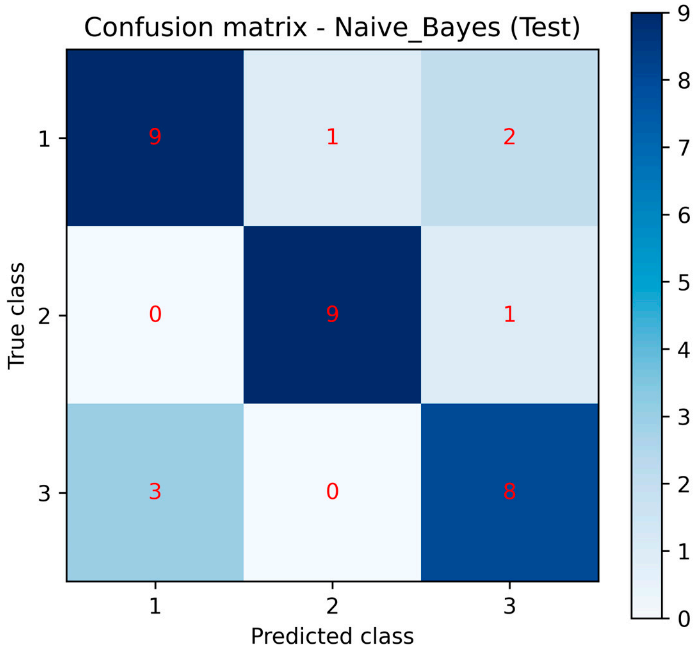

Figure S24. Confusion matrix of the classifier ensembles calculated on the test set for all sensory attributes of the odour

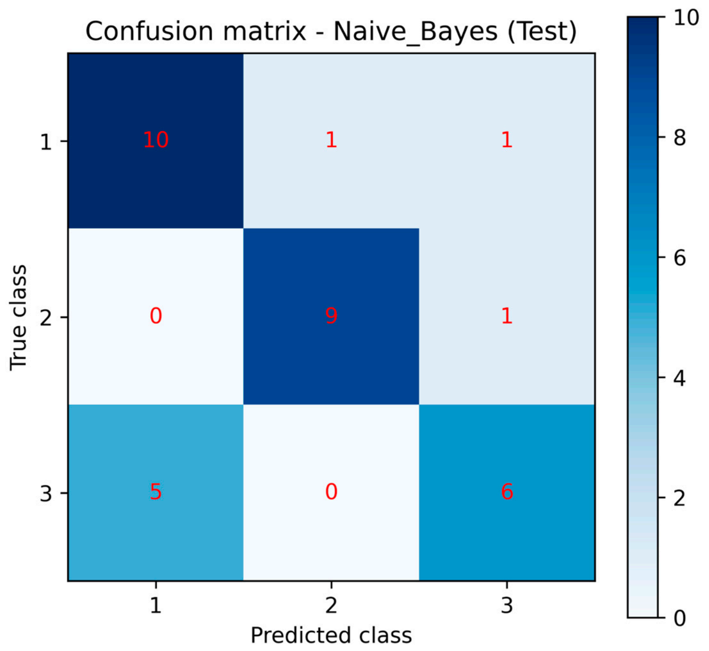

Figure S25. Confusion matrix of the classifier ensembles calculated on the test set for all sensory attributes of the odour

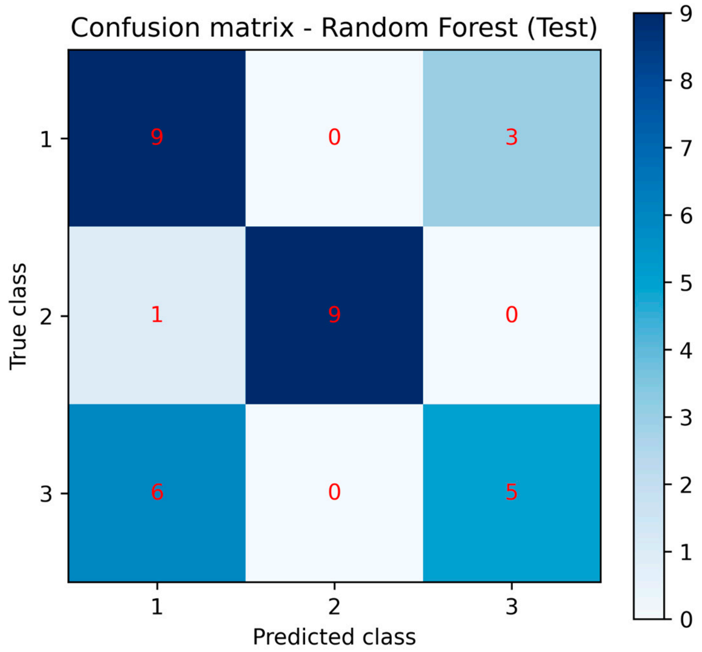

Figure S26. Confusion matrix of the classifier ensembles calculated on the test set for all sensory attributes of the odour

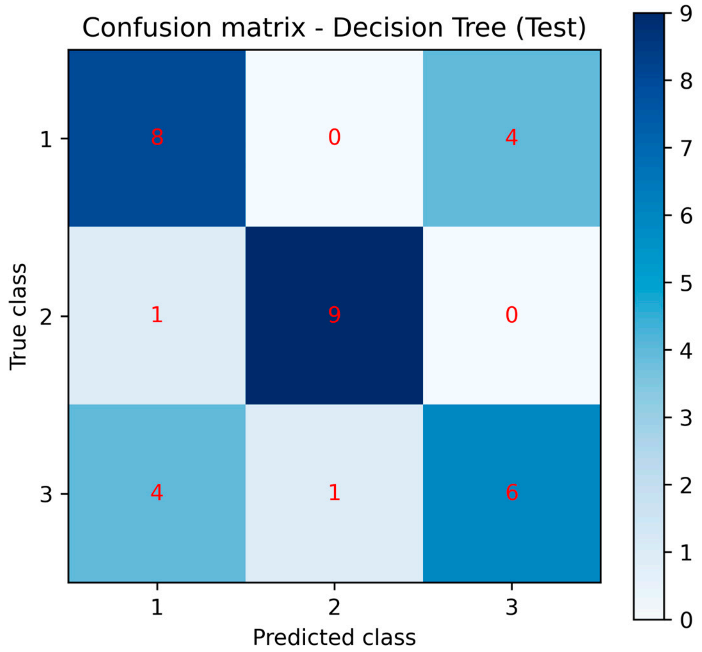

Figure S27. Confusion matrix of the classifier ensembles calculated on the test set for all sensory attributes of the odour

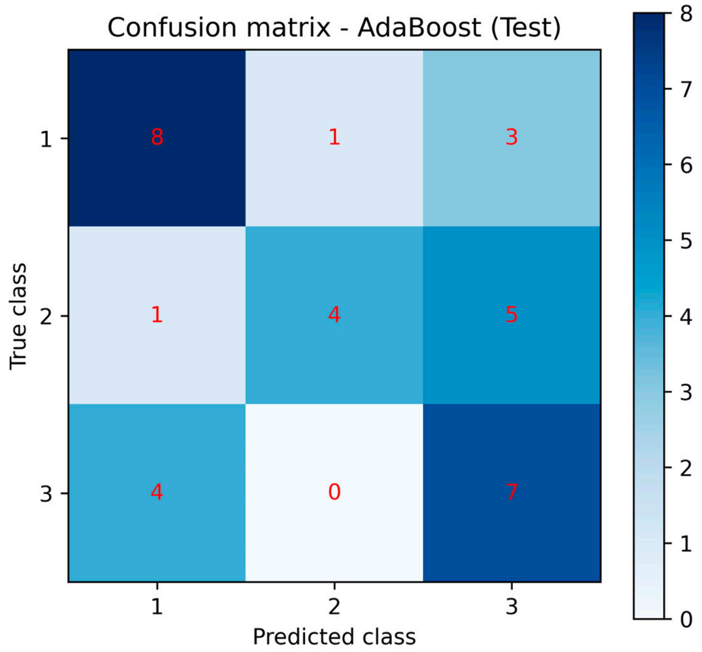

Figure S28. Confusion matrix of the classifier ensembles calculated on the test set for all sensory attributes of the odour

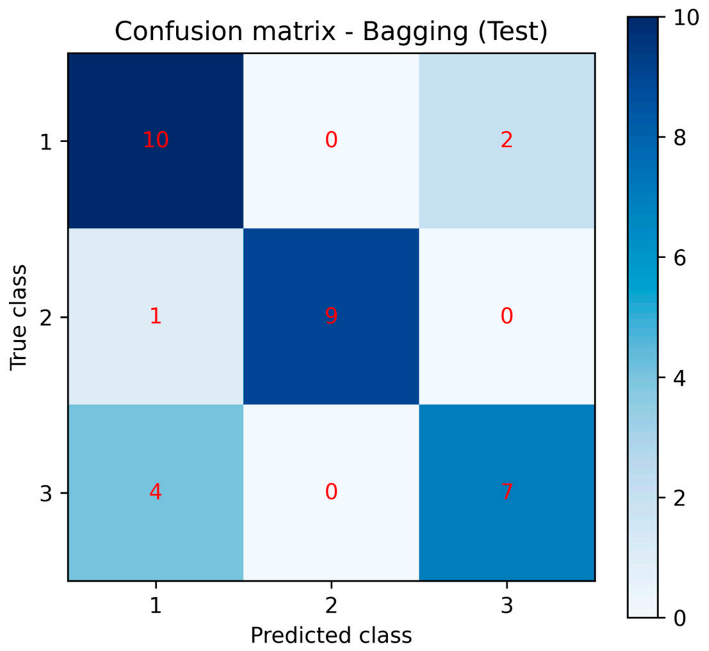

Figure S29. Confusion matrix of the classifier ensembles calculated on the test set for all sensory attributes of the odour

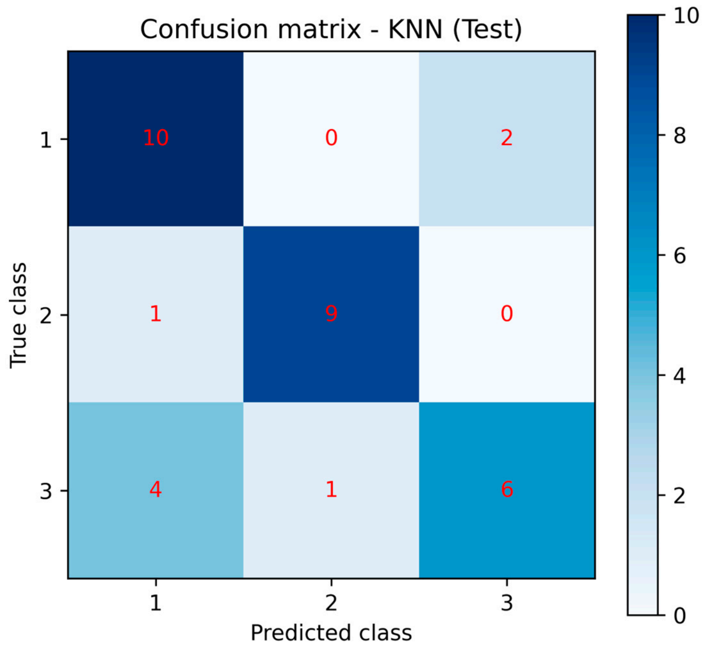

Figure S30. Confusion matrix of the classifier ensembles calculated on the test set for all sensory attributes of the odour

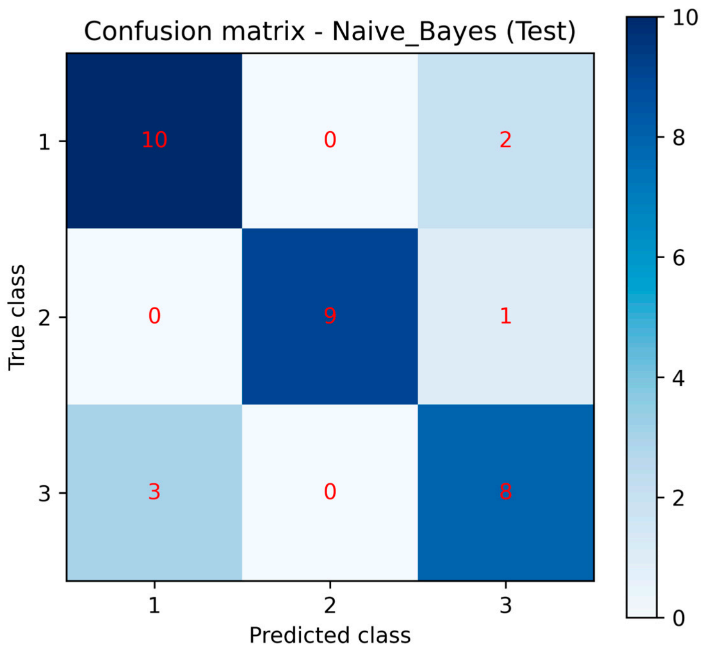

Figure S31. Confusion matrix of the classifier ensembles calculated on the test set for all sensory attributes of the odour

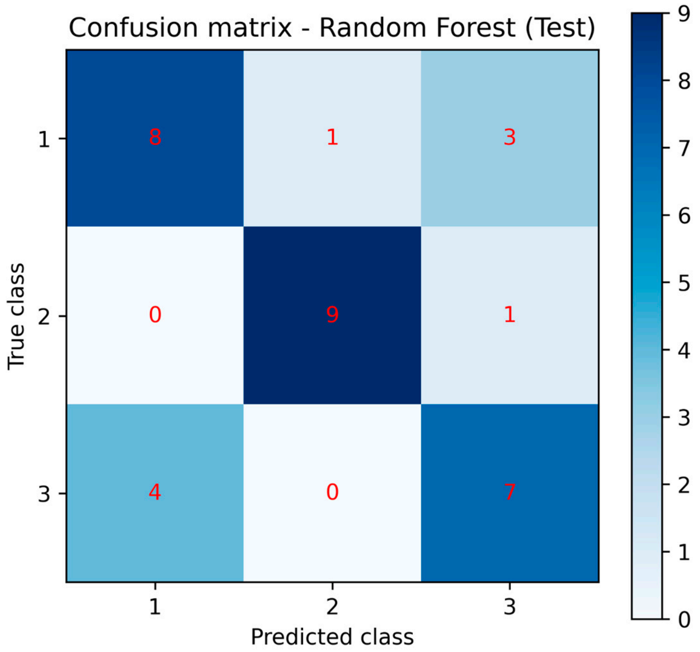

Figure S32. Confusion matrix of the classifier ensembles calculated on the test set for all sensory attributes of the odour

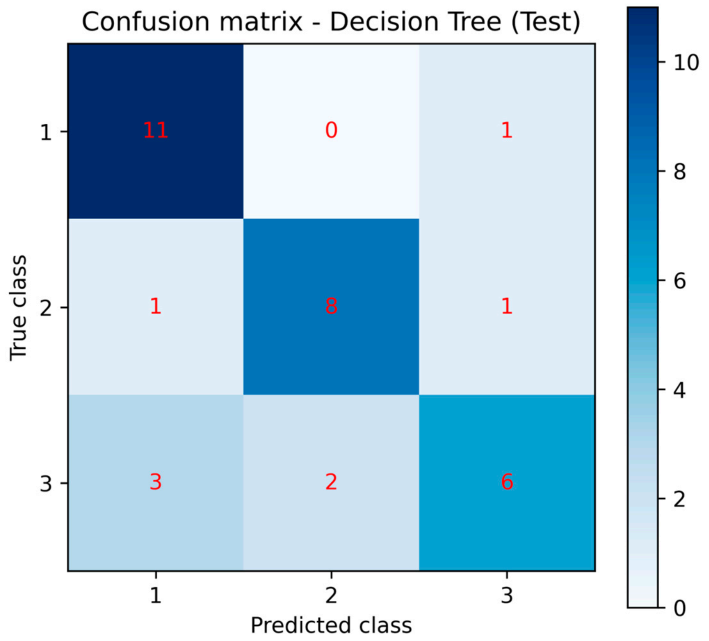

Figure S33. Confusion matrix of the classifier ensembles calculated on the test set for all sensory attributes of the odour

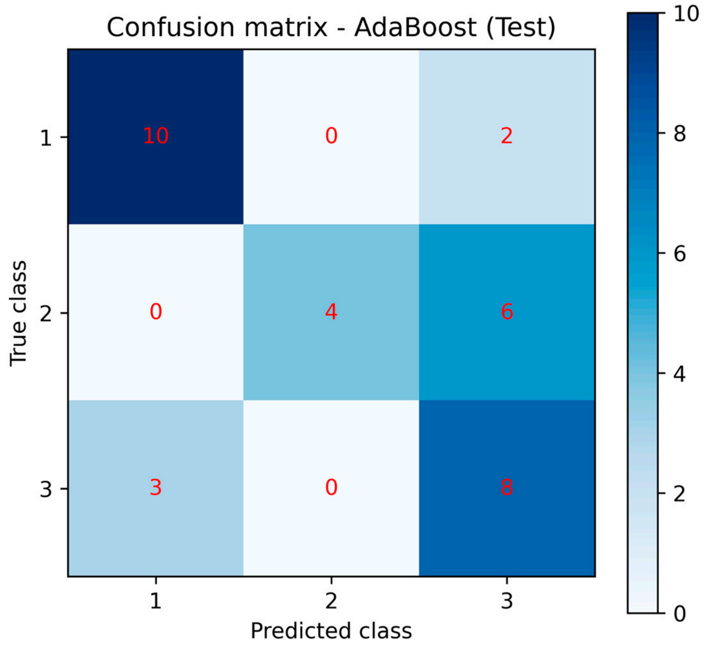

Figure S34. Confusion matrix of the classifier ensembles calculated on the test set for all sensory attributes of the odour

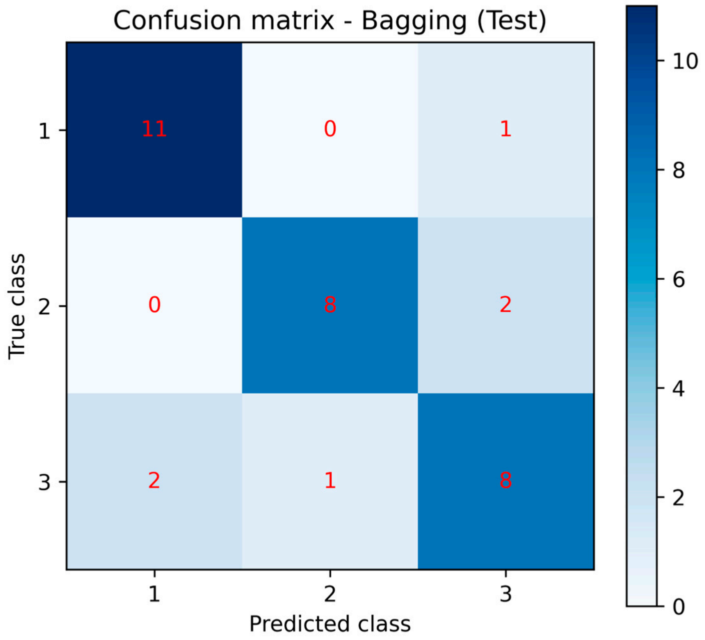

Figure S35. Confusion matrix of the classifier ensembles calculated on the test set for all sensory attributes of the odour

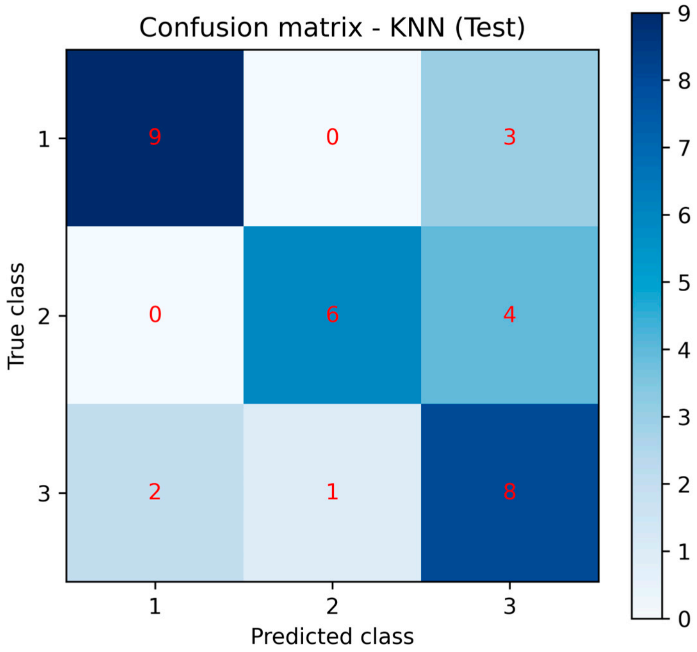

Figure S36. Confusion matrix of the classifier ensembles calculated on the test set for all sensory attributes of the odour

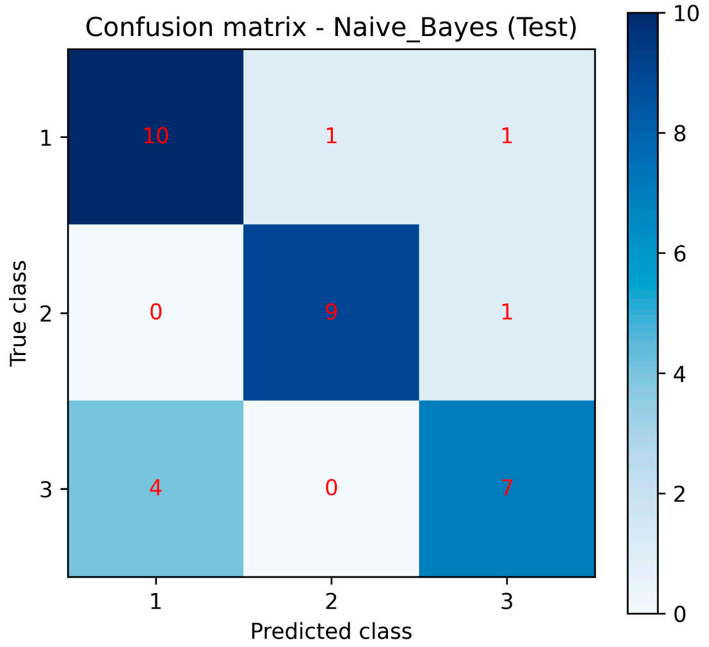

Figure S37. Confusion matrix of the classifier ensembles calculated on the test set for all sensory attributes of the odour

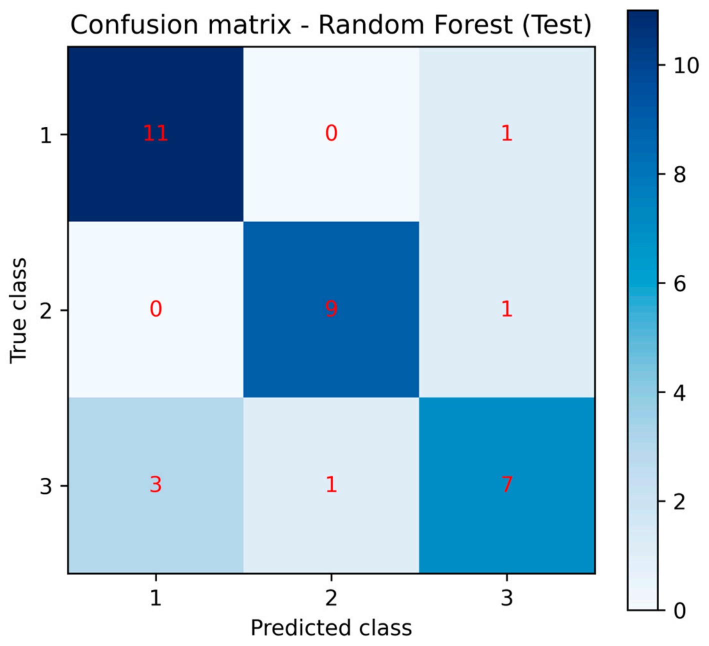

Figure S38. Confusion matrix of the classifier ensembles calculated on the test set for all sensory attributes of the odour

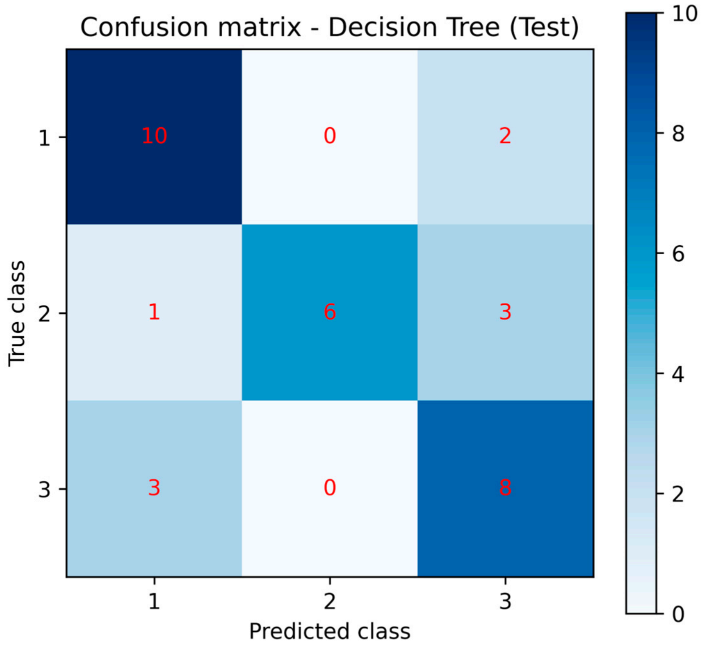

Figure S39. Confusion matrix of the classifier ensembles calculated on the test set for all sensory attributes of the odour

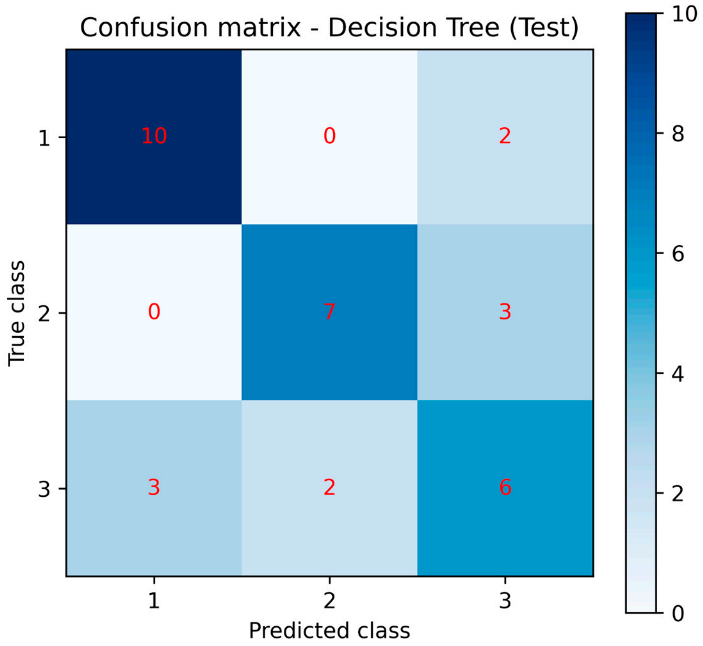

Figure S40. Confusion matrix of the classifier ensembles calculated on the test set for all sensory attributes of the odour

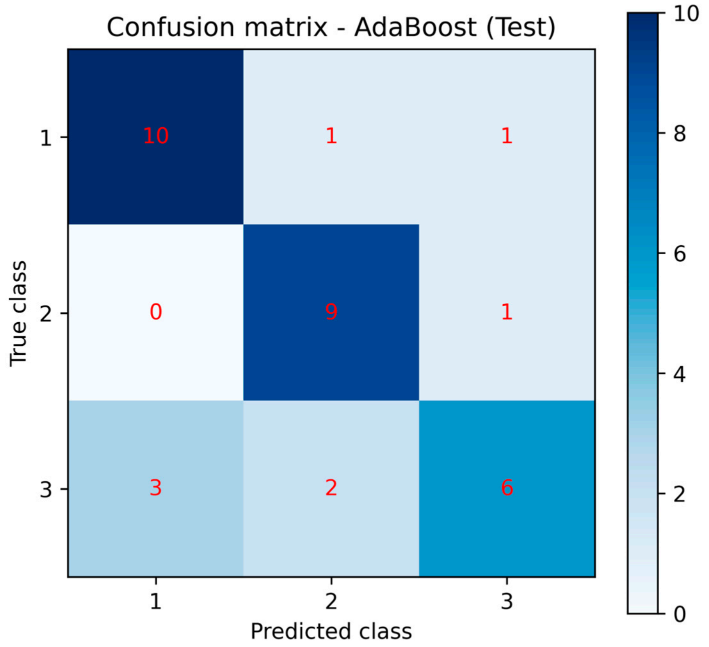

Figure S41. Confusion matrix of the classifier ensembles calculated on the test set for all sensory attributes of the odour

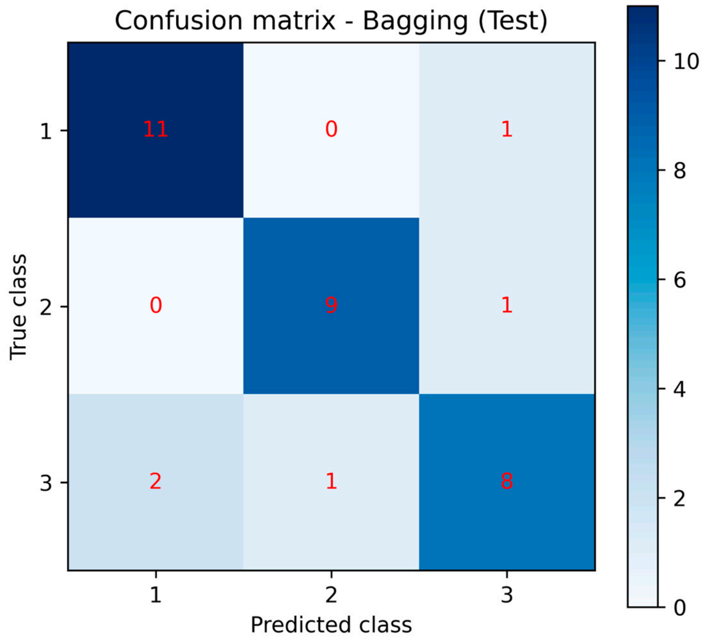

Figure S42. Confusion matrix of the classifier ensembles calculated on the test set for all sensory attributes of the odour

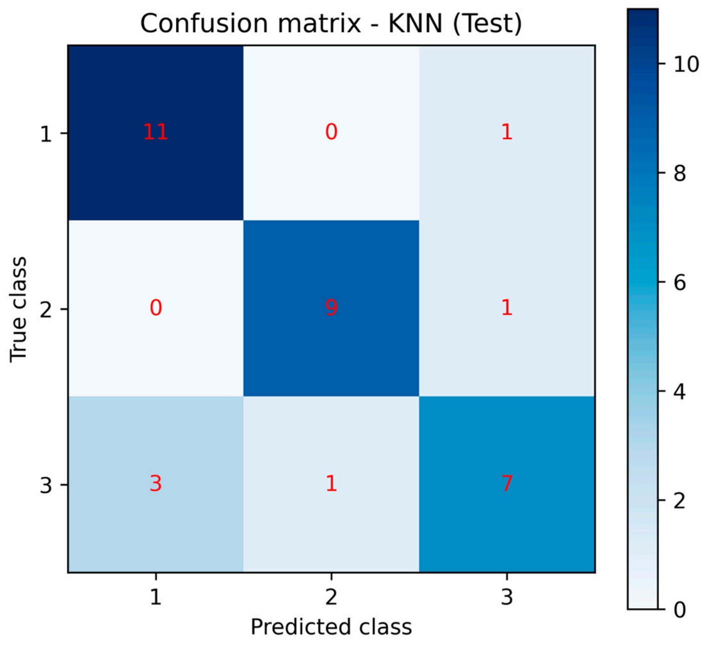

Figure S43. Confusion matrix of the classifier ensembles calculated on the test set for all sensory attributes of the odour

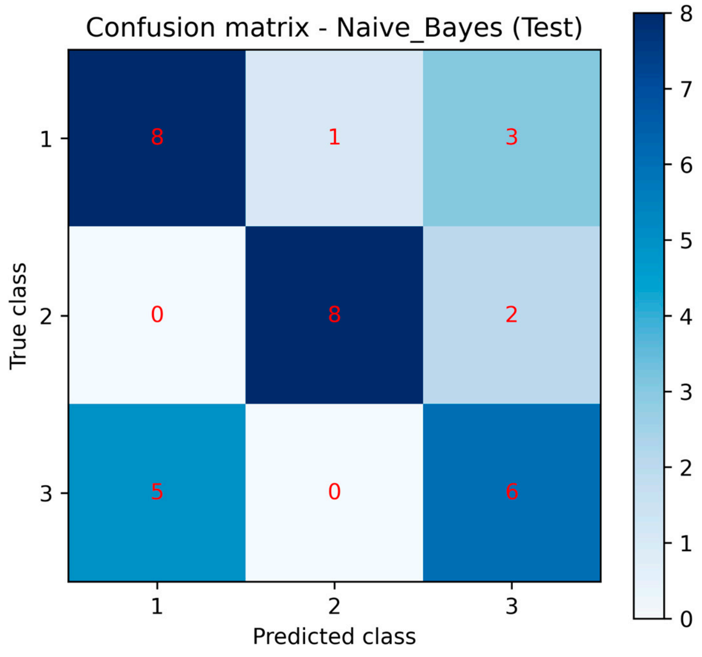

Figure S44. Confusion matrix of the classifier ensembles calculated on the test set for all sensory attributes of the odour

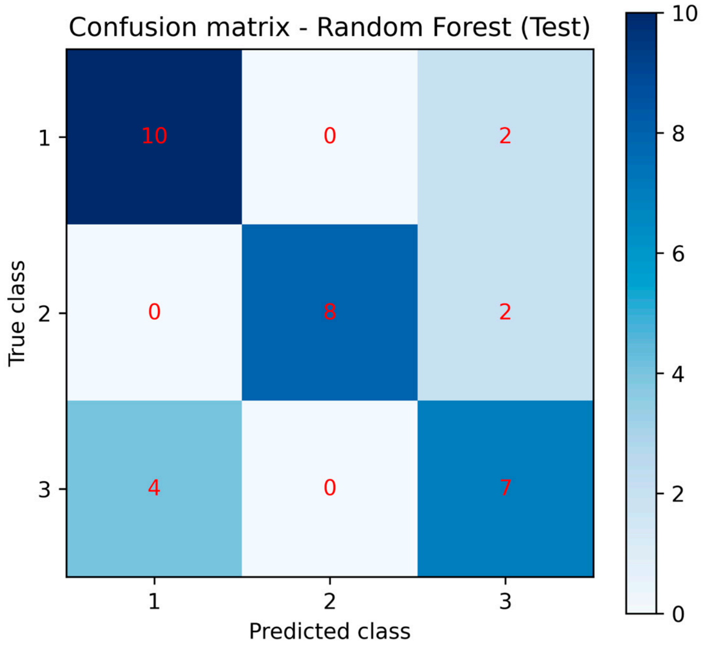

Figure S45. Confusion matrix of the classifier ensembles calculated on the test set for all sensory attributes of the odour

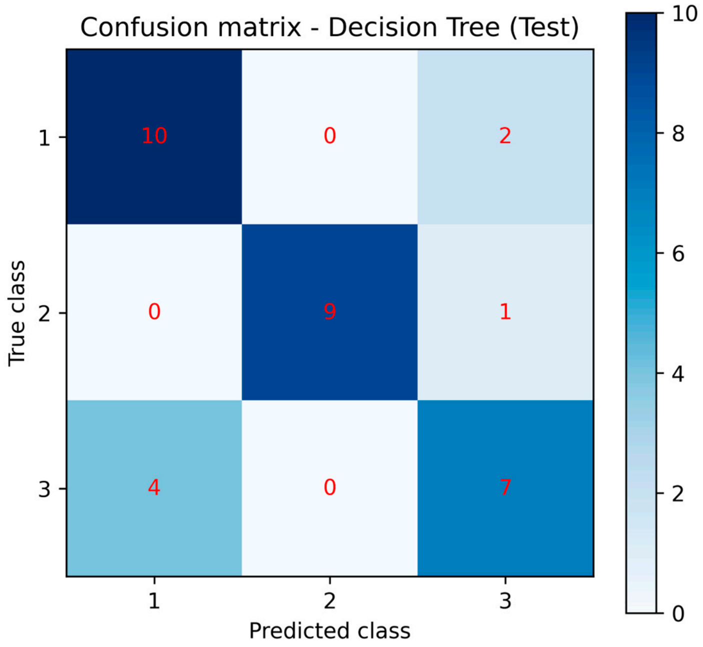

Figure S46. Confusion matrix of the classifier ensembles calculated on the test set for all sensory attributes of the odour

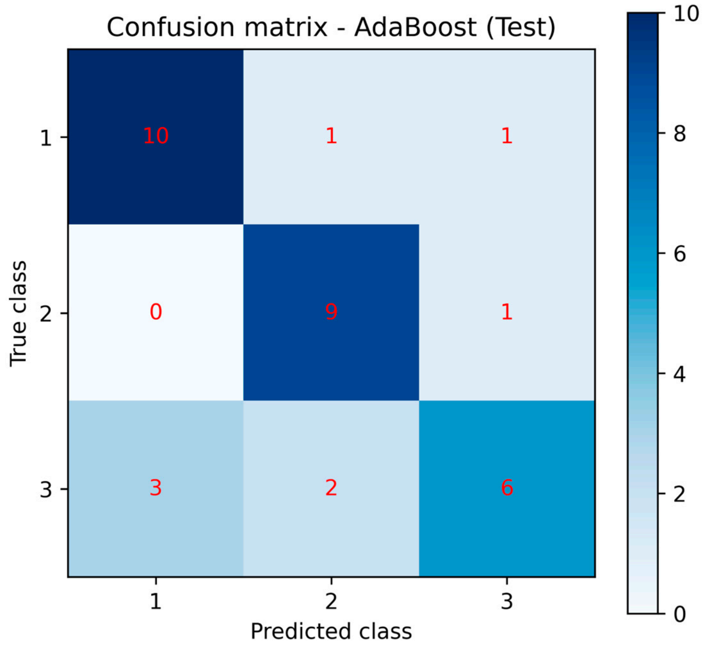

Figure S47. Confusion matrix of the classifier ensembles calculated on the test set for all sensory attributes of the odour

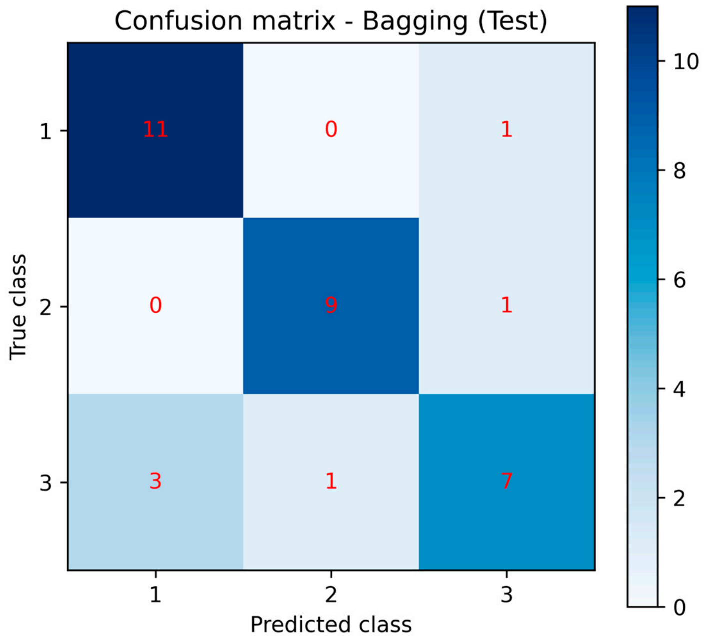

Figure S48. Confusion matrix of the classifier ensembles calculated on the test set for all sensory attributes of the odour

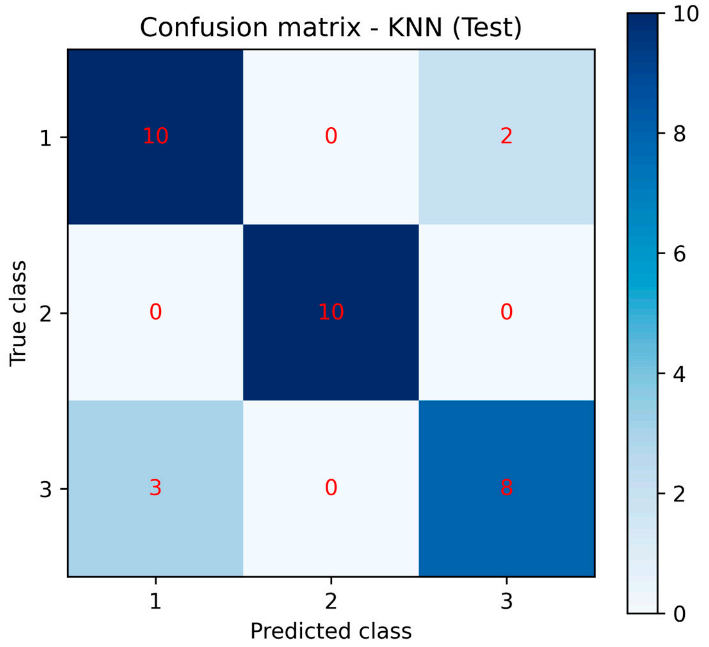

Figure S49. Confusion matrix of the classifier ensembles calculated on the test set for all sensory attributes of the odour

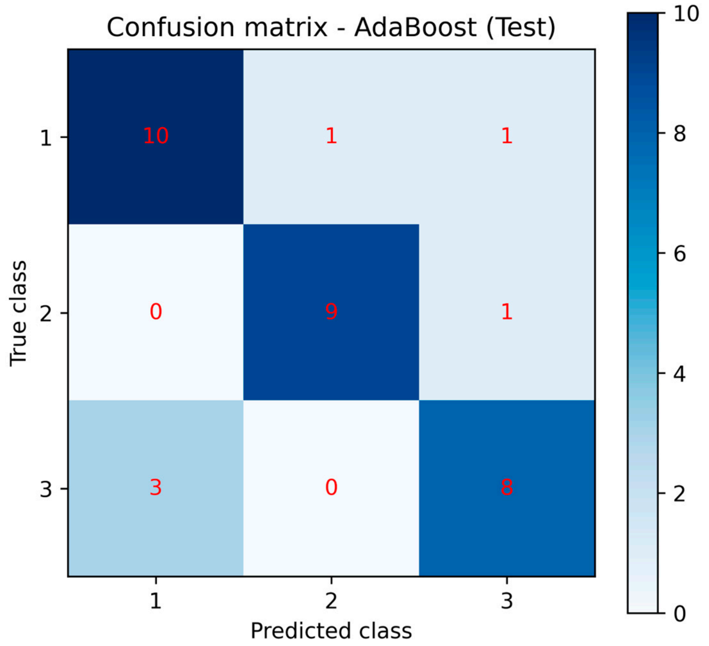

Figure S50. Confusion matrix of the classifier ensembles calculated on the test set for all sensory attributes of the odour

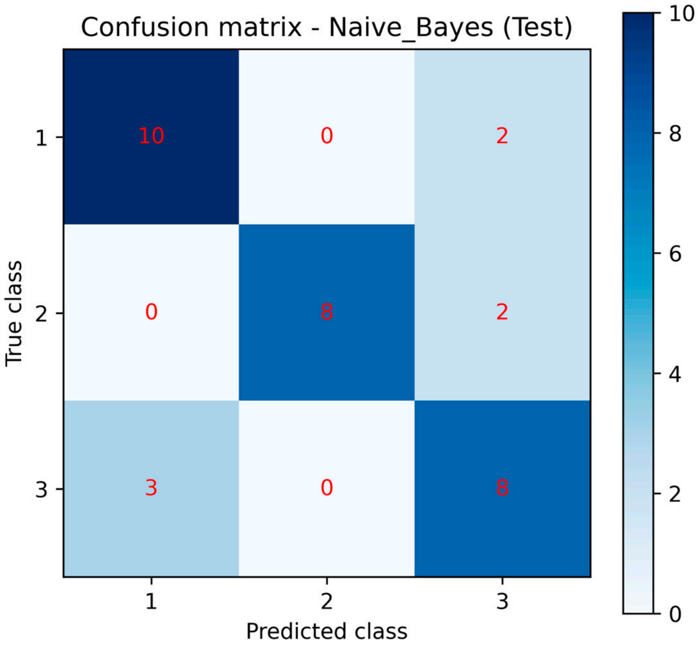

Figure S51. Confusion matrix of the classifier ensembles calculated on the test set for all sensory attributes of the odour

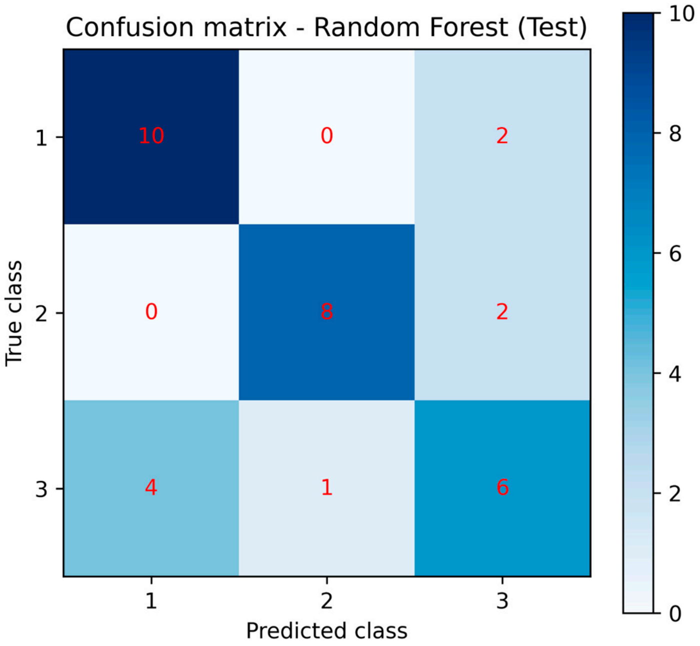

Figure S52. Confusion matrix of the classifier ensembles calculated on the test set for all sensory attributes of the odour

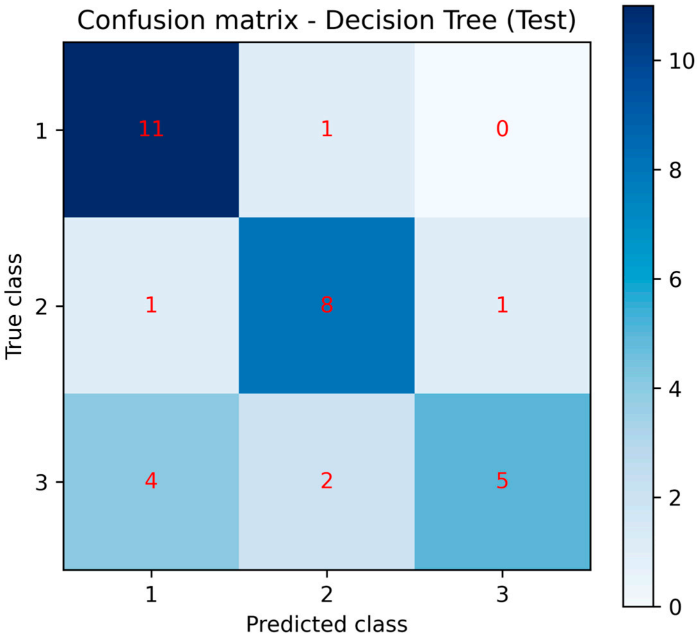

Figure S53. Confusion matrix of the classifier ensembles calculated on the test set for all sensory attributes of the odour

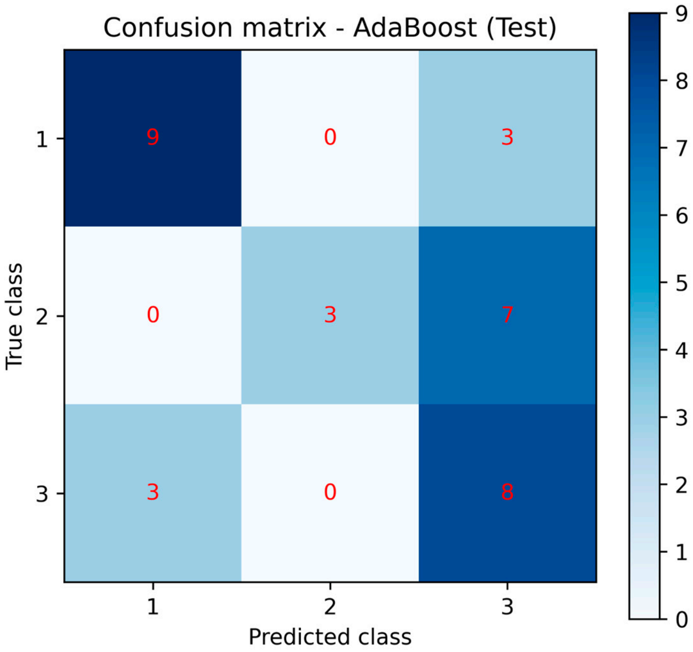

Figure S54. Confusion matrix of the classifier ensembles calculated on the test set for all sensory attributes of the odour

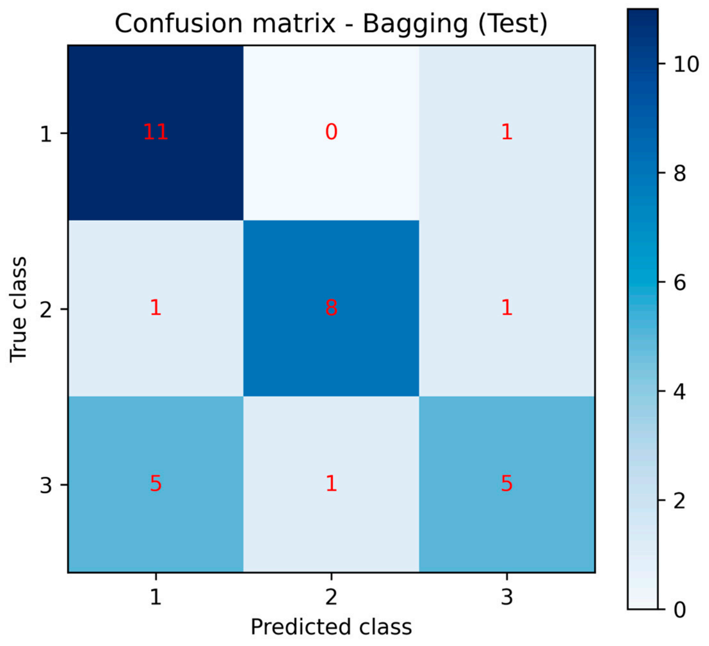

Figure S55. Confusion matrix of the classifier ensembles calculated on the test set for all sensory attributes of the odour

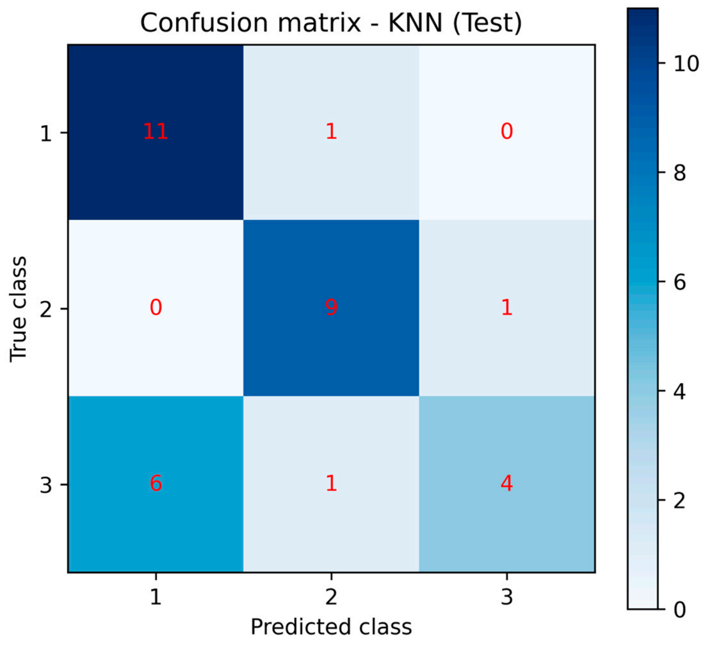

Figure S56. Confusion matrix of the classifier ensembles calculated on the test set for all sensory attributes of the odour

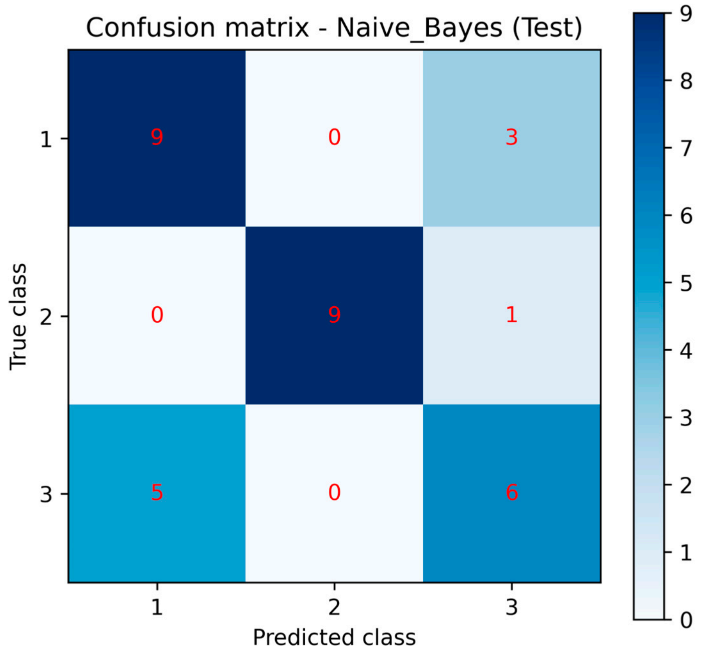

Figure S57. Confusion matrix of the classifier ensembles calculated on the test set for all sensory attributes of the odour

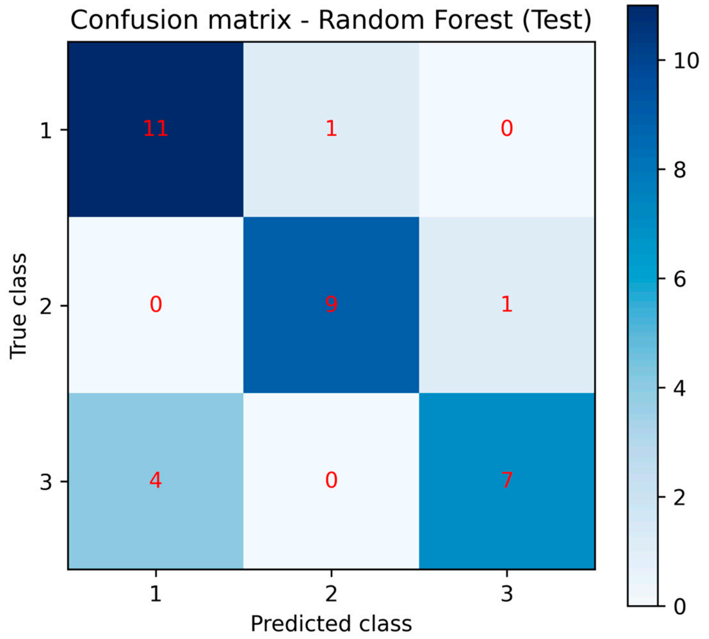

Figure S58. Confusion matrix of the classifier ensembles calculated on the test set for all sensory attributes of the odour

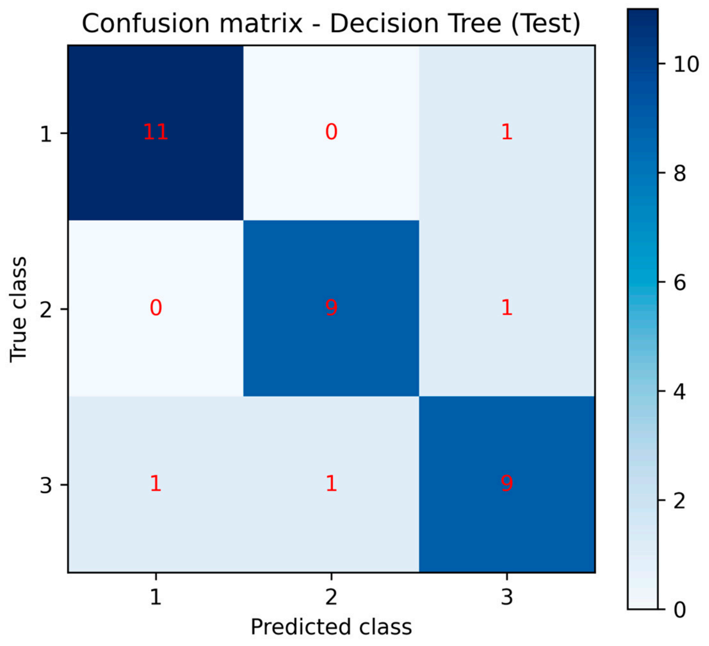

Figure S59. Confusion matrix of the classifier ensembles calculated on the test set for all sensory attributes of the odour

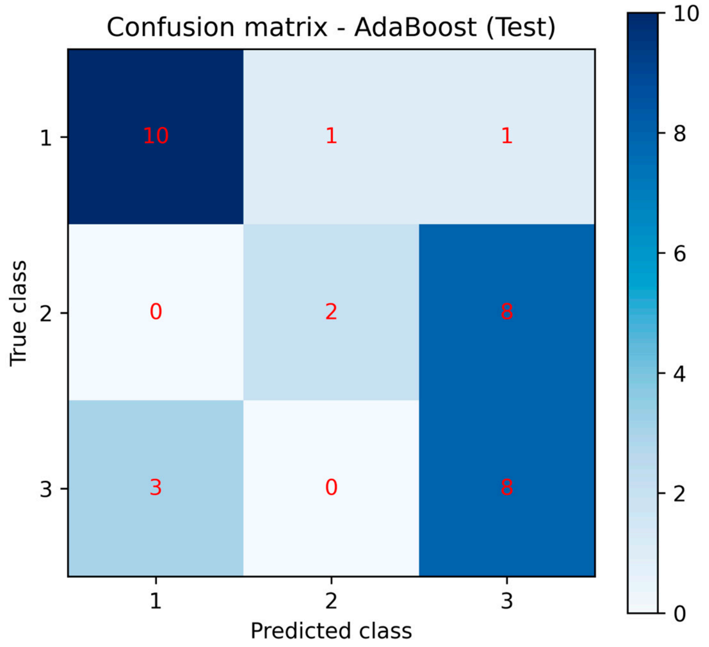

Figure S60. Confusion matrix of the classifier ensembles calculated on the test set for all sensory attributes of the odour

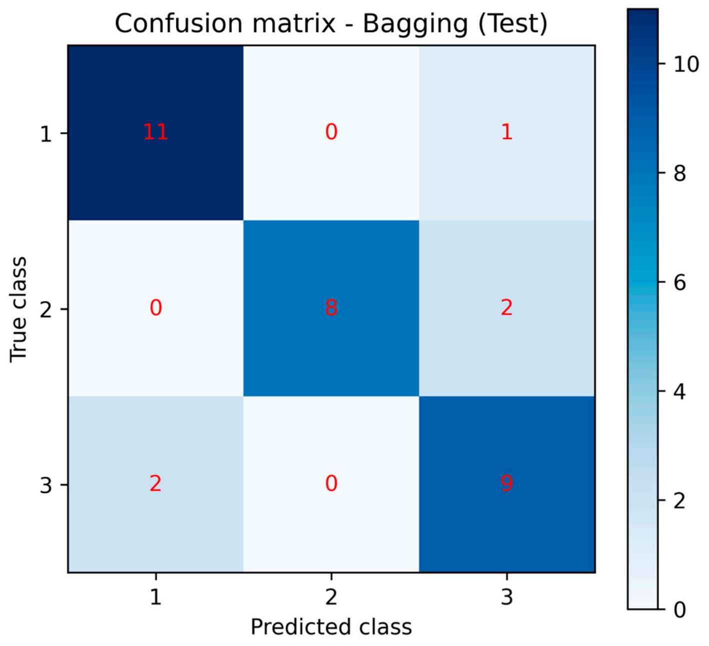

Figure S61. Confusion matrix of the classifier ensembles calculated on the test set for all sensory attributes of the odour

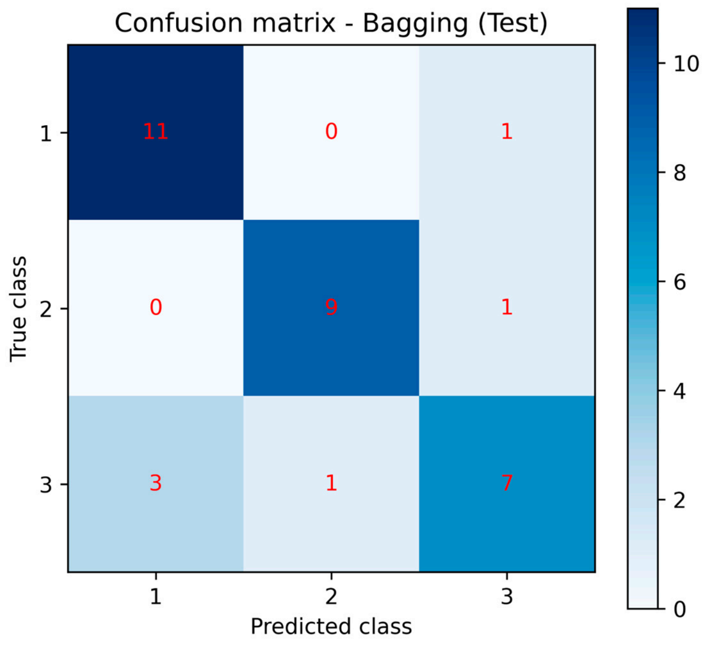

Figure S62. Confusion matrix of the classifier ensembles calculated on the test set for all sensory attributes of the odour

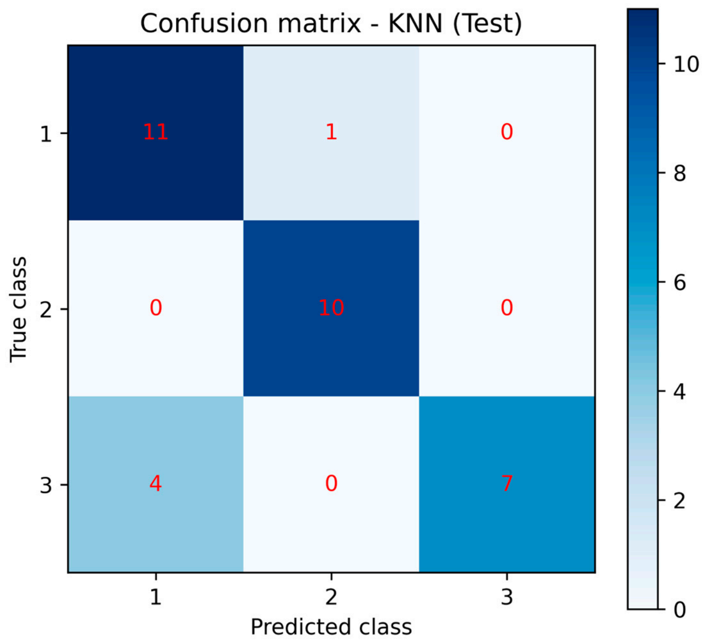

Figure S63. Confusion matrix of the classifier ensembles calculated on the test set for all sensory attributes of the odour

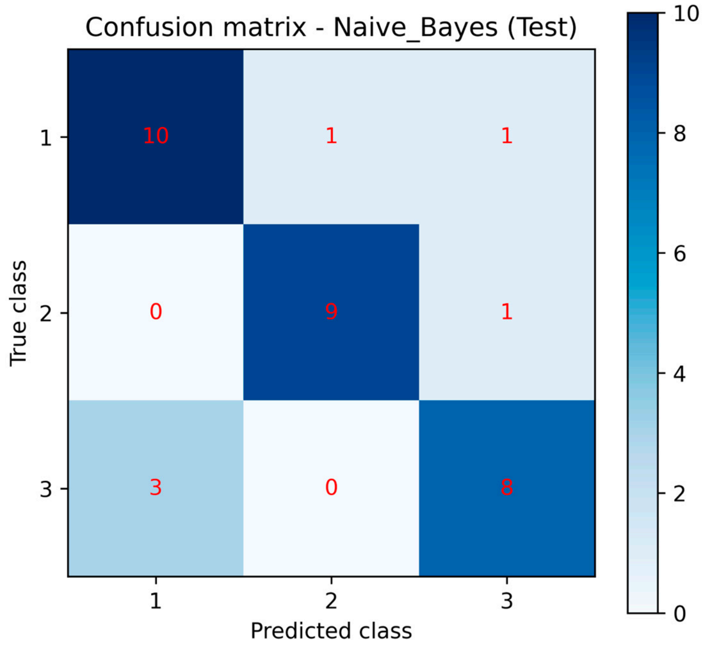

Figure S64. Confusion matrix of the classifier ensembles calculated on the test set for all sensory attributes of the odour

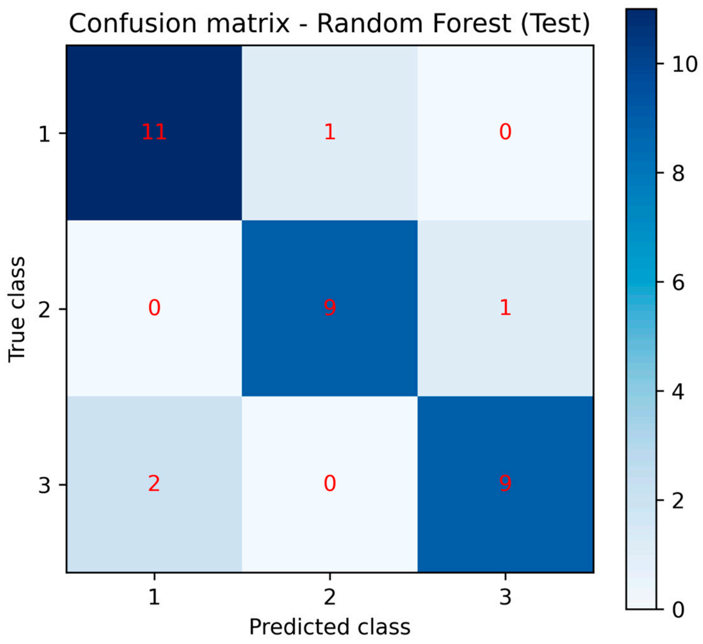

Figure S65. Confusion matrix of the classifier ensembles calculated on the test set for all sensory attributes of the odour

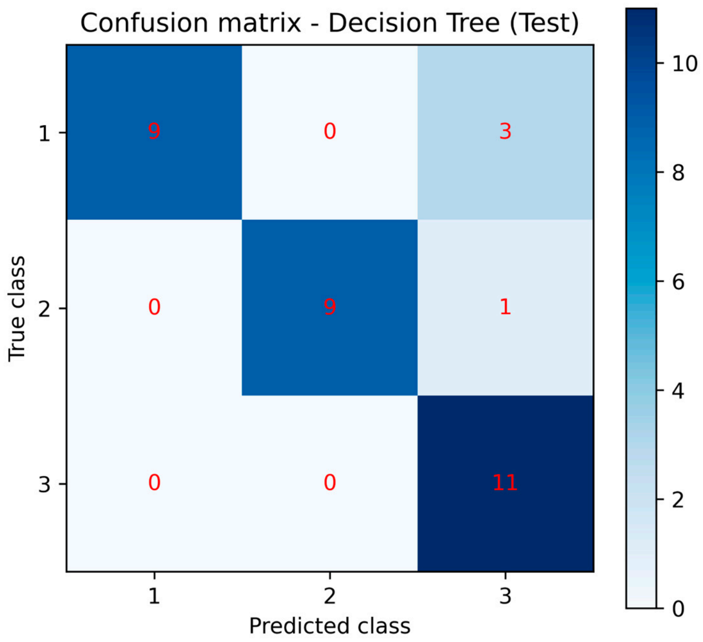

Figure S66. Confusion matrix of the classifier ensembles calculated on the test set for all sensory attributes of the odour

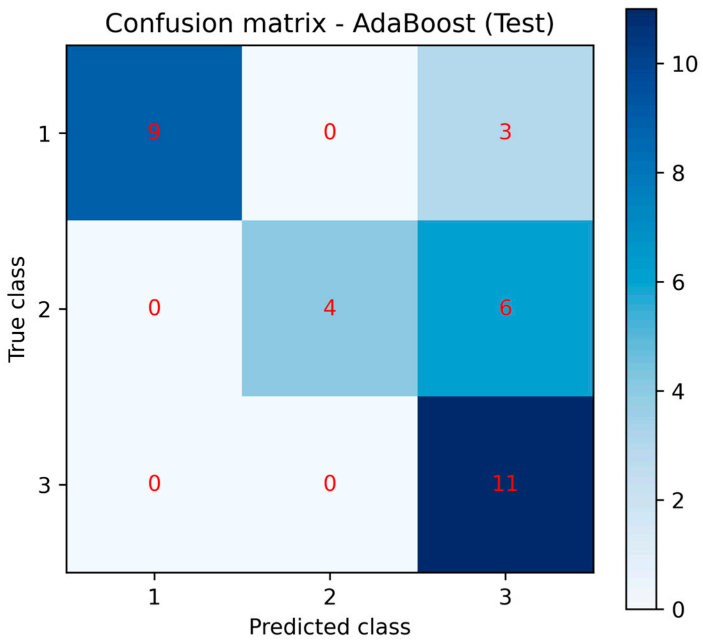

Figure S67. Confusion matrix of the classifier ensembles calculated on the test set for all sensory attributes of the odour

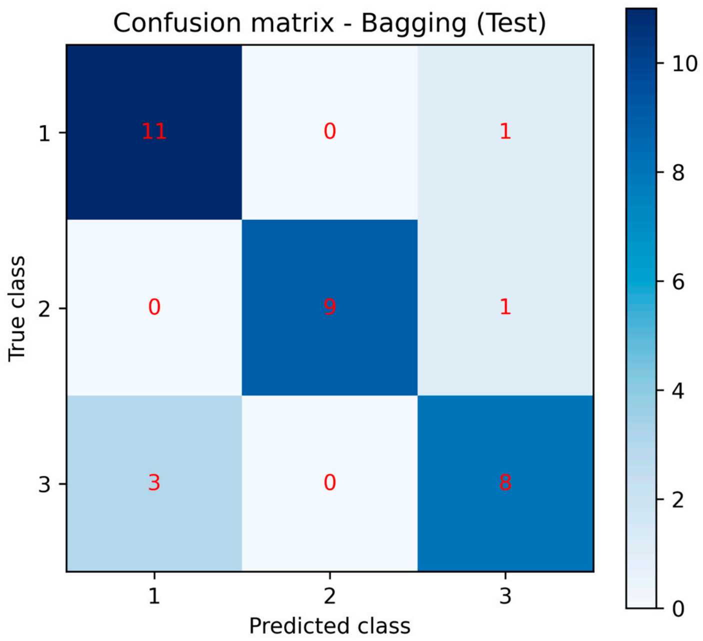

Figure S68. Confusion matrix of the classifier ensembles calculated on the test set for all sensory attributes of the odour

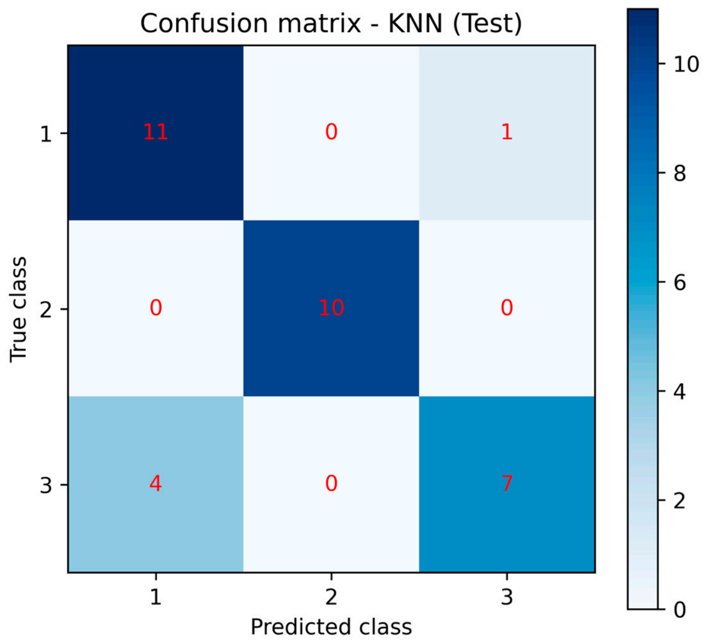

Figure S69. Confusion matrix of the classifier ensembles calculated on the test set for all sensory attributes of the odour

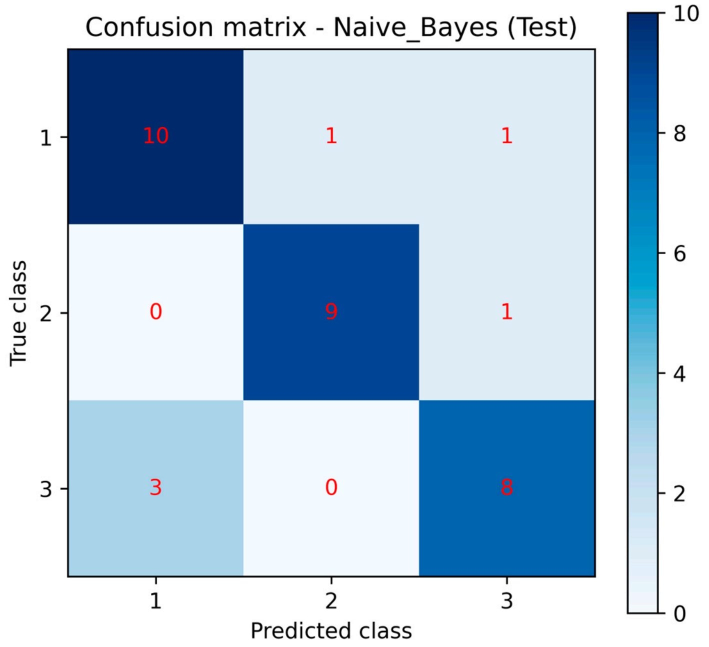

Figure S70. Confusion matrix of the classifier ensembles calculated on the test set for all sensory attributes of the odour

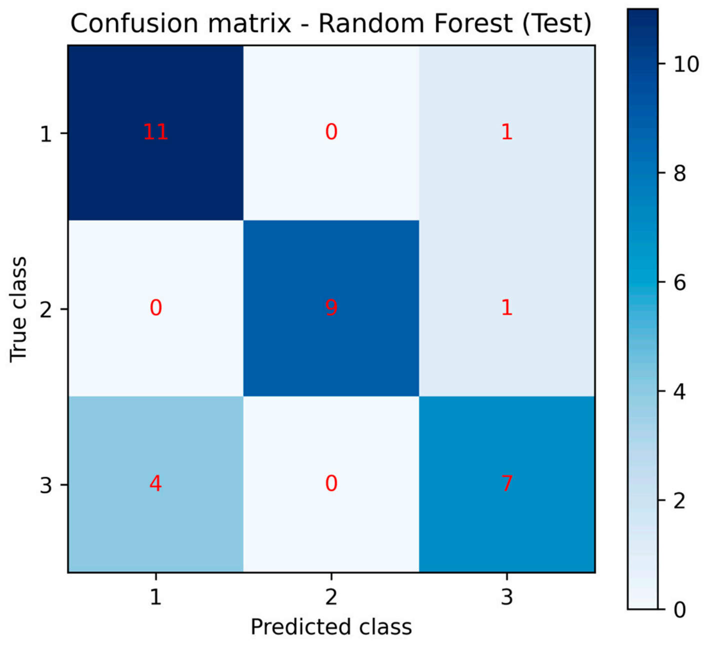

Figure S71. Confusion matrix of the classifier ensembles calculated on the test set for all sensory attributes of the odour

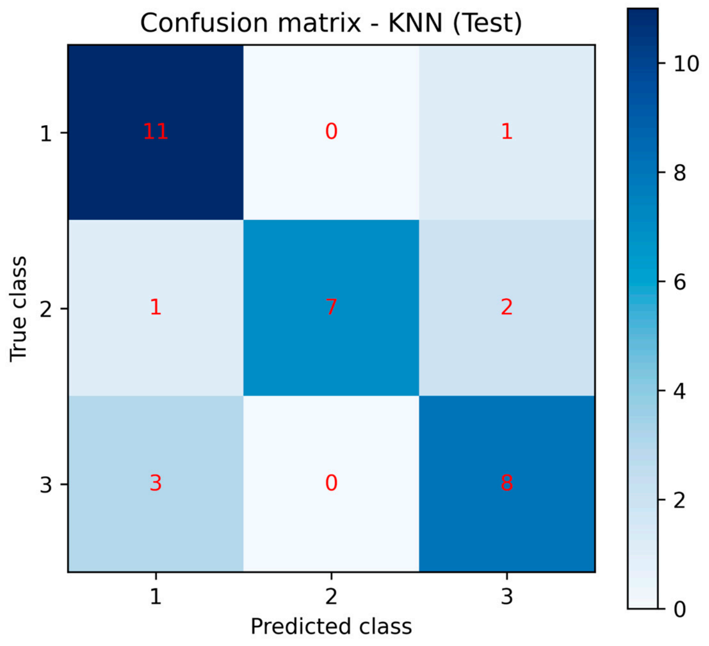

Figure S72. Confusion matrix of the classifier ensembles calculated on the test set for all sensory attributes of the odour

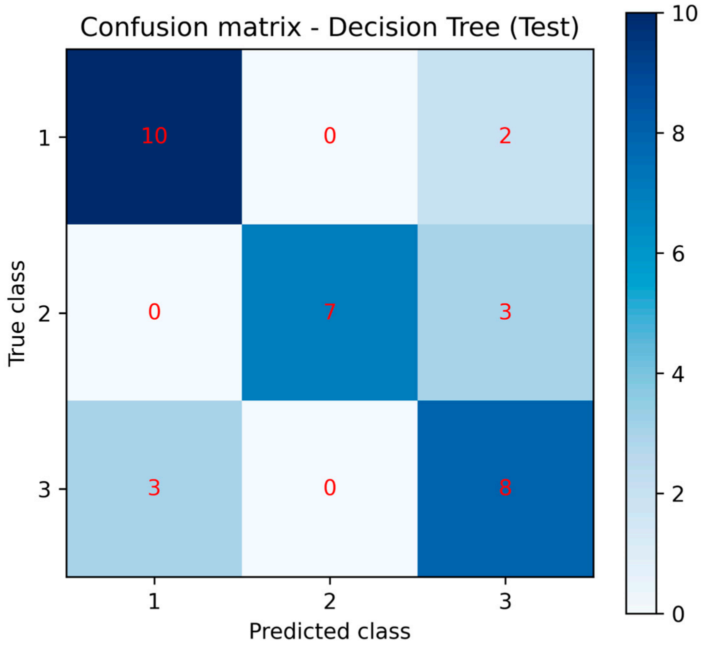

Figure S73. Confusion matrix of the classifier ensembles calculated on the test set for all sensory attributes of the odour

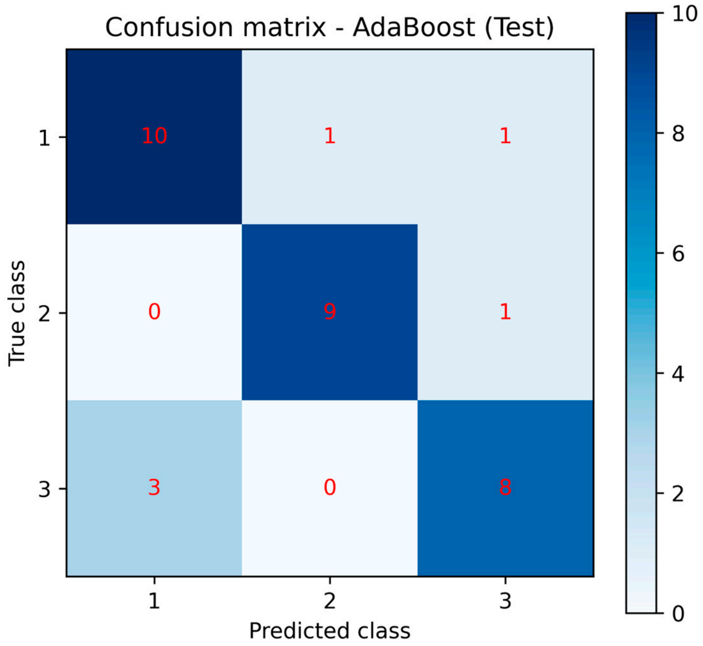

Figure S74. Confusion matrix of the classifier ensembles calculated on the test set for all sensory attributes of the odour

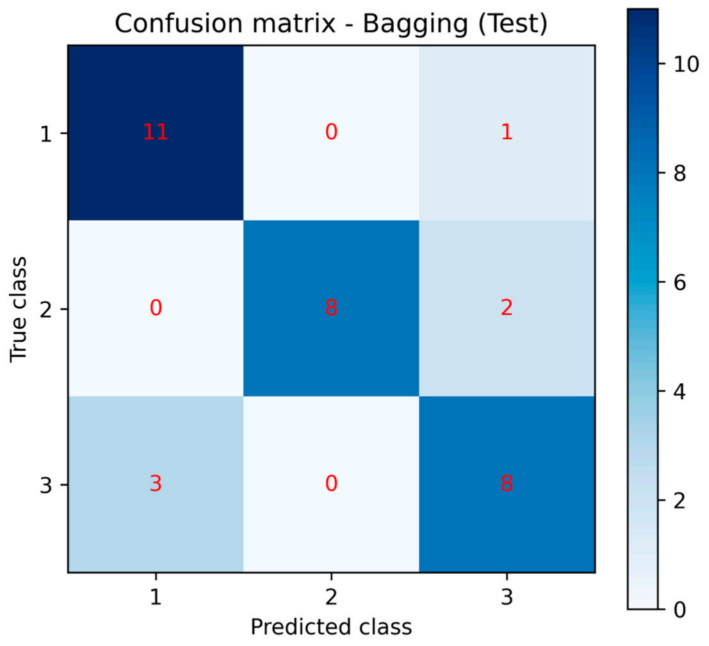

Figure S75. Confusion matrix of the classifier ensembles calculated on the test set for all sensory attributes of the odour

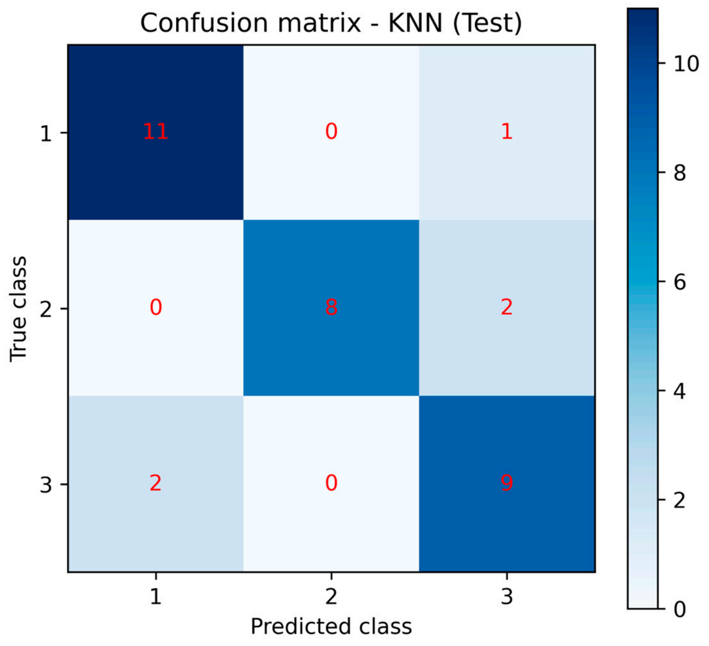

Figure S76. Confusion matrix of the classifier ensembles calculated on the test set for all sensory attributes of the odour

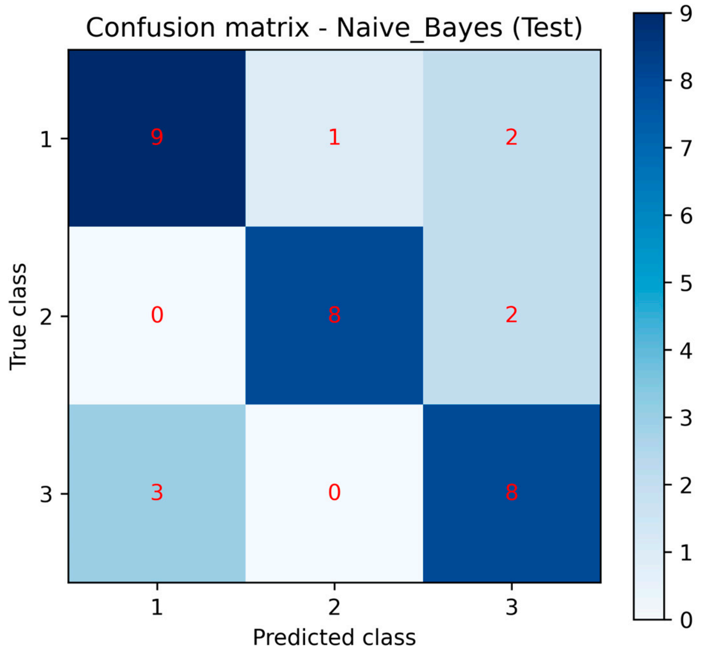

Figure S77. Confusion matrix of the classifier ensembles calculated on the test set for all sensory attributes of the odour

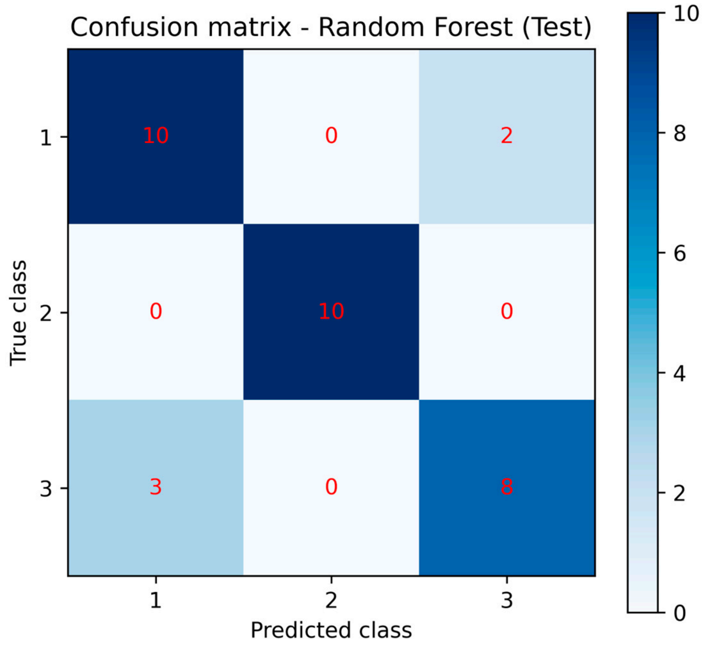

Figure S78. Confusion matrix of the classifier ensembles calculated on the test set for all sensory attributes of the odour

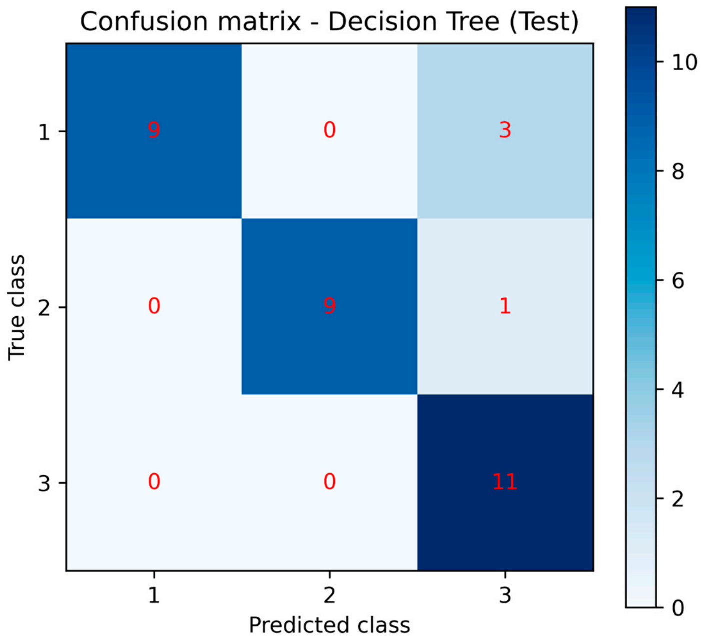

Figure S79. Confusion matrix of the classifier ensembles calculated on the test set for all sensory attributes of the odour

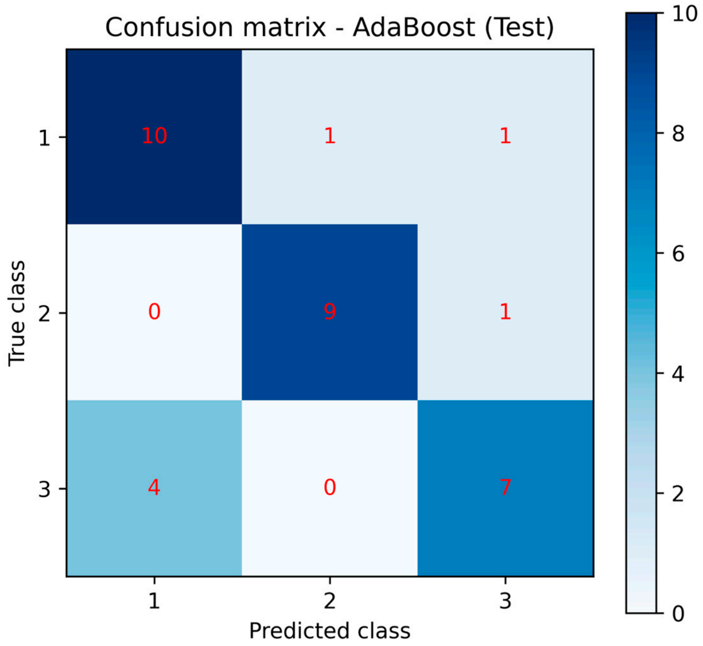

Figure S80. Confusion matrix of the classifier ensembles calculated on the test set for all sensory attributes of the odour

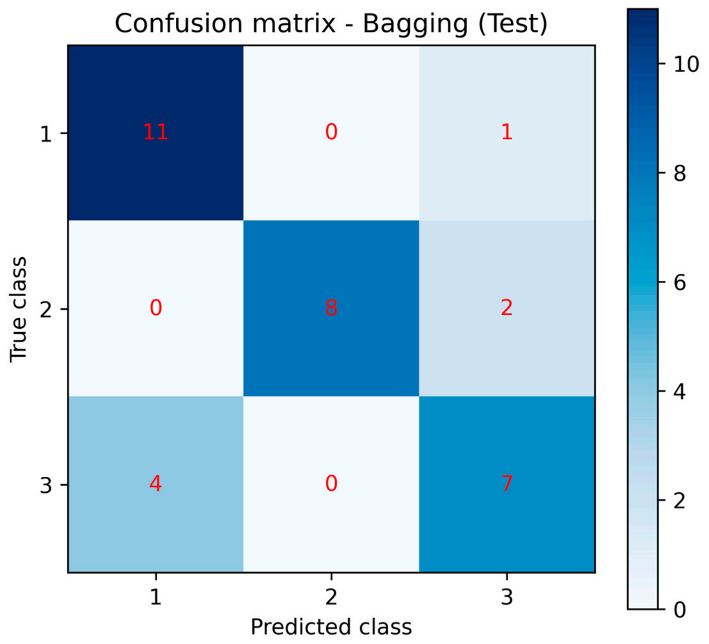

Figure S81. Confusion matrix of the classifier ensembles calculated on the test set for all sensory attributes of the odour

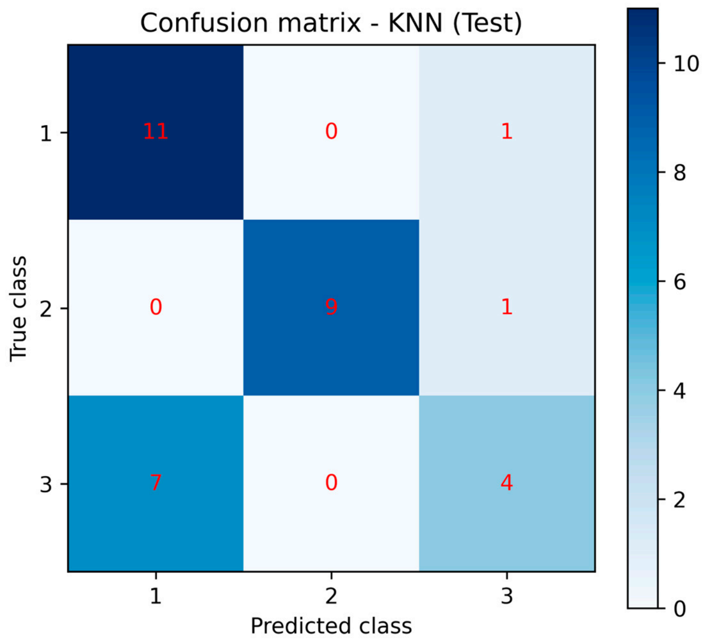

Figure S82. Confusion matrix of the classifier ensembles calculated on the test set for all sensory attributes of the odour

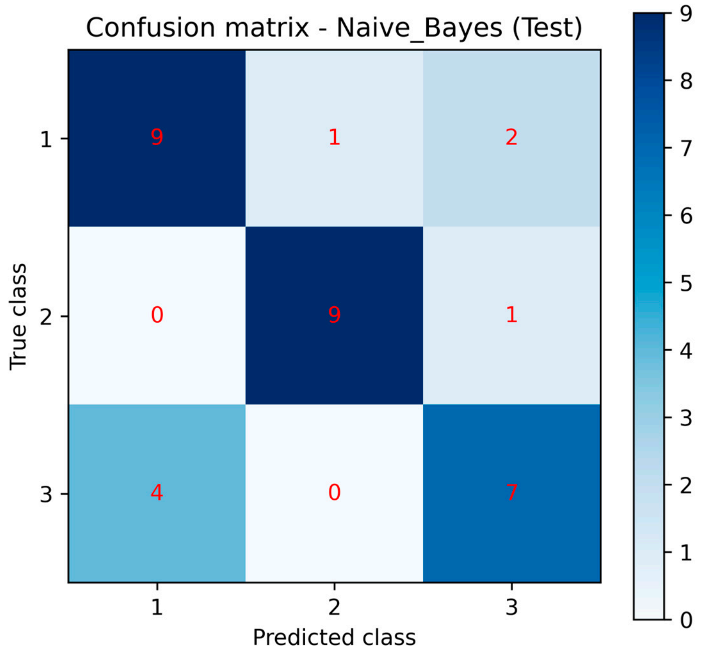

Figure S83. Confusion matrix of the classifier ensembles calculated on the test set for all sensory attributes of the odour

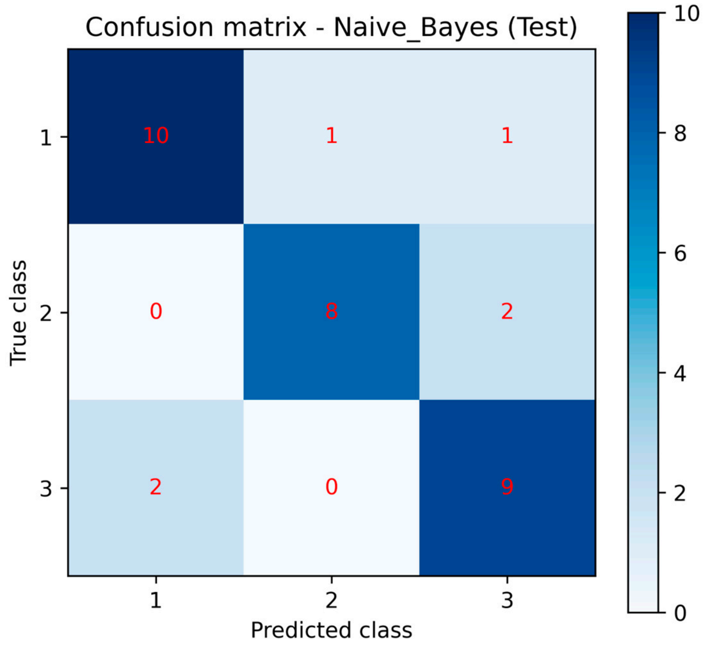

Figure S84. Confusion matrix of the classifier ensembles calculated on the test set for all sensory attributes of the odour

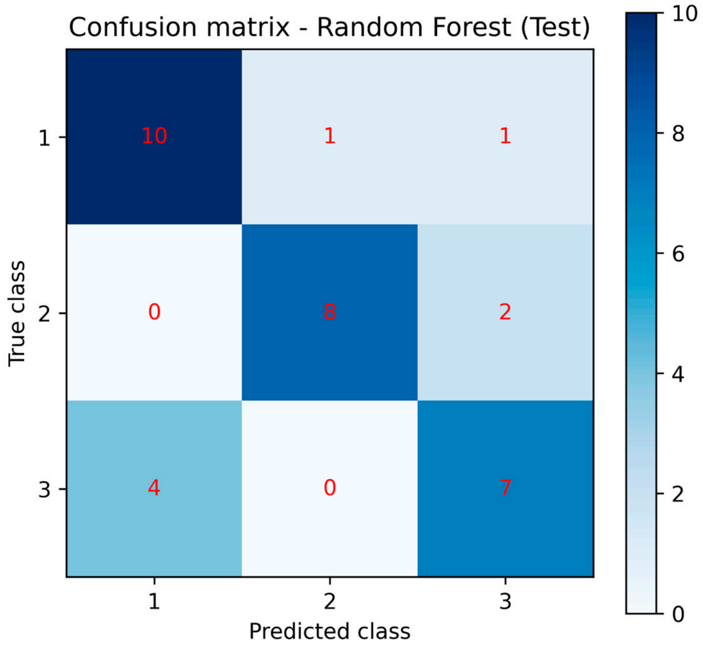

Figure S85. Confusion matrix of the classifier ensembles calculated on the test set for all sensory attributes of the odour

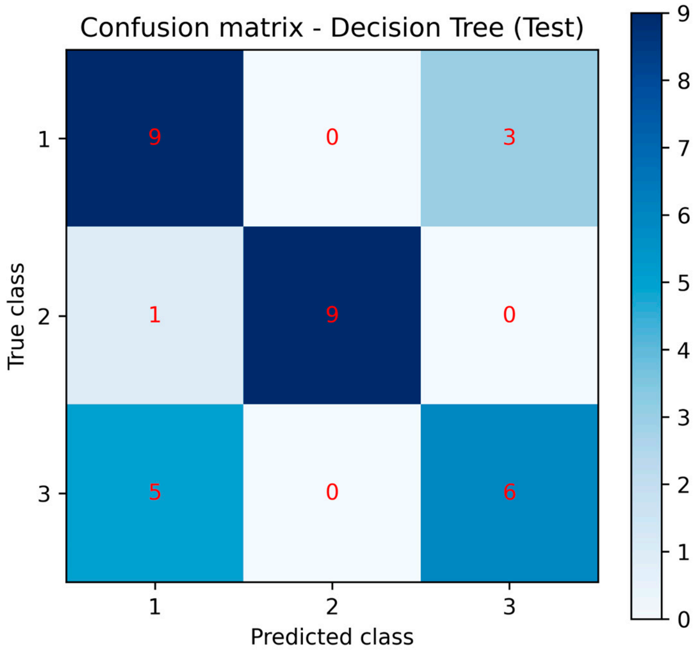

Figure S86. Confusion matrix of the classifier ensembles calculated on the test set for all sensory attributes of the odour

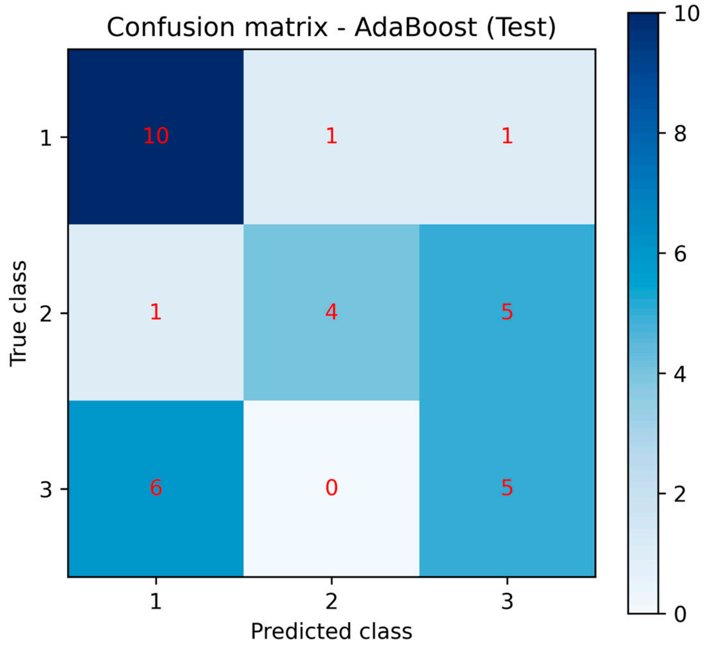

Figure S87. Confusion matrix of the classifier ensembles calculated on the test set for all sensory attributes of the odour

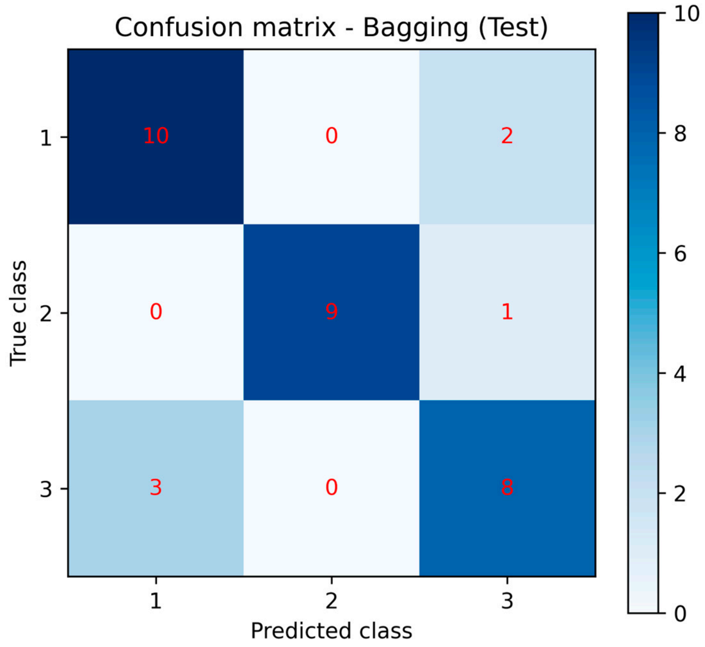

Figure S88. Confusion matrix of the classifier ensembles calculated on the test set for all sensory attributes of the odour

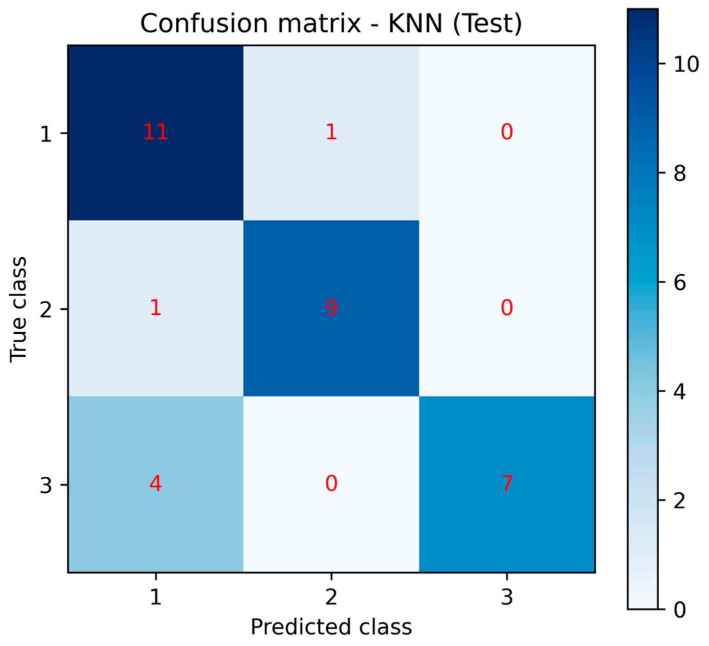

Figure S89. Confusion matrix of the classifier ensembles calculated on the test set for all sensory attributes of the odour

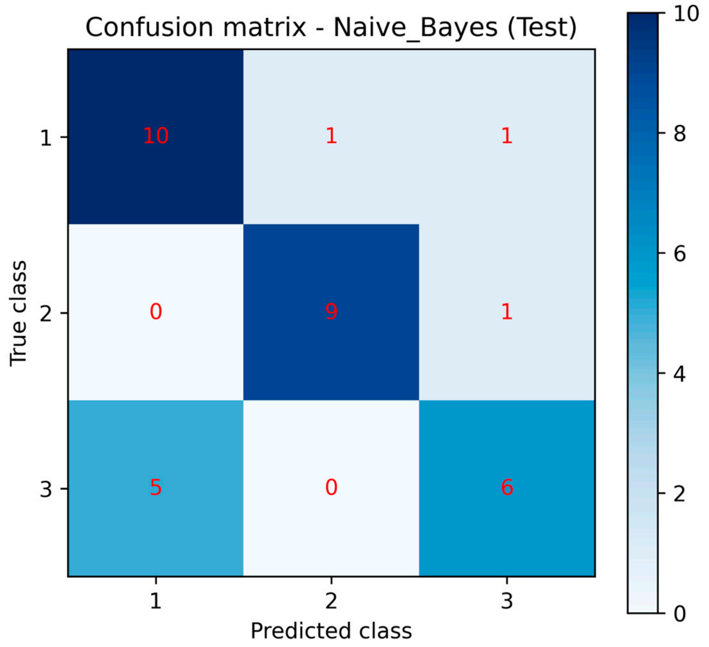

Figure S90. Confusion matrix of the classifier ensembles calculated on the test set for all sensory attributes of the odour

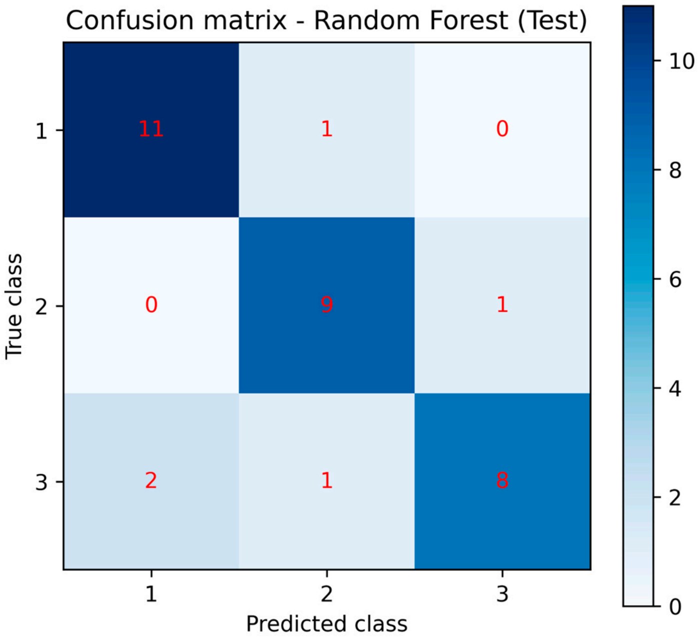

Figure S91. Confusion matrix of the classifier ensembles calculated on the test set for all sensory attributes of the odour

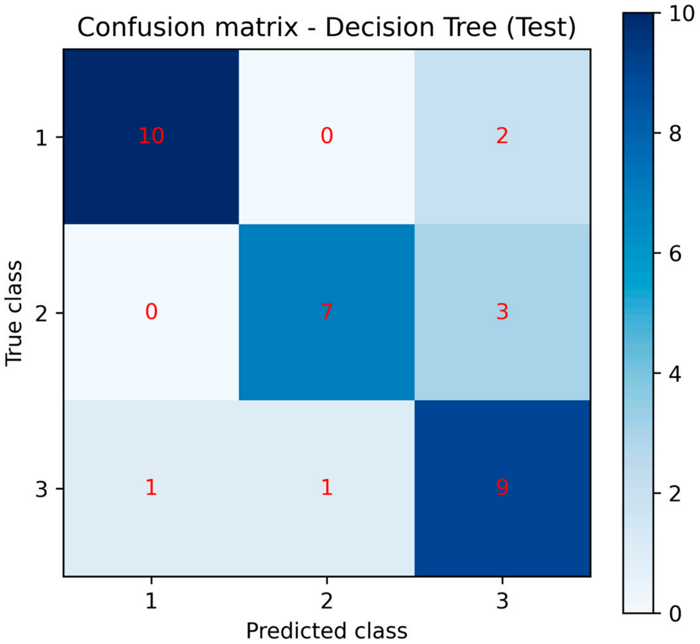

Figure S92. Confusion matrix of the classifier ensembles calculated on the test set for all sensory attributes of the odour

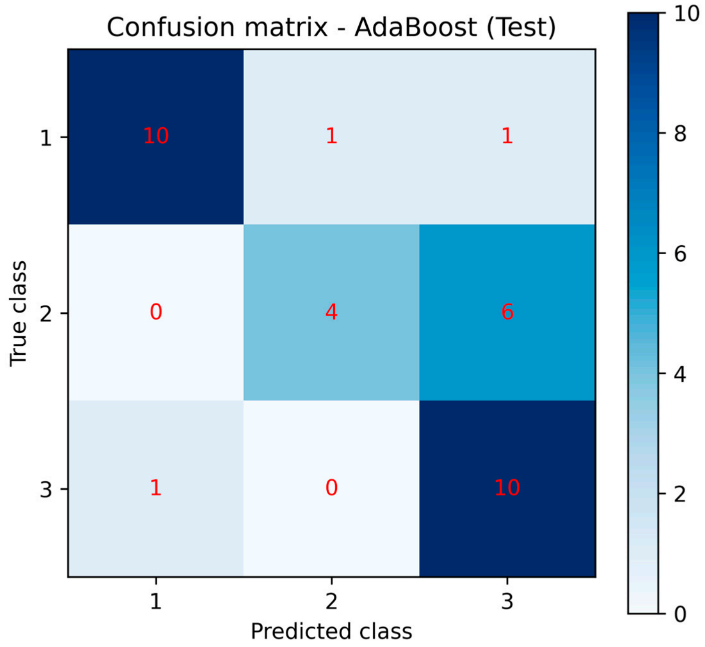

Figure S93. Confusion matrix of the classifier ensembles calculated on the test set for all sensory attributes of the odour

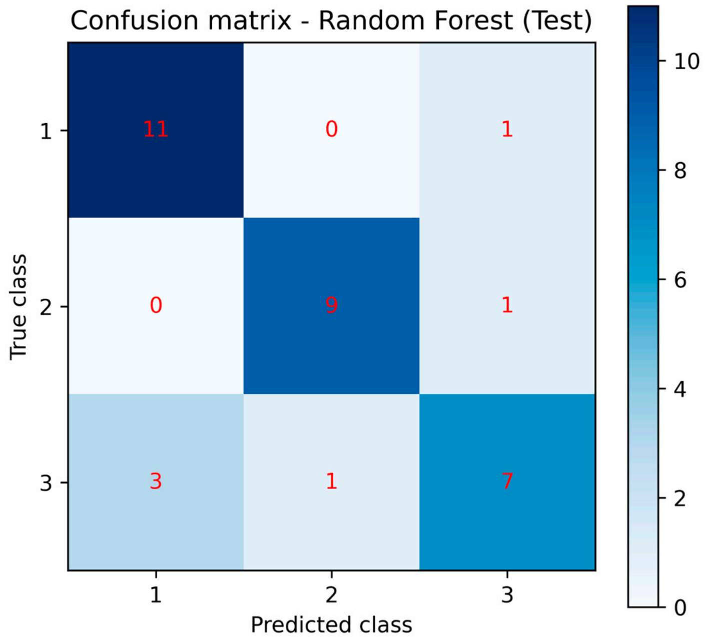

Figure S94. Confusion matrix of the classifier ensembles calculated on the test set for all sensory attributes of the odour

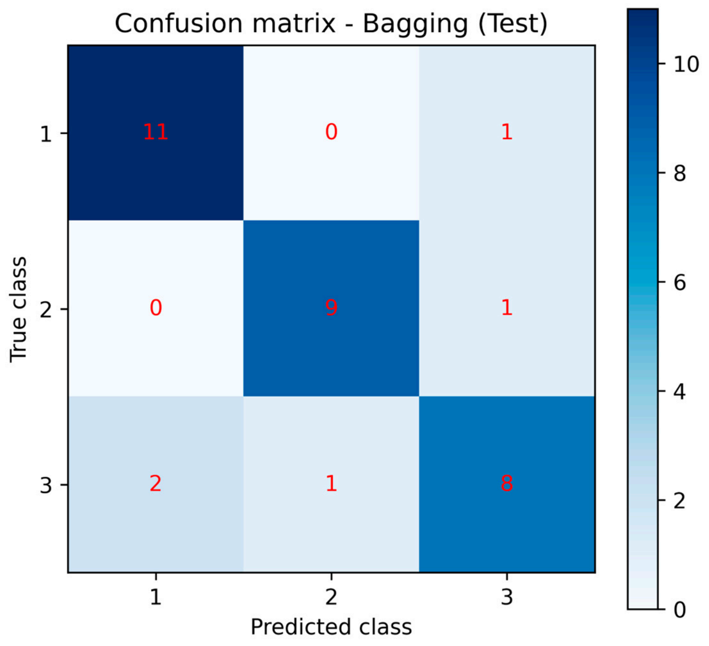

Figure S95. Confusion matrix of the classifier ensembles calculated on the test set for all sensory attributes of the odour

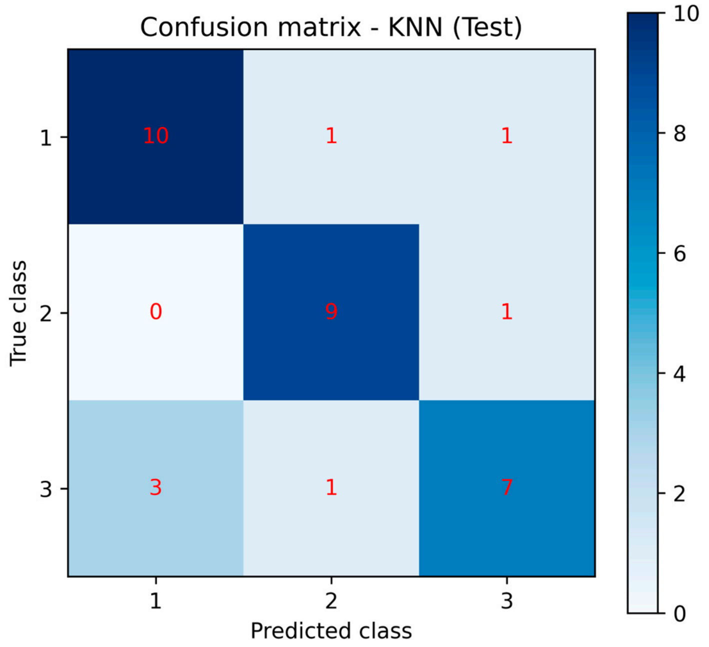

Figure S96. Confusion matrix of the classifier ensembles calculated on the test set for all sensory attributes of the odour

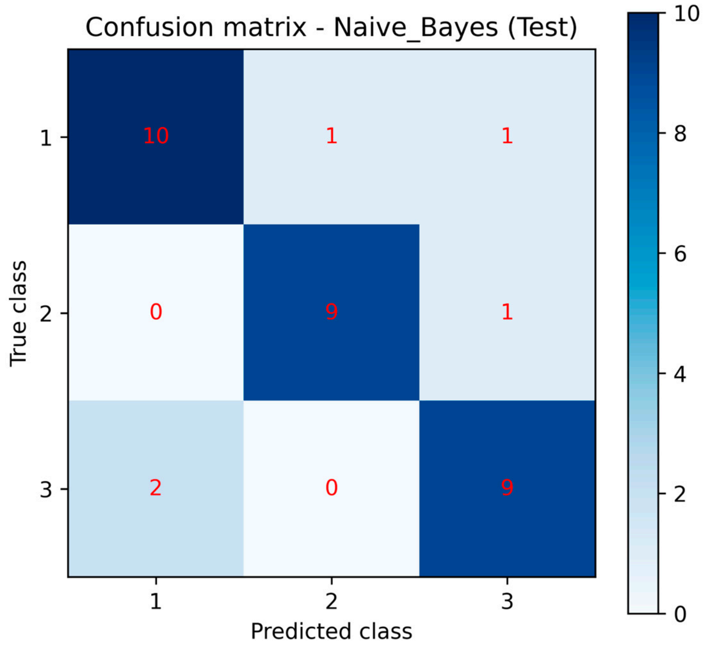

Figure S97. Confusion matrix of the classifier ensembles calculated on the test set for all sensory attributes of the odour

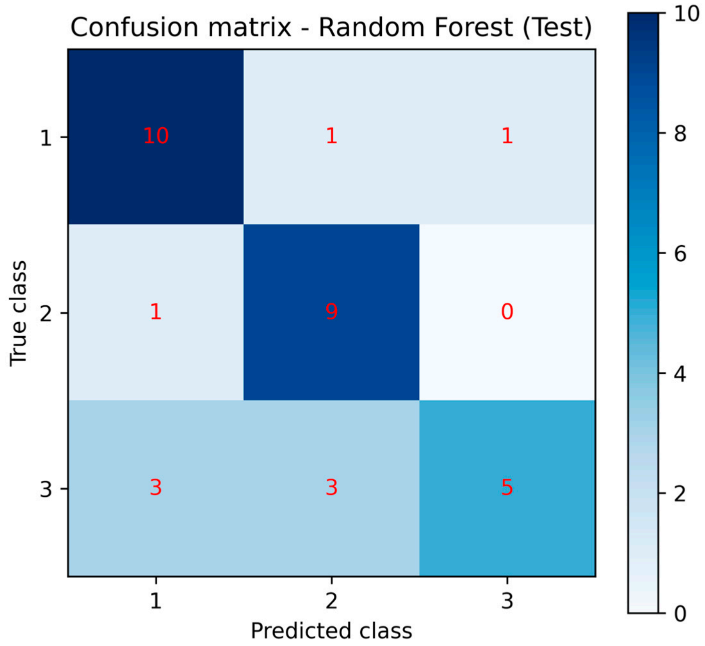

Figure S98. Confusion matrix of the classifier ensembles calculated on the test set for all sensory attributes of the odour

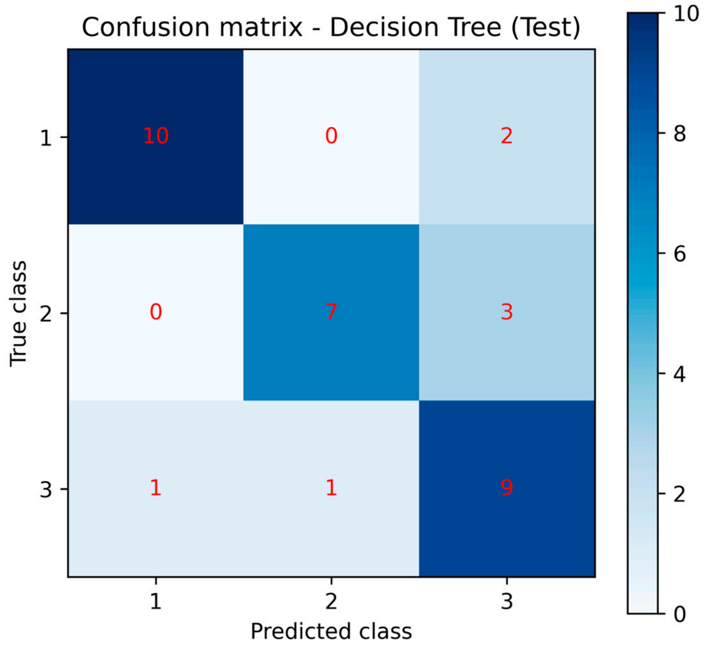

Figure S99. Confusion matrix of the classifier ensembles calculated on the test set for all sensory attributes of the odour

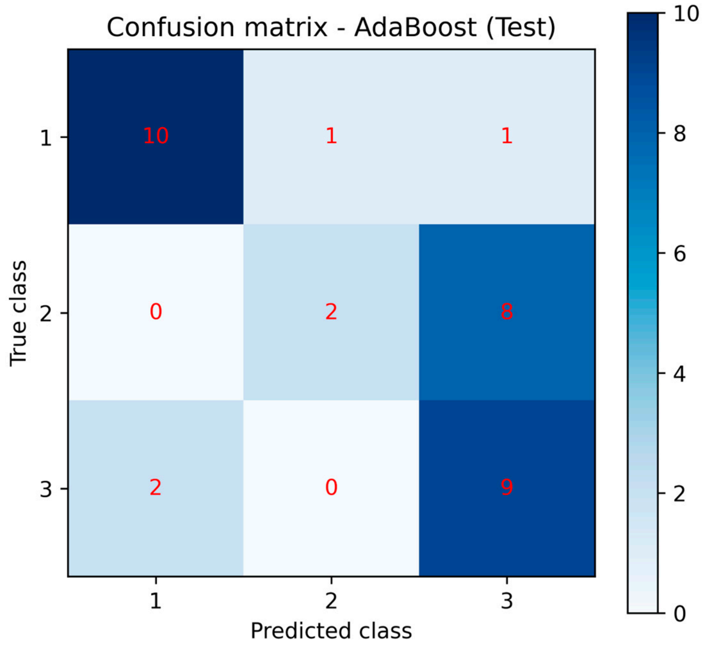

Figure S100. Confusion matrix of the classifier ensembles calculated on the test set for all sensory attributes of the odour

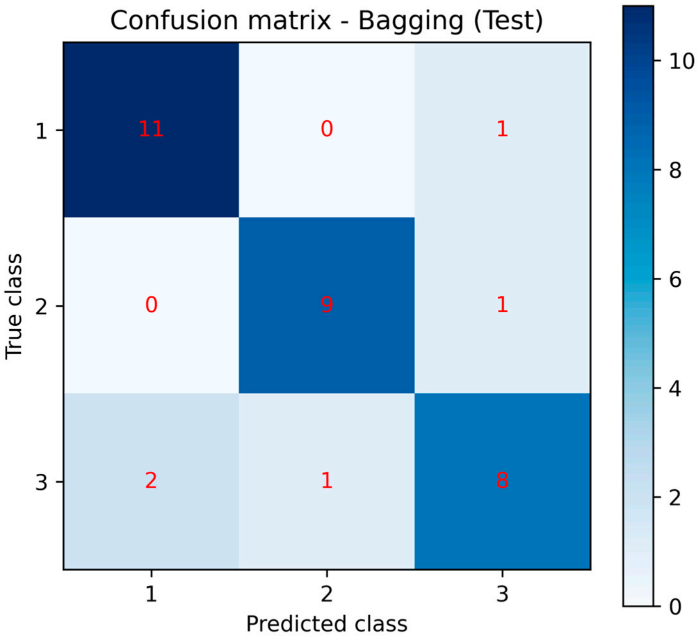

Figure S101. Confusion matrix of the classifier ensembles calculated on the test set for all sensory attributes of the odour

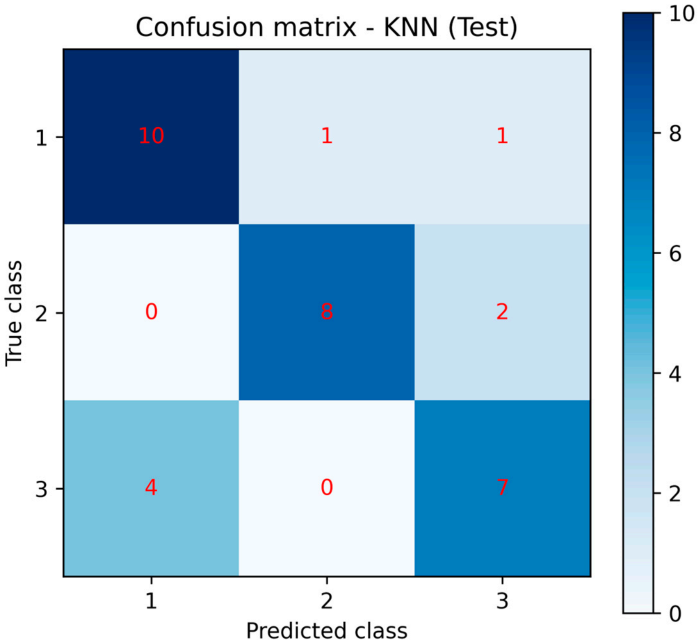

Figure S102. Confusion matrix of the classifier ensembles calculated on the test set for all sensory attributes of the odour

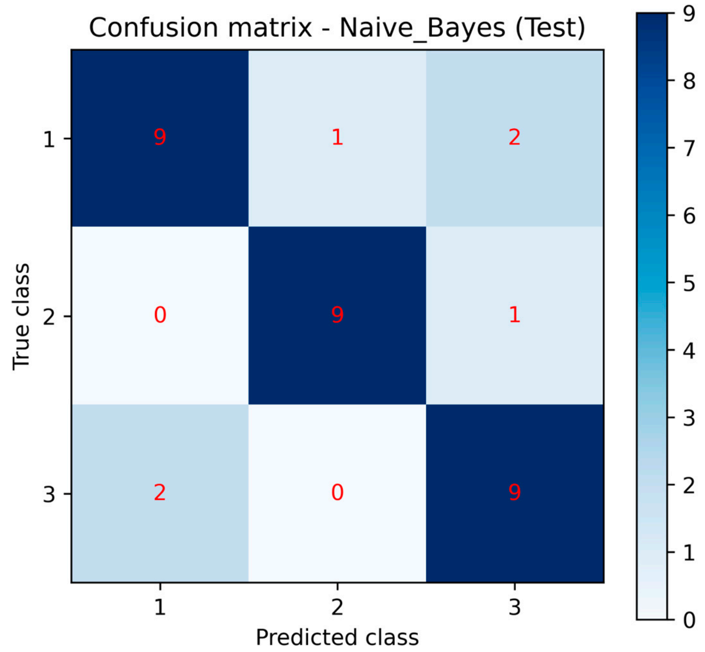

Figure S103. Confusion matrix of the classifier ensembles calculated on the test set for all sensory attributes of the odour

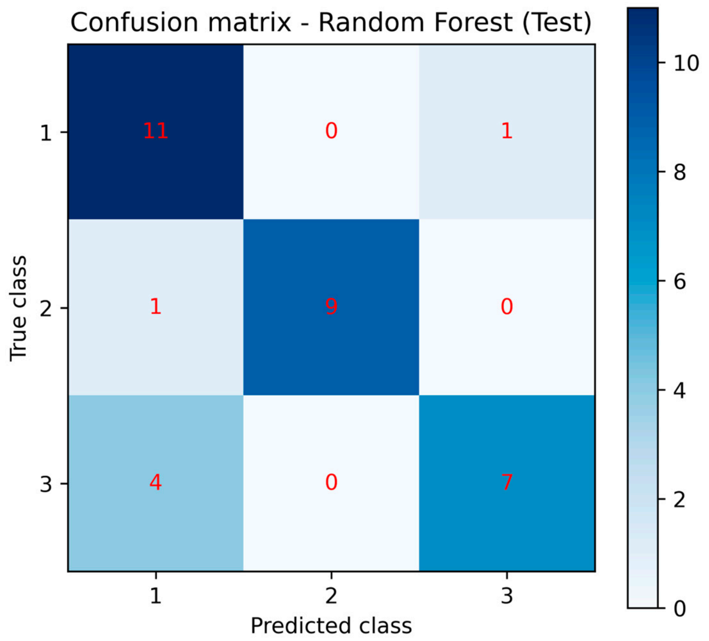

Figure S104. Confusion matrix of the classifier ensembles calculated on the test set for all sensory attributes of the odour

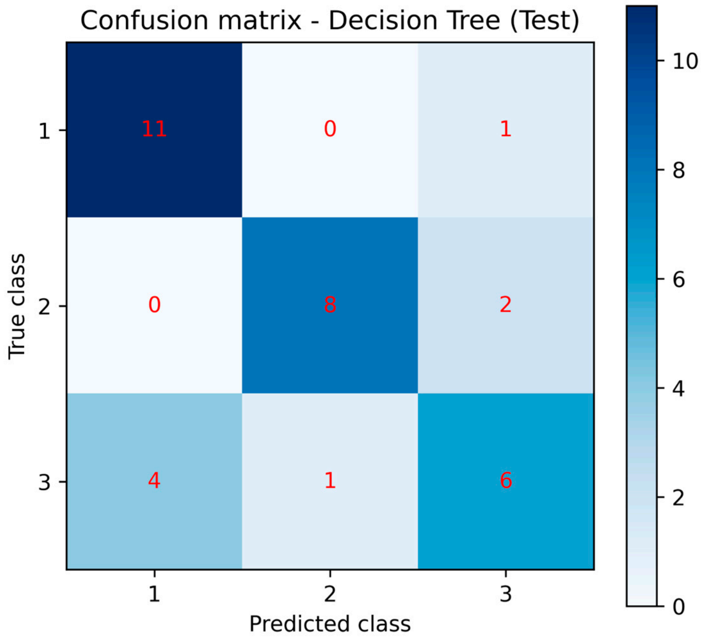

Figure S105. Confusion matrix of the classifier ensembles calculated on the test set for all sensory attributes of the odour

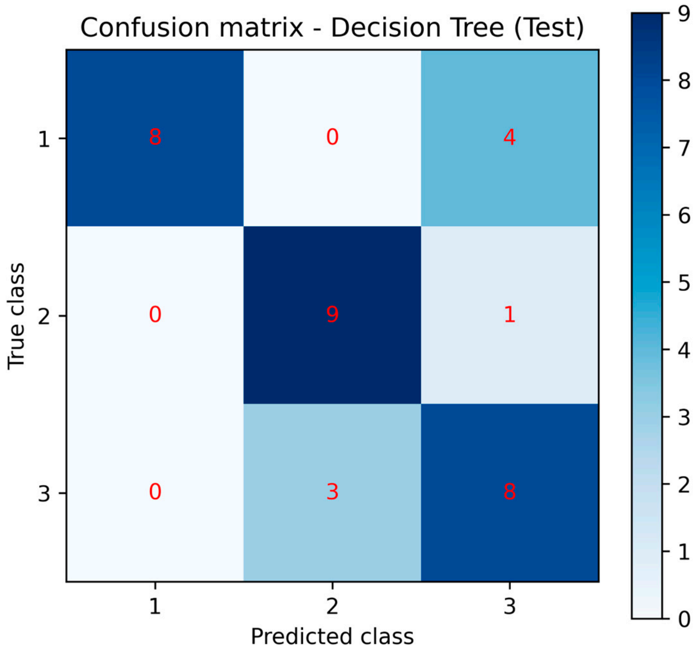

Figure S106. Confusion matrix of the classifier ensembles calculated on the test set for all sensory attributes of the odour

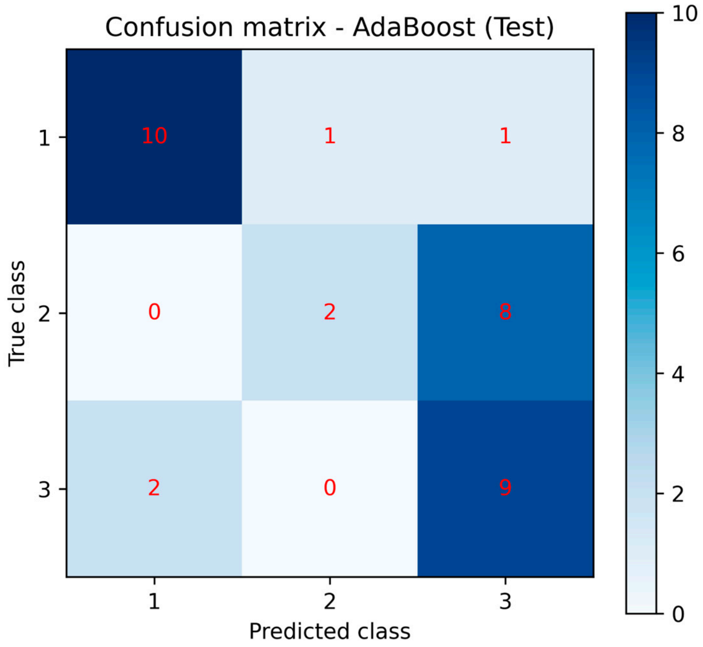

Figure S107. Confusion matrix of the classifier ensembles calculated on the test set for all sensory attributes of the odour

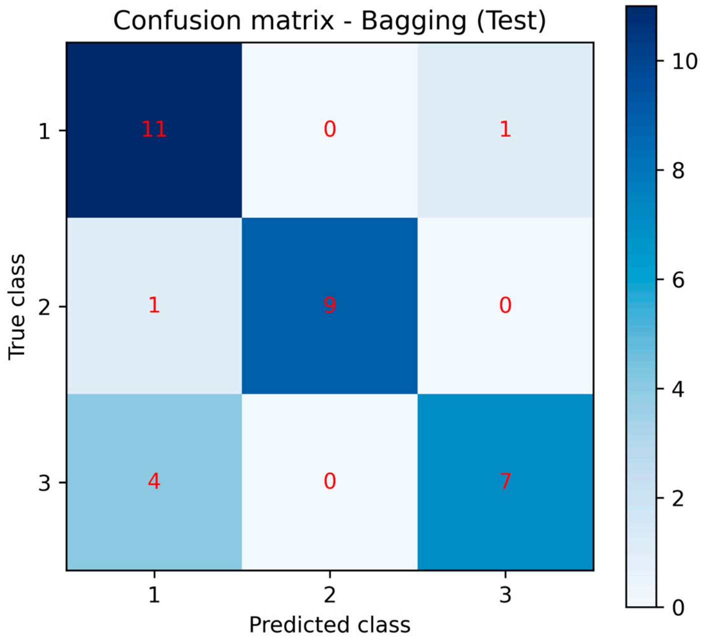

Figure S108. Confusion matrix of the classifier ensembles calculated on the test set for all sensory attributes of the odour

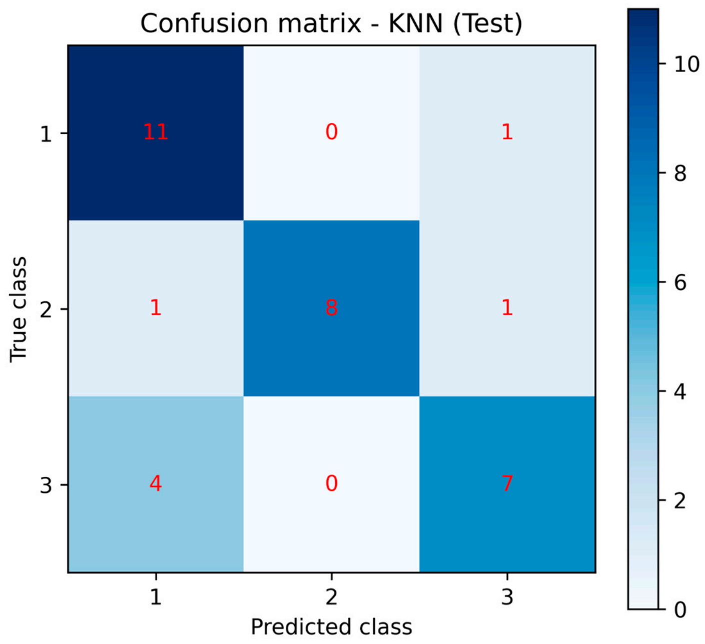

Figure S109. Confusion matrix of the classifier ensembles calculated on the test set for all sensory attributes of the odour

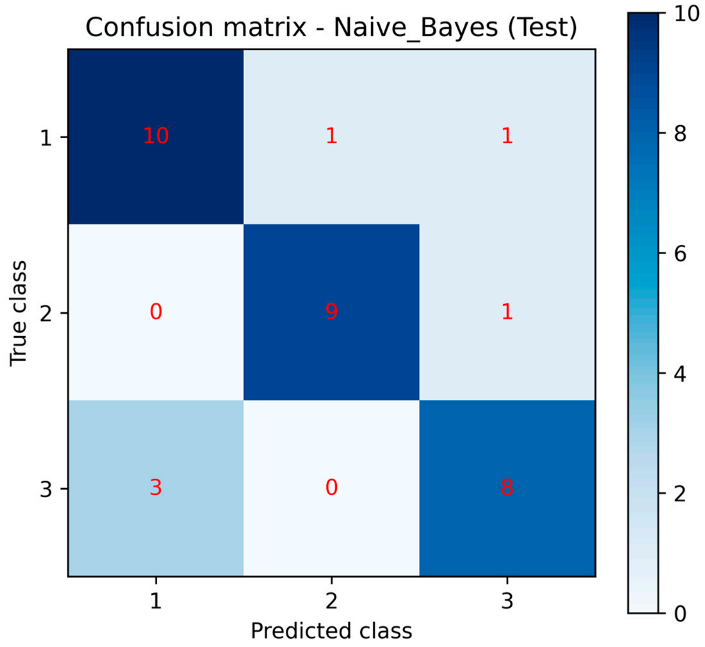

Figure S110. Confusion matrix of the classifier ensembles calculated on the test set for all sensory attributes of the odour

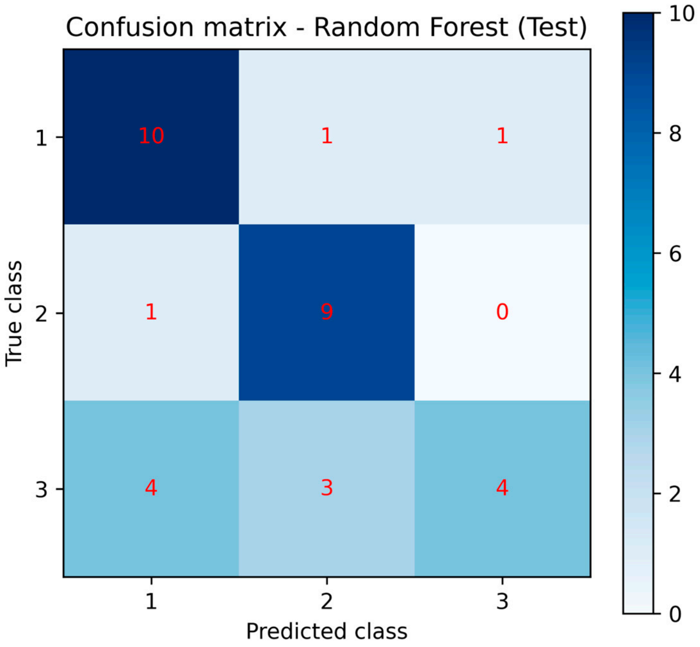

Figure S111. Confusion matrix of the classifier ensembles calculated on the test set for all sensory attributes of the odour

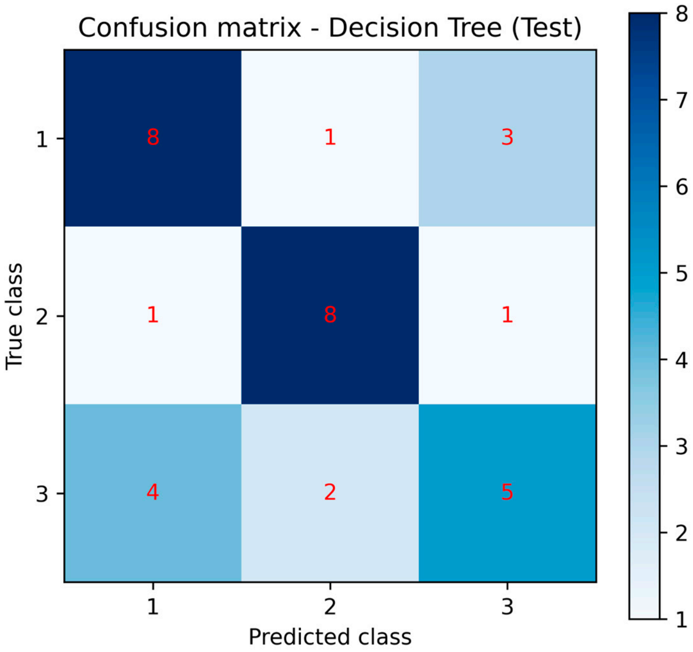

Figure S112. Confusion matrix of the classifier ensembles calculated on the test set for all sensory attributes of the odour

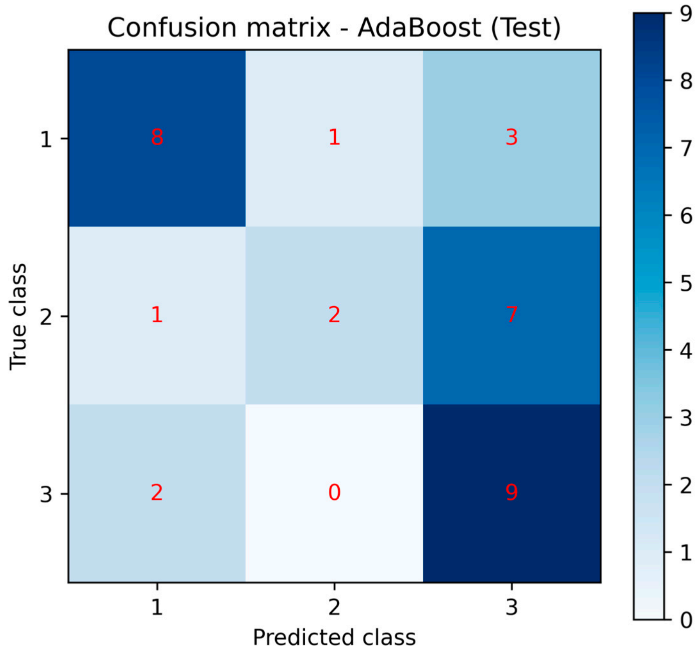

Figure S113. Confusion matrix of the classifier ensembles calculated on the test set for all sensory attributes of the odour

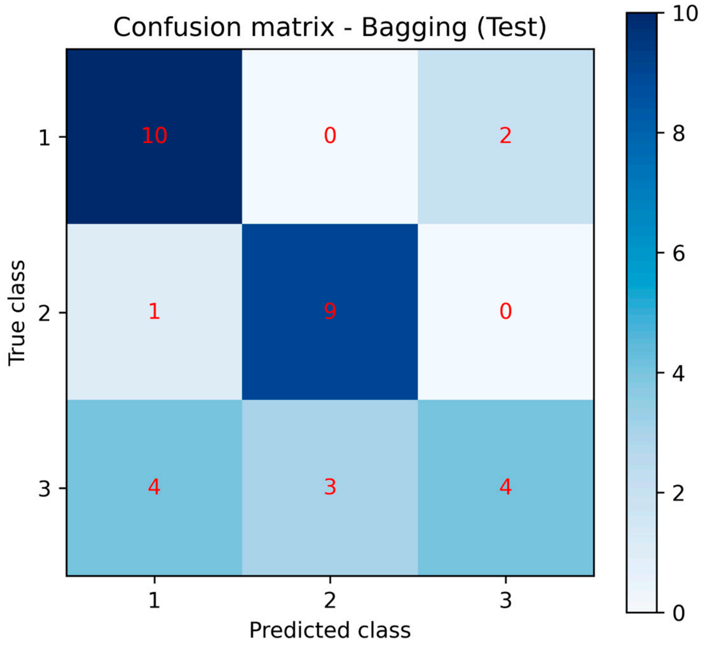

Figure S114. Confusion matrix of the classifier ensembles calculated on the test set for all sensory attributes of the odour

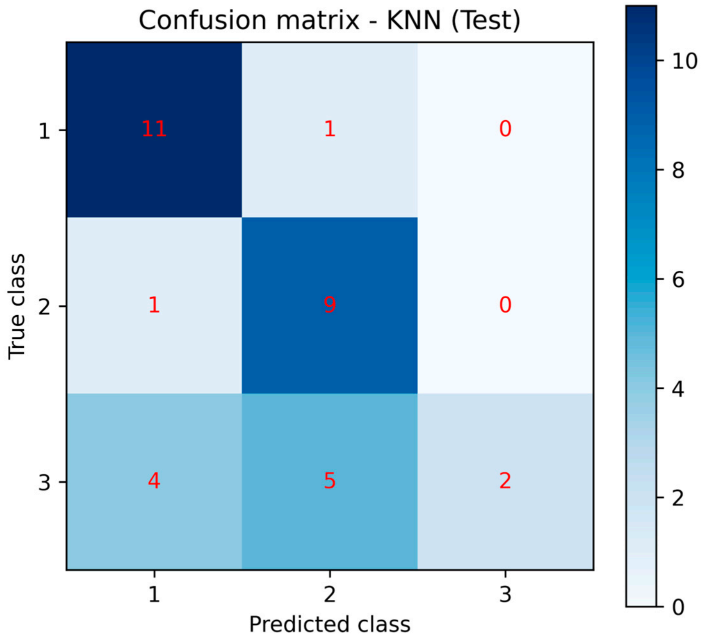

Figure S115. Confusion matrix of the classifier ensembles calculated on the test set for all sensory attributes of the odour

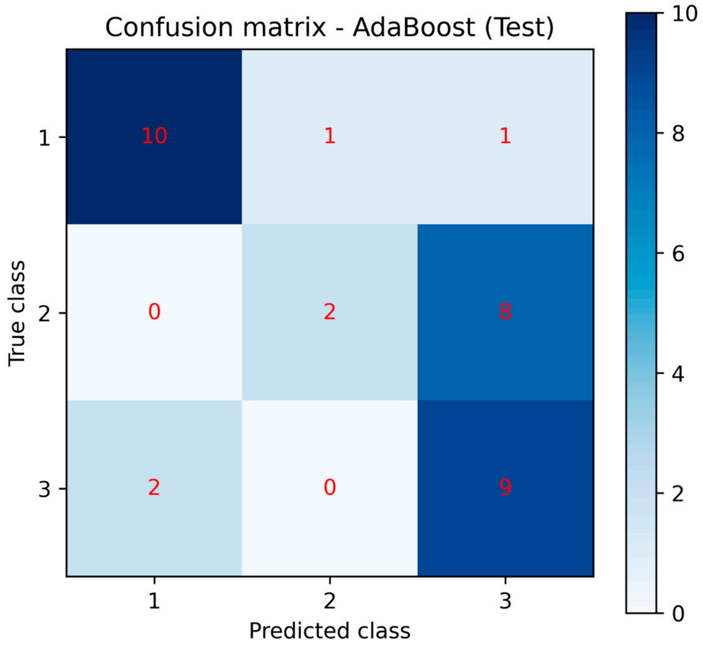

Figure S116. Confusion matrix of the classifier ensembles calculated on the test set for all sensory attributes of the odour

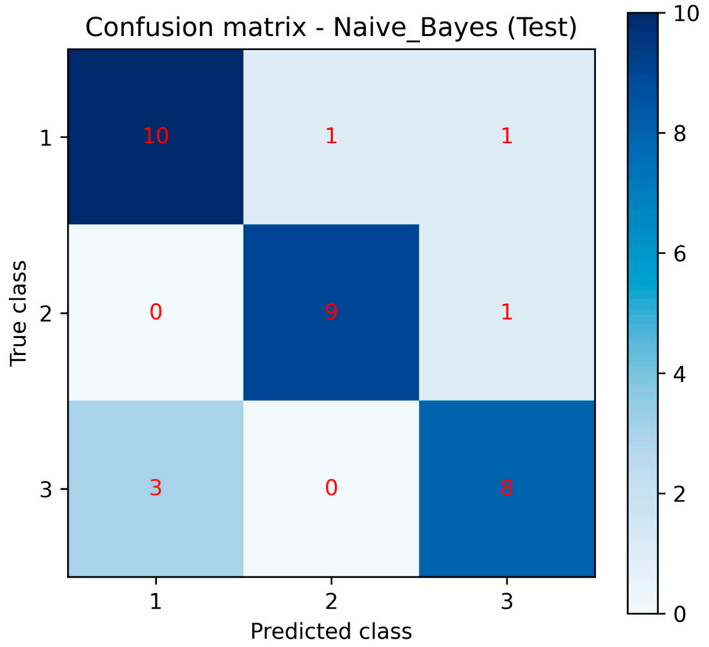

Figure S117. Confusion matrix of the classifier ensembles calculated on the test set for all sensory attributes of the odour

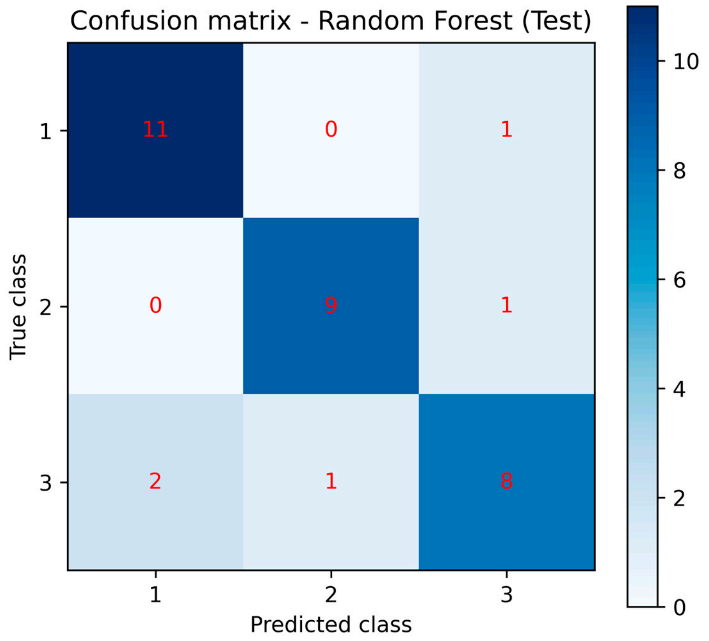

Figure S118. Confusion matrix of the classifier ensembles calculated on the test set for all sensory attributes of the odour

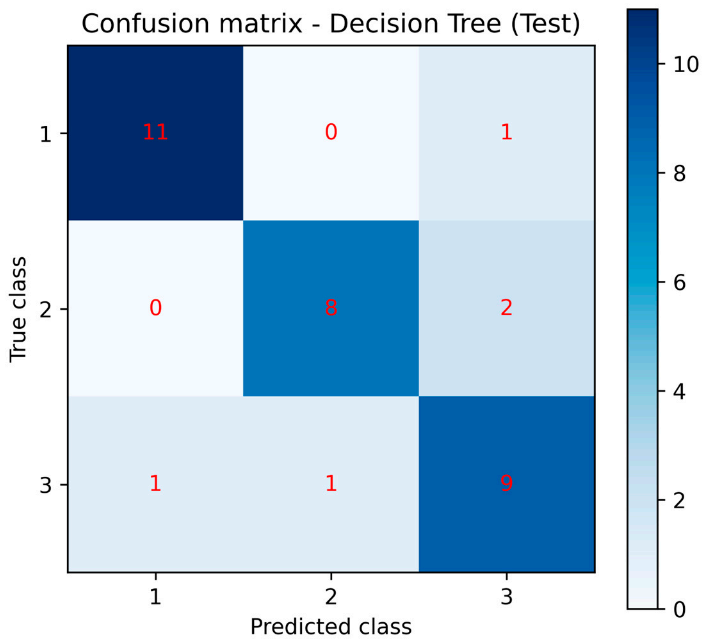

Figure S119. Confusion matrix of the classifier ensembles calculated on the test set for all sensory attributes of the odour

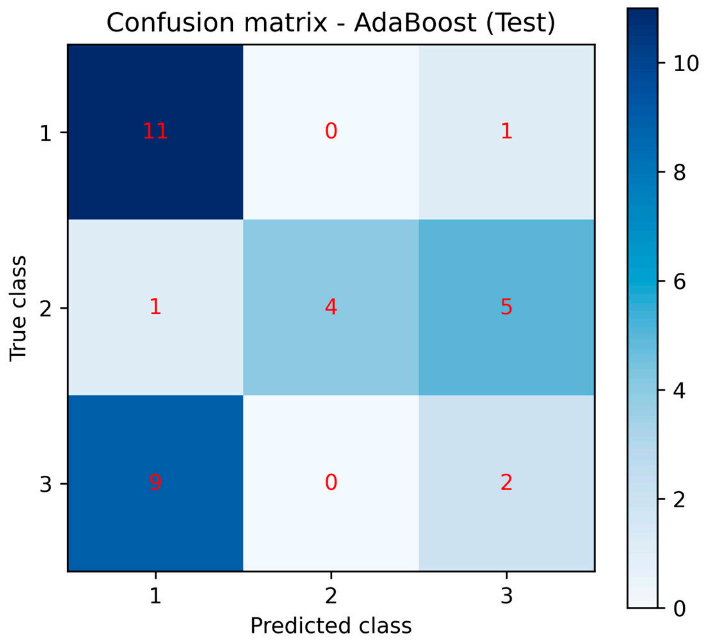

Figure S120. Confusion matrix of the classifier ensembles calculated on the test set for all sensory attributes of the odour

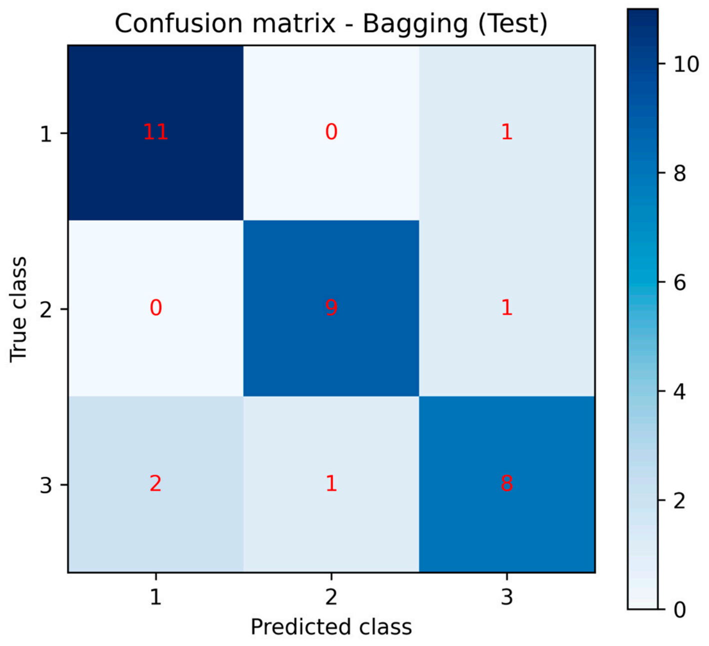

Figure S121. Confusion matrix of the classifier ensembles calculated on the test set for all sensory attributes of the odour

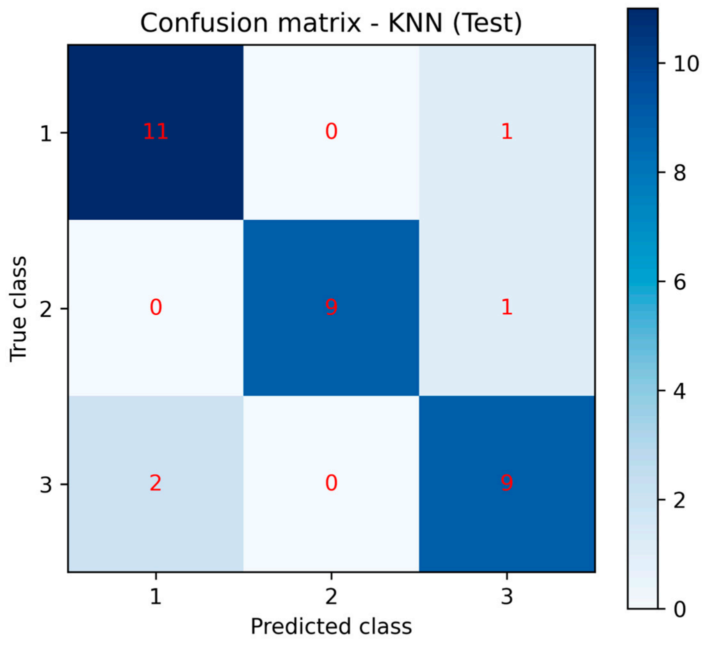

Figure S122. Confusion matrix of the classifier ensembles calculated on the test set for all sensory attributes of the odour

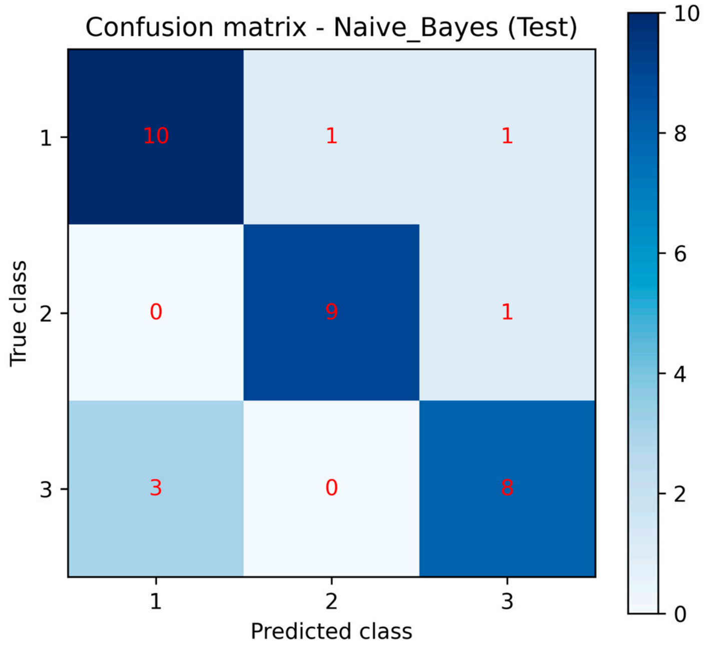

Figure S123. Confusion matrix of the classifier ensembles calculated on the test set for all sensory attributes of the odour

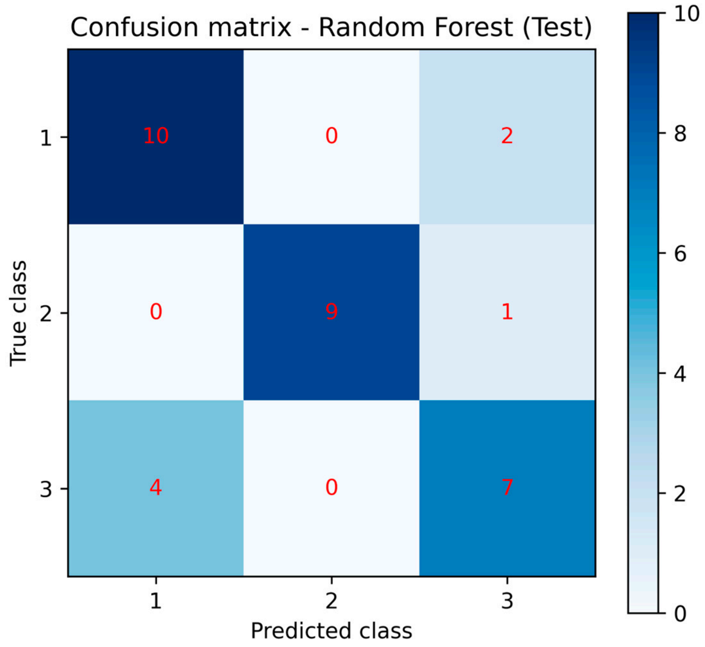

Figure S124. Confusion matrix of the classifier ensembles calculated on the test set for all sensory attributes of the odour

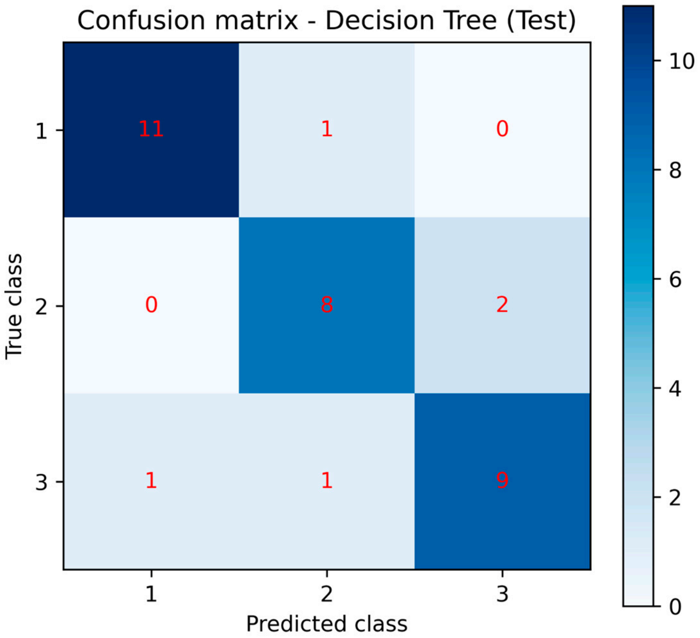

Figure S125. Confusion matrix of the classifier ensembles calculated on the test set for all sensory attributes of the odour

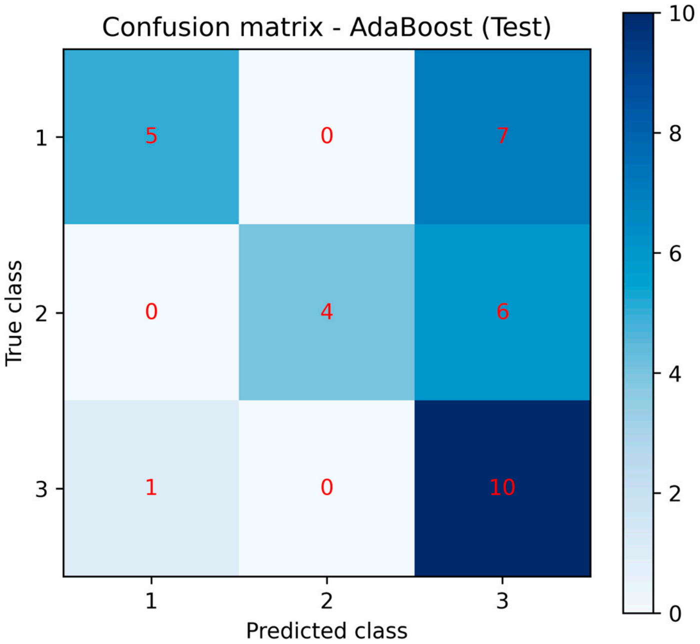

Figure S126. Confusion matrix of the classifier ensembles calculated on the test set for all sensory attributes of the odour
